# iPSCORE: A Resource of 222 iPSC Lines Enabling Functional Characterization of Genetic Variation across a Variety of Cell Types

**DOI:** 10.1016/j.stemcr.2017.03.012

**Published:** 2017-04-06

**Authors:** Athanasia D. Panopoulos, Matteo D'Antonio, Paola Benaglio, Roy Williams, Sherin I. Hashem, Bernhard M. Schuldt, Christopher DeBoever, Angelo D. Arias, Melvin Garcia, Bradley C. Nelson, Olivier Harismendy, David A. Jakubosky, Margaret K.R. Donovan, William W. Greenwald, KathyJean Farnam, Megan Cook, Victor Borja, Carl A. Miller, Jonathan D. Grinstein, Frauke Drees, Jonathan Okubo, Kenneth E. Diffenderfer, Yuriko Hishida, Veronica Modesto, Carl T. Dargitz, Rachel Feiring, Chang Zhao, Aitor Aguirre, Thomas J. McGarry, Hiroko Matsui, He Li, Joaquin Reyna, Fangwen Rao, Daniel T. O'Connor, Gene W. Yeo, Sylvia M. Evans, Neil C. Chi, Kristen Jepsen, Naoki Nariai, Franz-Josef Müller, Lawrence S.B. Goldstein, Juan Carlos Izpisua Belmonte, Eric Adler, Jeanne F. Loring, W. Travis Berggren, Agnieszka D'Antonio-Chronowska, Erin N. Smith, Kelly A. Frazer

**Affiliations:** 1 Gene Expression Laboratory, Salk Institute for Biological Studies, La Jolla, CA 92037, USA; 2 Institute for Genomic Medicine, University of California, San Diego, La Jolla, CA 92093, USA; 3 Department of Pediatrics, University of California, San Diego, La Jolla, CA 92093, USA; 4 Center for Regenerative Medicine, The Scripps Research Institute, La Jolla, CA 92037, USA; 5 Department of Medicine, University of California, San Diego, La Jolla, CA 92093, USA; 6 Zentrum für Integrative Psychiatrie, Universitätsklinikum Schleswig-Holstein, 24105 Kiel, Germany; 7 Bioinformatics and Systems Biology Graduate Program, University of California, San Diego, La Jolla, CA 92093, USA; 8 Biomedical Sciences Graduate Program, University of California, San Diego, La Jolla, CA 92093, USA; 9 Stem Cell Core, Salk Institute for Biological Studies, La Jolla, CA 92037, USA; 10 Department of Cellular and Molecular Medicine, University of California, San Diego, La Jolla, CA 92093, USA

**Keywords:** iPSCORE, iPSC, GWAS, molecular traits, physiological traits, cardiac disease, NHLBI Next Gen, LQT2, KCNH2, iPSC-derived cardiomyocytes

## Abstract

Large-scale collections of induced pluripotent stem cells (iPSCs) could serve as powerful model systems for examining how genetic variation affects biology and disease. Here we describe the iPSCORE resource: a collection of systematically derived and characterized iPSC lines from 222 ethnically diverse individuals that allows for both familial and association-based genetic studies. iPSCORE lines are pluripotent with high genomic integrity (no or low numbers of somatic copy-number variants) as determined using high-throughput RNA-sequencing and genotyping arrays, respectively. Using iPSCs from a family of individuals, we show that iPSC-derived cardiomyocytes demonstrate gene expression patterns that cluster by genetic background, and can be used to examine variants associated with physiological and disease phenotypes. The iPSCORE collection contains representative individuals for risk and non-risk alleles for 95% of SNPs associated with human phenotypes through genome-wide association studies. Our study demonstrates the utility of iPSCORE for examining how genetic variants influence molecular and physiological traits in iPSCs and derived cell lines.

## Highlights

• iPSCORE: A collection of publicly available iPSCs from 222 individuals
• Several multigenerational families and individuals of various ethnicities and ages
• Individuals carrying risk and non-risk genotypes for 95% of GWAS SNPs
• Genetic variants associated with mRNA expression in differentiated cardiomyocytes

## Graphical Abstract

**Figure:**
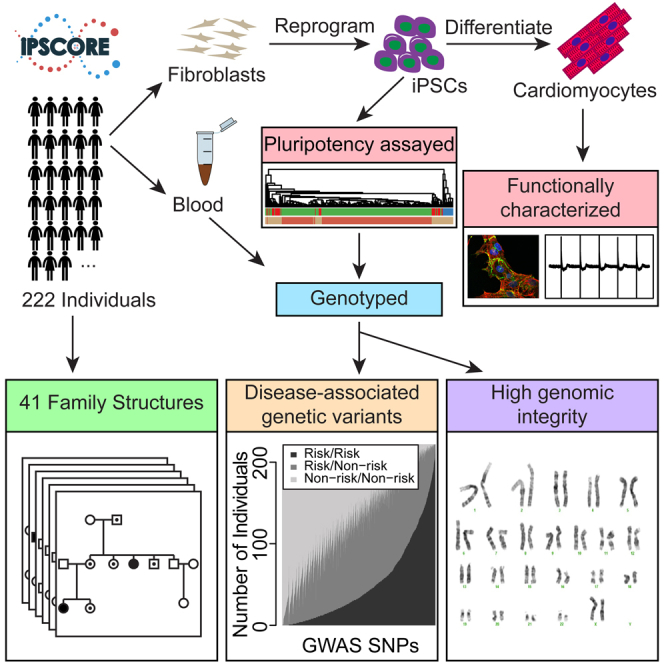

## Teaser

Working as part of the NHLBI NextGen consortium, Panopoulos and colleagues report the derivation and characterization of 222 publicly available iPSCs from ethnically diverse individuals with corresponding genomic data including SNP arrays, RNA-seq, and whole-genome sequencing. This collection provides a powerful resource to investigate the function of genetic variants.

## Introduction

Due to their ability to differentiate into a variety of cell types, induced pluripotent stem cells (iPSCs) are a potentially powerful model system to study mechanisms underlying non-coding genetic variants associated with human traits, many of which lie in cell-type-specific regulatory regions (Maurano et al., 2012). However, because non-coding regulatory variants can have relatively small effect sizes, hundreds of lines from diverse individuals may be needed to measure genetic associations as opposed to the tens of different lines typically used to study disease-associated coding variants with strong effects (Avior et al., 2016). To enable the study of genetic variants associated with complex diseases and cell-type-specific molecular phenotypes, we and others are establishing large systematically generated collections of iPSCs toward the goal of generating large genomic datasets that will be openly available to researchers (Avior et al., 2016, Kilpinen et al., 2016, McKernan and Watt, 2013, Streeter et al., 2017). Ongoing collections, including large disease-focused iPSC repositories (www.cirm.ca.gov), however, are currently limited in sample diversity and in related individuals (e.g., pedigrees or twins), which would allow for the interrogation of population-associated genetic variation, rare variation, and family-based genetic study designs. Thus, the generation of a resource consisting of hundreds of systematically derived iPSCs with available genomic data including SNP arrays, RNA sequencing (RNA-seq), and whole-genome sequencing, and that includes a variety of familial architectures and individuals of multiple ethnicities, would further enable a wide variety of study designs to interrogate the genetic basis of phenotype and disease.

There are a number of potential challenges to using iPSC and iPSC-derived cells to model human phenotype and disease. Somatic heterogeneity in iPSC lines that can occur during isolation and culture may interfere with examining genetic variants with subtle effects (Fusaki et al., 2009, International Stem Cell et al., 2011, Nazor et al., 2012). This heterogeneity can include copy-number alterations, which have been reported as occurring in recurrently altered regions in existing collections of pluripotent stem cells (both embryonic stem cells [ESCs] and iPSCs) (International Stem Cell et al., 2011, Laurent et al., 2011, Taapken et al., 2011). However, because many of these lines were not systematically generated and may have undergone prolonged passaging in culture, it is unclear how prevalent these hotspots are in limited passaged lines and/or if other hotspots could be uncovered as additional iPSC are examined. In addition, it is not yet known whether iPSC-derived cell types (cardiomyocytes, neurons, adipocytes) will be useful for functionally examining genetic variants. We and others have recently shown that genetic differences between individuals are associated with a variety of molecular phenotypes in iPSCs, including the transcriptome and epigenome (Burrows et al., 2016, DeBoever et al., 2017, Panopoulos et al., 2017, Rouhani et al., 2014, Thomas et al., 2015), but it is still unclear whether genetic background is associated with molecular phenotypes in iPSC-derived cells.

Here, we describe the iPSCORE (iPSC Collection for Omic Research) resource, a systematically derived and characterized reference panel of iPSC lines. Participants were recruited to include families, twins, and individuals of diverse ethnicity to enable genetic studies investigating the segregation of traits. While the majority of the participants were generally healthy, 39 individuals with heart diseases were included to allow for investigations into heart disease using derived cell types. iPSCs were systematically reprogrammed from fibroblasts and analyzed for pluripotency and the presence and recurrence of somatic copy-number variants (CNVs). We differentiated a subset of iPSCs to cardiomyocytes and examined how the donor's genetic background is associated with gene expression variation in derived cell lines. Finally, we examined and annotated how individuals in the iPSCORE resource carry SNPs associated with diverse genome-wide association studies (GWAS) phenotypes. The iPSCORE resource provides a powerful tool to examine how genetic variants influence molecular and physiological traits across a variety of derived cell types, as well as to functionally interrogate variants underlying a variety of GWAS phenotypes.

## Results

### Recruitment and Characterization of Individuals in the iPSCORE Resource

**Figure 1:**
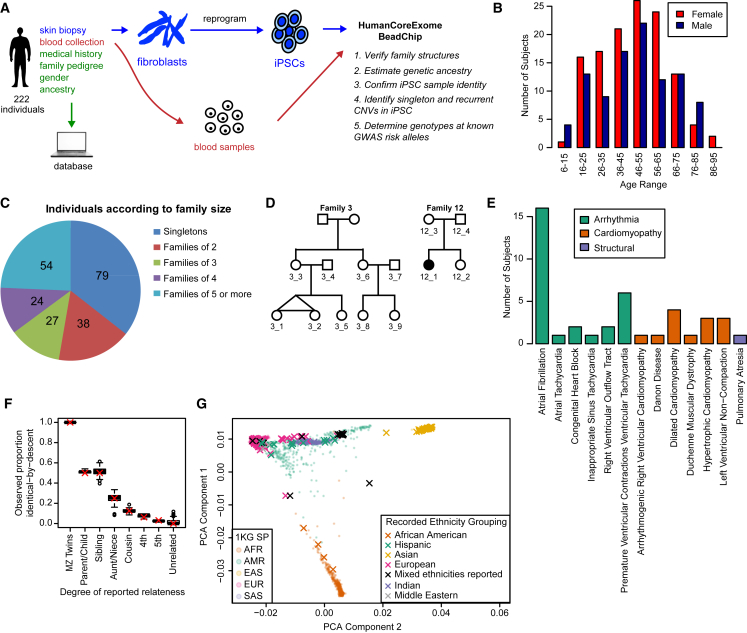
Description of the iPSCORE Cohort (A) Pipeline for the systematic generation and characterization of 222 iPSC lines. Individuals filled out a questionnaire detailing their medical history, family relationships to other subjects in the cohort, gender, and ancestry. Fibroblasts from skin biopsy were reprogrammed to integration-free iPSC using Sendai virus and frozen at passage 12. Genomic DNA isolated from the iPSC and the subject-matched blood samples were hybridized to the HumanCoreExome array. The resulting data were then used to confirm reported family structure, reported ancestry, and iPSC sample identity (match with blood sample), and to perform CNV analysis (iPSC characterization) and determine status of known disease risk alleles. (B) Age distributions of males and females. (C) Pie chart showing how many individuals are singletons or in a family size of 2, 3, 4, and 5 or more. (D) Pedigrees of two representative families; numbered individuals indicate presence in the study. Family 3 is a two-generation family with identical twins (nine subjects), and Family 12 has a member diagnosed with ventricular tachycardia and congenital heart block (four subjects). (E) Number of individuals with cardiac disease, grouped by disease type. Some individuals are affected by multiple types of arrhythmia. (F) Boxplot showing the observed proportion of the genome identical by descent (pIBD) as a function of the reported family relationship. The box hinges indicate the 25^th^ and 75^th^ quantiles and the whiskers extend to 1.5 times the interquartile range. A red “X” indicates the expected mean pIBD given the number of generations that separate the individuals. (G) An x-y plot showing the first versus second components of a principal component analysis using genotype data from a subset of SNPs present on the array mapped onto a principal component analysis from the 1,000 Genomes Project (1KG) super populations (SP) (small faded circles). Individuals from the iPSCORE cohort are mapped onto these components with their recorded ethnicity grouping shown by a colored X.

We recruited individuals and recorded sex, age, medical history, ethnicity, and relatedness to others in the collection through a questionnaire at enrollment (Figure 1A). Hereafter, we describe the 222 individuals for which we successfully obtained at least one iPSC line (Table S1). There were 124 females ranging in age from 10 to 88 (median age 48), and 98 males ranging in age from 9 to 82 years of age (median age 49) (Figure 1B). The resource includes 143 participants who are members of a family and genetically related to at least one other individual (Figures 1C and S1). In total, there are 41 families that contain between 2 and 14 members, which include seven monozygotic twin pairs and two dizygotic twin pairs (example pedigrees in Figure 1D; see Figure S1 for all pedigrees). Due to the fact that some of the individuals in the 41 families are only related by marriage, there are a total of 136 genetically unrelated individuals in the collection. While most participants in the collection do not have heart disease, there were 25 individuals with arrhythmia (some with multiple types), 13 with cardiomyopathy, and one with structural cardiac malformations (Figure 1E and Table S1A). Using whole-genome sequence data generated from the blood of cardiac disease probands and their families (DeBoever et al., 2017), we examined genetic variation at candidate disease genes and identified four potentially disease-associated variants affecting two families and two singletons (Table S1B). Overall, the iPSCORE resource contains both complex family structures and unrelated individuals across a large spectrum of ages and multiple ethnicities predominantly from healthy donors, but also includes a subset (18%) of individuals that have a diagnosed cardiac disease.

Germline DNA isolated from blood (or in 16 cases from fibroblasts) from each participant was hybridized to the HumanCoreExome BeadChip, and we used the derived genotypes to confirm reported familial relationships, ancestry, and sex. We estimated the proportion of the genome identical-by-descent between each pair of germline samples and observed genetic similarity that was consistent with reported familial relationships, with no cryptically related individuals (Figure 1F). Ethnicities were recorded as free response by the participants (or in a minority of cases, the physician) and categorized into the following “recorded ethnic groups” (number of individuals given): African American (4), Hispanic (15), Asian (30), European (147), Multiple ethnicities reported (18), Indian (6), and Middle Eastern (2). We estimated genetic ancestry by comparing the genetic similarity of the participants to the 1,000 Genomes Project (1KGP) and observed 100% concordance with the reported ethnicity and the most similar 1KGP super population using linear discriminant analysis (Figure 1G and Table S1A). However, some heterogeneity was observed in clustering of the first principal components, consistent with some level of unreported admixture. Finally, sex was determined from genotype data and no discrepancies were identified. These results suggest that the samples analyzed are consistent with reported phenotypes and familial relationships.

### Generation, Sample Identity Verification, and Pluripotency Testing of iPSC Lines

Skin biopsies collected at enrollment were immediately used to derive fibroblasts for generating iPSCs, while the blood was stored for later DNA extraction (Figure 1A). We used a non-integrative reprogramming method (Sendai virus) to generate the iPSCs and derived multiple clones from each individual (on average three clones), with a minimum of two clones frozen at passage 3 (P3) and at least one clone cultured to later passage (typically P12). We attempted to reprogram fibroblasts from 240 of the recruited participants and obtained iPSCs for 224 individuals, of which 222 passed sample identity quality control (see below).

To confirm sample identity of the iPSC, we hybridized DNA isolated from the iPSC samples (typically at P12) to HumanCoreExome BeadChips and compared it with the genotype data from the matched germline sample. Sample identity was considered confirmed if the iPSC line genetically matched the donor germline sample across 90,099 SNPs. We identified two iPSC lines that did not genetically match the blood sample: for one, we suspect that the blood was mislabeled at time of collection, and for the other, that the iPSC was exchanged with another unknown cell line. In both cases, the anomalous sample did not match with any other sample in the study, and both germline/iPSC pairs were excluded. Overall, 222 of 224 (99.1%) iPSC lines passed sample identity quality control and were included in the study.

**Figure 2:**
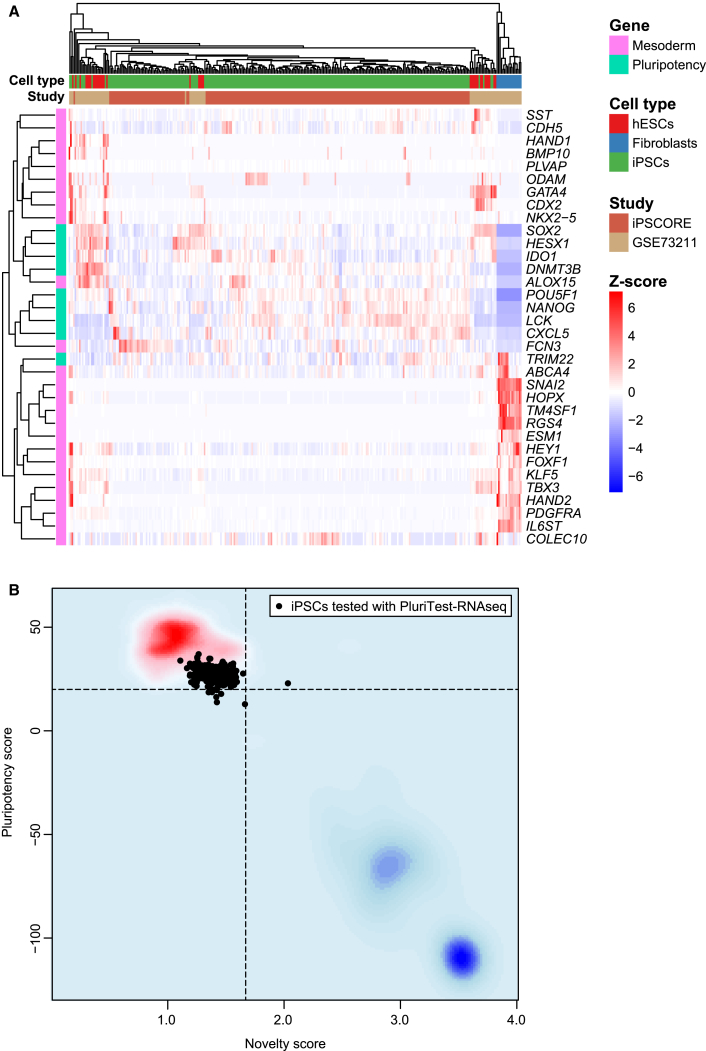
Analysis of iPSC Transcriptome Data to Assess Pluripotency (A) Heatmap and hierarchical clustering showing normalized expression levels (*Z* scores derived from VST expression levels) of nine pluripotency (green) (Burridge et al., 2012, Dubois et al., 2011, Vidarsson et al., 2010) and 25 mesoderm marker genes (pink) (Tsankov et al., 2015) in 213 iPSCORE iPSC lines and 73 cell lines (21 iPSC, 35 hESC, and 17 fibroblast) obtained from GEO: GSE73211 (Choi et al., 2015). Samples are color coded to show whether they are derived from iPSCORE (dark brown) or from GEO: GSE73211 (light brown), and on the basis of tissue type (red for hESC, green for iPSC, and blue for fibroblast). The heatmap shows that iPSCs and hESCs have higher overall expression of pluripotency genes than fibroblasts, which have low expression of pluripotency genes, but higher expression of most mesoderm markers than iPSC lines and hESC lines. (B) PluriTest-RNAseq-based analysis of 213 iPSCORE lines (green) with RNA-seq data. The red and blue background encodes an empirical density map indicating the location of pluripotent (red) and non-pluripotent (blue) cells in the reference dataset. The x axis represents novelty score, which indicates how much the test iPSC deviates from a normal pluripotent line, with higher values being associated with more somatic characteristics and therefore lower pluripotency. The y axis represents the pluripotency score, a logistic regression model that enables a probability-based choice between pluripotent and non-pluripotent classes (Muller et al., 2011).

To evaluate iPSC pluripotency, we conducted flow cytometry and analyzed gene expression using expression arrays and RNA-seq data. We examined a subset of the lines (50 samples) by flow cytometry, all of which showed >95% positive staining for the pluripotency markers Tra-1-81 and SSEA-4 (Figure S2). For 213 iPSCs with RNA-seq (DeBoever et al., 2017), we compared the expression levels of nine pluripotency (Burridge et al., 2012, Dubois et al., 2011, Vidarsson et al., 2010) and 25 mesoderm markers (Tsankov et al., 2015) to publicly available RNA-seq data from human ESCs (hESCs), iPSCs, and fibroblasts (Choi et al., 2015) (Figure 2A). The iPSCs were comparable with these previously established pluripotent stem cell lines, showing low expression of mesoderm markers and high expression of pluripotency markers (Figure 2A). To further examine iPSC pluripotency, we analyzed the RNA-seq expression data for the 213 lines using PluriTest-RNAseq, a recently modified version of PluriTest (Muller et al., 2011) that has been adapted for RNA-seq (see Supplemental Experimental Procedures; unpublished data by B.M.S., R.W., F.J.M., and J.F.L.) as opposed to gene expression arrays. We observed strong clustering of the iPSCs in the upper left quadrant with 206 of the lines passing the test's criteria (>20 Pluripotency Score, indicating high expression levels of pluripotency-associated gene signatures; <1.67 Novelty, indicating a low probability of epigenetic or genetic abnormalities) (Table S2 and Figure 2B). Of the seven outliers, four have normal karyotypes and three have CNVs that cumulatively account for less than 500 kb in total length per line (see below and Table S3), suggesting that the variation in score is not due to genetic abnormalities. As part of an ongoing project whereby we are differentiating these iPSC lines into cardiomyocytes (see below), we attempted to differentiate four of the outlying samples and successfully differentiated three, which is similar to the overall ∼78% success rate (147 of 188 attempted) for first cardiac differentiation attempts, indicating that these outliers show differentiation rates similar to passing lines (data not shown). Thus, these results support that the iPSCORE lines are pluripotent.

### Characterization of Somatic Copy-Number Variants

**Figure 3:**
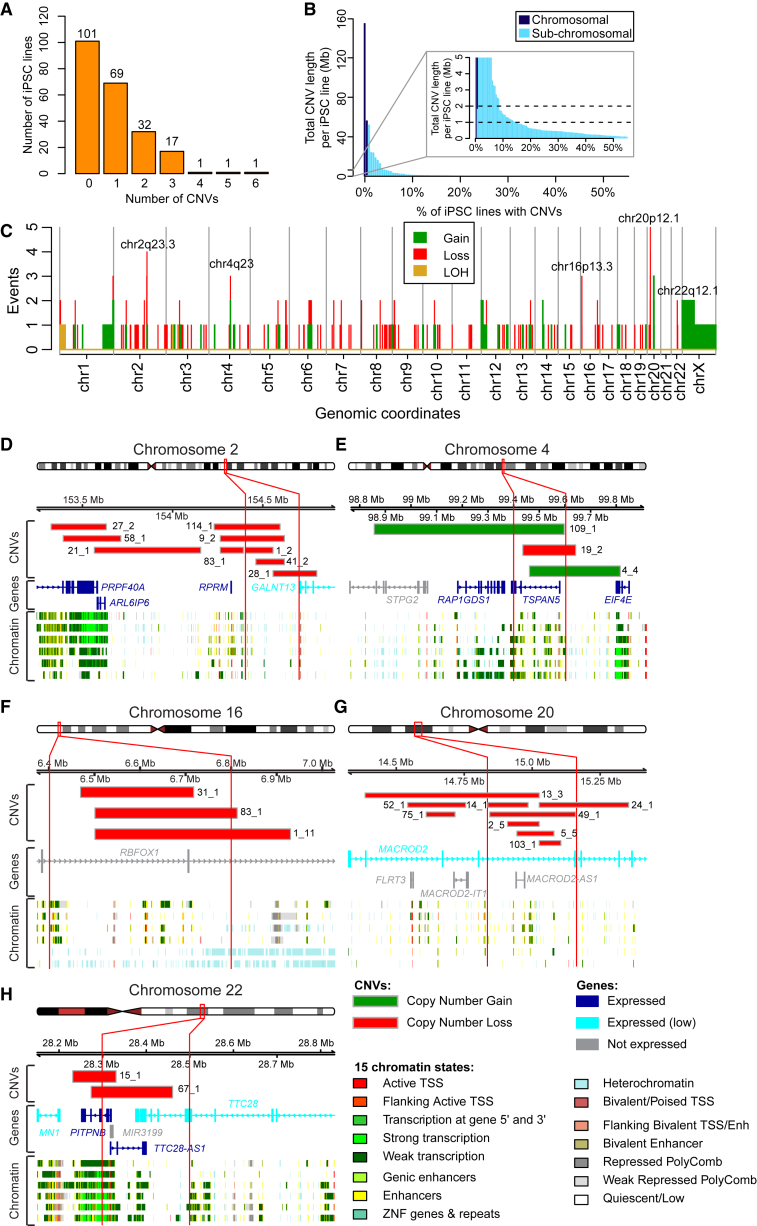
Characteristics of the Copy-Number Variants in the 222 iPSC Cell Lines (A) Histogram showing the number of iPSC cell lines with (N) detected CNV aberrations (x axis); for example, 101 of the iPSC lines have zero aberrations detected. (B) Histogram showing the cumulative size of CNVs (in megabase pairs) per iPSC cell line as a percentage of the cohort (Total = 222). (C) Histogram showing the number of alterations in each genomic locus. The five intervals harboring a significant cluster of CNVs are indicated. Gray lines separate chromosomes. (D–H) Genomic intervals harboring significantly clustered CNVs. The relative chromosomal position of each CNV cluster is shown. Red vertical lines delineate the regions significantly enriched, but additional nearby CNVs are also shown. In each panel, expressed and non-expressed genes are color coded. RNA-seq data of the 213 iPSC lines were used to determine gene expression levels: genes were defined as not expressed (gray) if fewer than 10 iPSC had an expression level of transcripts per million (TPM) >2; genes with a mean TPM <4 were considered as having low expression (light blue); while genes with a mean TPM >= 4 were considered as expressed (dark blue). The rows underneath the genes show 15 chromatin states in one ESC line (ESC.4STAR; top row) and five iPSC lines derived from Roadmap ChromHMM (http://egg2.wustl.edu/roadmap/ [Ernst and Kellis, 2012, Roadmap Epigenomics et al., 2015]). (D) Nine samples (sample ID indicated) harbor deletions (red rectangles) at chr2q23.3, of which five fall in the significantly enriched region. (E) Two samples harbor gains and one harbors a loss at chr4q23. (F) Three samples harbor deletions at chr16p13.3. (G) Nine samples harbor deletions at chr20p12.1, of which seven fall in the associated region. (H) Two samples harbor deletions at chr 22q12.1.

Previous studies have shown that iPSC lines can contain somatic CNVs that were either present in the donor sample or arose during/after the reprogramming process (Abyzov et al., 2012, Hussein et al., 2011, Hussein et al., 2013, Young et al., 2012). To examine the genomic integrity of the iPSC lines, we compared the intensity levels and B-allele frequencies of the HumanCoreExome arrays between the matched germline and iPSC DNA samples. We used a visual approach and a paired analysis in Nexus CN, a method that requires iPSC variants to be different from germline, and thus excludes inherited CNVs (see Supplemental Experimental Procedures). We identified 199 regions from 121 cell lines that met our criteria for CNVs with high confidence (listed in Table S3A and Figure S3). Notably, 101 of the 222 iPSC lines (as scored by the criteria described here) have no significant CNVs when compared back with their corresponding germline sample. This is followed by a distribution of iPSCs having between one CNV (69 lines) and six CNVs (1 line) (Figure 3A). We observed one trisomy (chromosome X), one event involving amplification of an entire chromosomal arm (chromosome Xp), and 197 subchromosomal alterations including 151 deletions, 43 amplifications, one loss of heterozygosity, and two allelic imbalances (likely caused by subclonal populations) (Table S3A). Size ranges for subchromosomal alterations ranged from 0.1 Mb to 48.1 Mb (average 1.3 Mb; median 285 kb). For each of the 121 iPSC lines containing one or more CNV, we calculated the cumulative amount (in bp) of CNVs (average 3.8 Mb; median 473 kb) (Figure 3B). A small number of lines carried a disproportionate burden, with 19 lines having more than 2 Mb of CNVs and 33 having more than 1 Mb. Of note, these subchromosomal alterations are almost exclusively outside the detection limits of G-banded karyotyping, which typically cannot detect genomic abnormalities <5 Mb (Manning and Hudgins, 2007, Manning et al., 2010), and therefore these lines would be considered “normal” using a standard method of iPSC characterization. Thus, a majority (∼90%) of iPSCORE lines showed no detectable CNVs (101/222, or 45%) or carried CNVs less than 2 Mb (102/222, or 46%).

To investigate whether the somatic CNVs occurred prior to or during/following initial reprogramming (we cannot distinguish between mutations that occurred before or after the cell became an iPSC colony) versus during subsequent iPSC passaging in culture, we selected 17 iPSC lines containing a total of 33 CNVs at P12–P15, and compared their genotypes with a sample of the same line taken at an earlier passage (P3). Only three of the CNVs (9%) were not present at the earlier P3 version of the iPSC line, while 30 (91%) were present (Table S3B). For six of the iPSC lines (containing a total of 11 CNVs), we examined two additional clones at P3, and for one of the lines (containing two CNVs), we examined one additional clone at P3 (Table S3B). Only one of the 13 CNVs examined was present in another clone derived from the same fibroblast culture. These results are in agreement with previous studies that have found most somatic variants (single-nucleotide variants [SNVs] and CNVs) are present at low frequency in the cells of origin and are already present in early passages (Abyzov et al., 2012, Gore et al., 2011, Hussein et al., 2011, Laurent et al., 2011, Mayshar et al., 2010, Ruiz et al., 2013, Young et al., 2012). Our data suggest that systematically generated iPSC lines do not tend to acquire passage-associated CNVs (i.e., when passaged 12–15 times) and that most CNVs are detectable at early stages following iPSC derivation.

### Recurrently Altered Chromosomal Regions

To identify genomic regions that may be recurrently altered in iPSCs, we plotted the distribution of the 199 CNVs and then looked for 100-kb-long intervals containing more CNVs than expected by chance (Figure 3C). We observed five small regions (ranging from 200 to 400 kb) affecting 21 of 222 (9%) iPSC lines where CNVs occurred significantly more often than expected considering a uniform distribution across each individual chromosome (significance testing for aberrant copy number, p < 0.05) (Figures 3D–3H; Tables S3A and S3C). The most prevalent recurrent regions occurred on chr2 (chr2q23.3) and chr20 (chr20p12.1), containing an accumulation of five and seven subchromosomal CNVs (all deletions), respectively. The region on chr2 (chr2q23.3) lies in a relatively quiescent interval (not bound by regulatory proteins or modified histones) between two genes: *RPRM* (Reprimo, TP53 Dependent G2 Arrest Mediator Candidate), a tumor-suppressor gene involved in the regulation of p53-dependent cell-cycle arrest (Xu et al., 2012), and thus of potential interest due to the established importance of the p53 pathway in reprogramming (Krizhanovsky and Lowe, 2009); and *GALNT13* (Polypeptide N-Acetylgalactosaminyltransferase 13), a gene expressed at low levels in iPSCs that is involved in the glycosylation of mucins (Hang and Bertozzi, 2005). The chr4 region (chr4q23) overlaps active enhancers and an expressed gene in iPSCs: *TSPAN5* (tetraspanin 5), a member of the transmembrane 4 superfamily involved in the regulation of cell development and growth (Zhou et al., 2014). Although the gene (*RBFOX1*: RNA Binding Protein, Fox-1 Homolog) in the significantly enriched region on chr16 (16p13.3) is not expressed in iPSCs, the interval has previously been shown to be recurrently aberrantly methylated in iPSC lines (Ruiz et al., 2012). The chr20 region (chr20p12.1) affects a relatively quiescent interval and a protein-coding gene expressed at low levels in iPSCs: *MACROD2* (MACRO Domain Containing 2), a gene involved in autism (Jones et al., 2014) and in tamoxifen resistance in breast cancer (Mohseni et al., 2014). The chr22 region (chr22q12.1) affects two protein-coding genes, as well as an antisense RNA, all transcribed in iPSCs: *PITPNB* (Phosphatidylinositol Transfer Protein, Beta), *TTC28* (tetratricopeptide repeat domain 28), and TTC28-AS1 (TTC28 Antisense RNA 1). Although not a statistically significant enrichment in our study due to the relatively high number of CNVs in the iPSC lines on chr20 (resulting in a high background rate for this chromosome), we observed three CNVs overlapping the previously identified chr20q11.2 hotspot region (Laurent et al., 2011) linked with the pluripotency and cell proliferation-associated gene *DNMT3B* (DNA (Cytosine-5-)-Methyltransferase 3 Beta) (Lefort et al., 2008). The fact that the regions significantly enriched for CNVs in our study show other recurrent alterations (aberrant methylation on chr16) or contain actively transcribed genes involved in cell growth and development (chr4, chr22), suggest that these genomic intervals may have functional effects in iPSCs.

### iPSC-Derived Cardiomyocytes Can Be Used to Study Molecular and Physiological Traits

**Figure 4:**
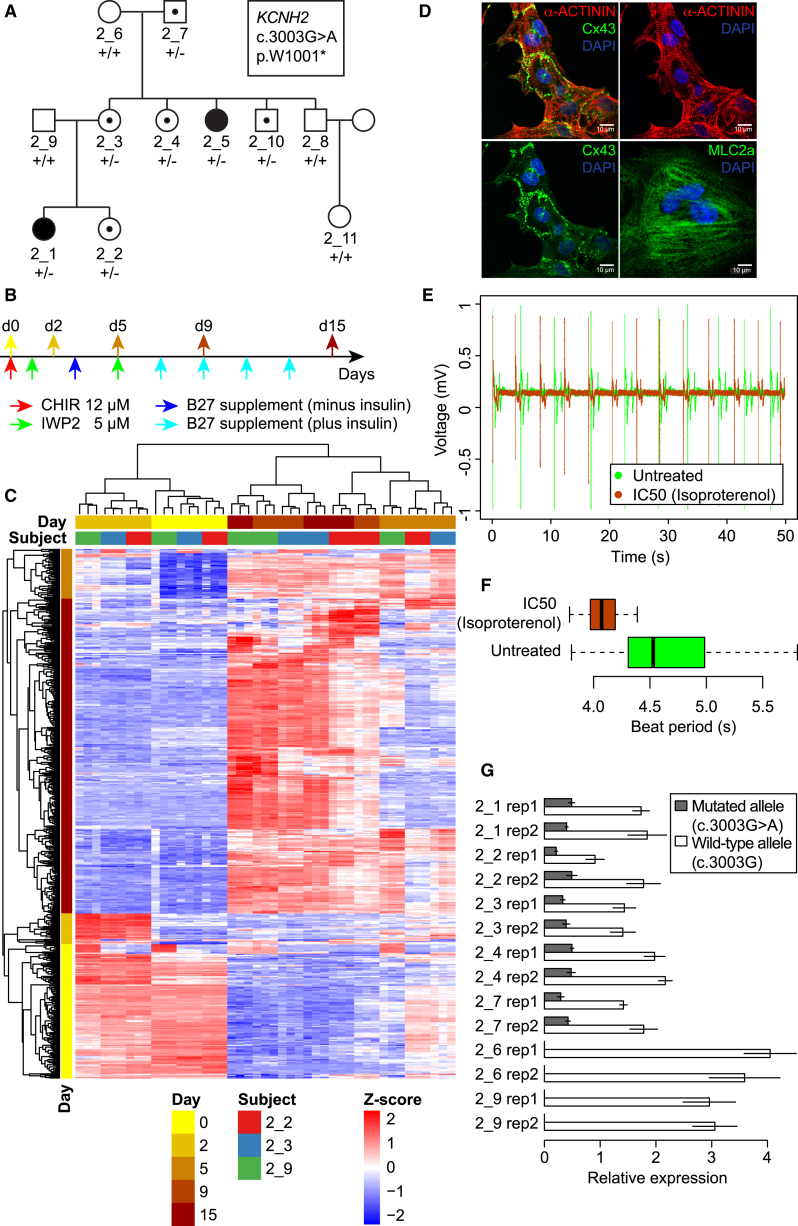
Differentiation of iPSC Lines into Cardiomyocytes and Functional Characterization (A) Pedigree of the iPSCORE family 2 showing segregation of *KCNH2* mutation (p.W1001^∗^) underlying dominant long-QT syndrome with incomplete penetrance. Individuals with filled in circles display long-QT syndrome, while individuals with black dots are carriers of the mutation. (B) Protocol used for cardiomyocyte differentiation (Lian et al., 2013). Arrows at the bottom indicate the reagents that were sequentially added to cell culture. Arrows at the top indicate the time points at which cells were collected for whole transcriptome analysis, corresponding to the differentiation stages of pluripotency (day 0 [d0]), mesodermal progenitors (d2), cardiovascular progenitors (d5), committed cardiovascular cells (d9), and cardiomyocytes (d15) (Paige et al., 2012). (C) Heatmap and hierarchical clustering of expression of the 500 genes with highest variance in expression levels among the 45 time-course samples. Samples (columns) are color coded based on the time point at which they were collected (days 0, 2, 5, 9, and 15) and on the subject from whom they were derived (2_2, 2_3, and 2_9). Genes (rows) are color coded by the four groups (hierarchical clustering), according to the differentiation stage where they were first expressed or most highly expressed (Table S4). Gene expression values are reported *Z* scores of variance stabilized transformed read counts. (D–F) Analysis of iPSC-derived cardiomyocytes from individual 2_3. (D) Confocal images of iPSC-CMs from sample 2_3 immunostained with sarcomeric α-actinin (ACTN1) (red), Cx43 (green), or MLC2-a (green) at day 34 post differentiation. Cx43 puncta are observed on hiPSC-CM cell membranes especially at cardiomyocyte cell-cell junctions. DAPI was used to counterstain nuclei. MEA analysis: (E) field potential measured from one electrode of one well before and after treatment of iPSC-CMs from sample 2_3 with isoproterenol (IC_50_ 0.01 μM), and (F) boxplot of beat period calculated from the same data. (G) Real-time qPCR specifically quantifying the transcripts of *KCNH2* with the two genotypes (mutated or wild-type), relative to GAPDH expression (ΔCt) in the iPSC-CMs from seven family members. Expression values are normalized relative to the average of ΔCt. Error bars represent SDs.

To demonstrate the utility of the iPSC lines for studying how genetic variants influence molecular and physiological traits in derived cells, we generated iPSC-derived cardiomyocytes (iPSC-CMs) from individuals in a three-generational family that shows segregation of long-QT syndrome type II (Figure 4A). We differentiated three individuals (2_2, 2_3, and 2_9) in triplicate and profiled them using RNA-seq at five different cardiac differentiation stages (45 independent samples) (Figure 4B). The five profiled stages were each subsequent to important chemical stimuli in the differentiation process that were previously shown to result in epigenetic changes (Paige et al., 2012): pluripotent (day 0 [d0]), mesodermal progenitors (d2), cardiovascular progenitors (d5), committed cardiovascular cells (d9), and cardiomyocytes (d15). We selected the 500 most variably expressed autosomal genes, divided them into four groups using hierarchical clustering, and annotated them according to the differentiation stage where they were most highly expressed (89 genes expressed at d0, 26 at d2, 41 at d5, and 274 at the combined d9–d15) (Figure 4C). The triplicate samples for each individual at each of the five stages clustered together (the two sets of triplicates at d9 and d15 clustered), suggesting that genetic background correlates with expression differences between the different iPSC lines and derived cardiomyocytes. We performed functional enrichment analysis to confirm that genes in each of the four groups of genes recapitulate important stages of cardiac development. This analysis showed that the genes in group d0 were enriched in gene ontology terms associated with stem cells and processes involved in the specification of cell identity, group d2 genes were involved in mesoderm development and gastrulation, and group d5 genes were associated with embryo and organ development, whereas genes in group d9–d15 were involved in heart muscle development (Table S4). These results are in accordance with the cardiac differentiation stages described by Paige et al. (2012). The iPSC-CMs from sample 2_3 were further interrogated by immunofluorescence for the presence of typical cardiac structural markers, ACTN1, CX43, and MLC2a, and this confirmed that cardiomyocyte-like sarcomeres and gap junctions had developed (Figure 4D). Thus, the iPSCs can be differentiated to cardiomyocytes that show appropriate cardiac morphological structures as well as gene expression patterns that cluster by genetic background.

iPSC-CMs could potentially be used as a model system to assess individual response to drugs through in vitro functional and pharmacological assays. We characterized cardiomyocytes from 2_3 and three additional cell lines (2_1, 13_1, and 14_2) using multielectrode array analysis (MEA), which records extracellular field potentials of clusters or layers of cells and provides measurement of cardiac electrophysiology analogous to an electrocardiogram recording (Figures 4E and S4). All four cell lines displayed cardiomyocyte-like electrophysiological proprieties. When we exposed sample 2_3 to isoproterenol, a β_1_ and β_2_ adrenoreceptor agonist used for the treatment of bradycardia and heart block, cardiomyocytes showed a significantly increased beat rate (Figures 4E and 4F), consistent with previous reports (Mandel et al., 2012, Scott et al., 2014, Sirenko et al., 2013). Thus, these observations suggest that cardiomyocytes derived from this collection show expected electrophysiological properties in response to drug stimulus, and therefore may allow for studying the genetic components underlying drug response differences between individuals.

The long-QT syndrome that shows segregation in iPSCORE family 2 (Figure 4A; Table S1A) is caused by the p.W1001^∗^ mutation in *KCNH2*, which encodes the α subunit of a potassium ion channel essential for the final repolarization of the ventricular action potential (Kupershmidt et al., 2002). It has been proposed that the disease mechanism for this mutation is the reduction of the rapid delayed rectifier current (IKr) due to degradation of the transcript by nonsense-mediated mRNA decay (NMD), and consequent prolongation of the action potential (Gong et al., 2007). To examine this hypothesis, we generated d15 iPSC-derived cardiomyocytes from additional family members (2_1, 2_4, 2_6, and 2_7) and analyzed *KCNH2* expression in all seven individuals by allele-specific qPCR. We found that in the carriers of the mutation, the transcript from the mutated allele was reduced by ∼75% (t test p = 7.4 × 10^−7^) with respect to the wild-type allele, consistent with the proposed NMD hypothesis (Figure 4G). These results demonstrate that iPSC-derived cell types can be used to investigate mechanisms underlying the association of genetic variation with molecular, physiological, and disease phenotypes.

### iPSC Lines Carry Genetic Variants Associated with a Variety of Traits and Diseases

**Figure 5:**
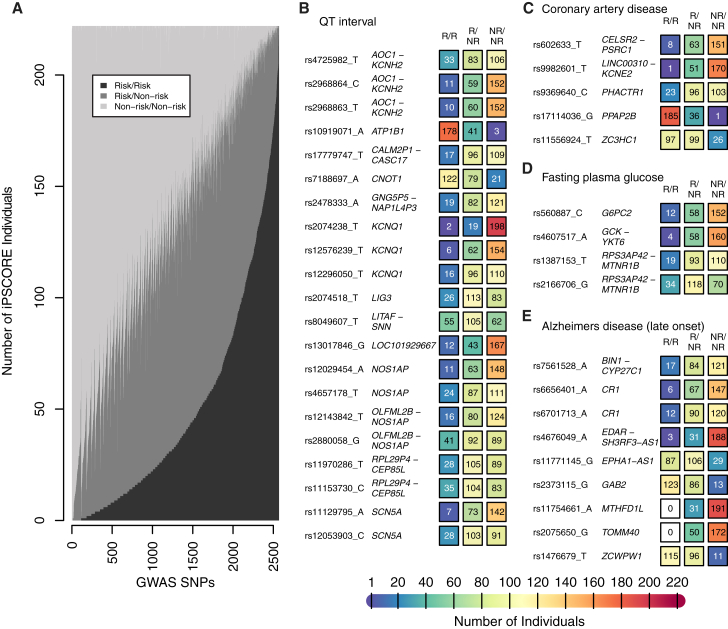
Distributions of GWAS SNP Genotypes in the iPSCORE Resource (A) Stacked barplot showing the number of individuals in the iPSCORE resource that have particular genotypes at 2,571 SNPs that have been previously associated with one or more phenotypes through GWAS. (B–E) Counts of individuals that carry the risk/risk (R/R), risk/non-risk (R/NR), and non-risk/non-risk (NR/NR) genotypes for SNPs implicated in the indicated disease. The color of the box indicates the number of individuals on a color scale shown at the bottom. (B) QT interval; (C) coronary artery disease; (D) fasting plasma glucose levels; and (E) Alzheimer's disease (late onset). See also Table S5.

Given that many of the iPSC lines in the iPSCORE resource are from healthy donors, they may be useful for examining common genetic variants associated with non-cardiac phenotypes. GWAS have examined hundreds of human phenotypes and identified thousands of SNPs associated with one or more trait (Cingolani et al., 2012). We identified 2,571 of these GWAS SNPs present on the HumanCoreExome arrays that are associated with one or more phenotype and report the risk allele genotypes from the germline samples of the 222 participants (Figure 1A and Table S5). In addition, we examined the distribution of risk/risk, risk/non-risk, and non-risk/non-risk genotypes at these GWAS SNPs and found that for 95% (2,434/2,571), each of the three genotypes was represented, the totals for which can be seen in Figure 5A. These phenotypes include those that are relevant to cardiovascular disease, diabetes, and neurological health, such as QT interval, coronary artery disease, fasting glucose levels, and late-onset Alzheimer's disease (Figures 5B–5E). It has been shown that iPSC lines can be differentiated into a variety of human cell types, including adipocytes (Lian et al., 2016), cardiomyocytes (Burridge et al., 2014), hematopoietic progenitor cells (Ferrell et al., 2015), pancreatic β cells (Tulpule et al., 2013), and several different neuronal cell types (Sances et al., 2016). Thus, the iPSC lines in the iPSCORE resource could be used to investigate the molecular mechanisms underlying the genetic risk for a wide variety of traits and diseases in the appropriate derived cell types.

## Discussion

Current large-scale collections of iPSCs generally have limited numbers of lines from people of non-European ancestry or individuals in multigenerational families. The iPSCORE collection includes 75 lines from people of Hispanic ethnicity, non-European ancestry, or multiple ancestries, which will aid in studies interrogating population-associated genetic variation or in fine-mapping using trans-ethnicity mapping. Additionally we include multigenerational families and monozygotic twins, which will enable interrogation of rare, family-specific variation, segregation analysis of molecular and physiological traits, and estimation of technical and environmental variation independent of genetic background. The 136 genetically unrelated individuals in the resource enable the derived cell lines to be used for genetic association studies that historically have required unrelated individuals; although with methods that account for sample structure (Kang et al., 2010), these studies can incorporate all 222 individuals. These association studies will be further augmented by the fact that whole-genome sequence data has been generated from somatic tissue (blood and in some cases fibroblasts) of the iPSCORE participants and is part of the resource (Table S1C). Because risk and non-risk alleles for the vast majority of GWAS SNPs are represented in the genomes of the 222 individuals, this resource will allow for the functional interrogation of these important predominantly regulatory variants in appropriate iPSC-derived cell types. Thus, the nature of the individuals who participated in the iPSCORE resource will allow for diverse experimental approaches to examine how genetic variation affects molecular and physiological traits.

To efficiently characterize more than 200 iPSC lines, we incorporated genomic tools such as the HumanCoreExome BeadChip to examine genomic integrity, establish sample identity, and estimate genetic ancestry and familial relatedness; and RNA-seq to establish pluripotency. Overall, genomic integrity for these low-passage lines was high with almost half of the iPSCs in the iPSCORE resource showing no detectable abnormalities, and ∼90% showing less than 2 Mb of cumulative CNV coverage (in bp). It is important to note that genotype array assays are limited to the extent that they are unable to detect balanced chromosomal translocations or abnormalities occurring at a frequency lower than 20% (D'Antonio et al., 2017 [this issue of *Stem Cell Reports*]); however, previous studies using genotype arrays have found higher ratios and frequencies of abnormalities in iPSCs (International Stem Cell et al., 2011, Laurent et al., 2011, Taapken et al., 2011) than we report, suggesting that a systematic approach to iPSC generation can result in significantly fewer abnormalities. We also used RNA-seq data to validate the quality of the iPSCORE lines by comparing them with publicly available RNA-seq data for stem cells previously shown to be pluripotent and performed pluripotency estimation using PluriTest-RNAseq. Thus, the adoption of high-throughput genomic methods can help reduce costs and enable effective and relatively rapid characterization of iPSC lines for genomic integrity and pluripotency.

Although we observed low overall rates of CNVs, we observed five recurrently altered regions. Three of the intervals are quiescent, containing few (if any) regulatory elements and either low or unexpressed genes. However, one of these intervals (the chr16 interval) is recurrently aberrantly methylated in iPSC lines (Ruiz et al., 2012), which suggests that the region has functional significance in iPSCs. The other two recurrently altered intervals contain actively transcribed genes involved in cell growth and development in iPSCs. Further studies are needed to determine whether these significantly altered intervals offered a selective advantage in the reprogramming process or were due to hotspots that recurrently mutate at a low rate (2%–4%) in iPSCs (or the parental cells). Previous studies have shown that most somatic variants (both CNVs and SNVs) observed in iPSCs are already present in the cell of origin (Abyzov et al., 2012, Cheng et al., 2012, Gore et al., 2011, Ruiz et al., 2013, Young et al., 2012). We observed in a small number of lines that the majority of somatic CNVs observed in later-passage iPSCs (P12) were already present at earlier passages (P3), supporting the model that most somatic variants are likely derived from the parental cell. In total, these data suggest that while a significant number of our systematically generated iPSCs examined at relatively early passage (P12) do not harbor detectable genomic alterations, some iPSCs showed recurrently altered genomic intervals that may reveal a selective advantage during the reprogramming process, and that many of these may be present in the cell of origin.

In summary, iPSCORE is a high-quality large-scale collection of iPSCs from 222 individuals that is currently publicly available through the NHLBI-contracted biorepository at WiCell Research Institute, with phenotype and genomic data (SNP arrays, RNA-seq, whole-genome sequencing) being released through public databases. We are currently using the resource to differentiate the iPSC lines into cardiomyocytes with the intention of investigating molecular (ATAC-seq [assay for transposase-accessible chromatin with high-throughput sequencing], DNA methylation, H3K27ac marks) and physiological phenotypes in both iPSC lines and iPSC-derived cardiomyocytes. As these, and other genomic (such as whole-genome sequences) and molecular data for a variety of derived cell types become available, the resource will become substantially richer over time, enabling the research community to efficiently address a multitude of questions regarding human biology and disease.

## Experimental Procedures

### Enrollment of Subjects for the iPSCORE Resource

This resource was established as part of the Next Generation Consortium of the National Heart, Lung and Blood Institute and is available to researchers through the biorepository at WiCell Research Institute (www.wicell.org; NHLBI Next Gen Collection). For-profit organizations can contact the corresponding author directly to discuss line availability. Healthy individuals were recruited for the resource through both the Twin Sibling Pedigree cohort (TSP; a population-based twin registry spanning counties in Southern California) (Pasha et al., 2013) and open enrollment through the Clinical and Translational Research Institute (CTRI) at the University of California at San Diego (UCSD). Thirty-nine patients at UCSD Sulpizio Cardiovascular Center were also recruited. These collections were approved by the Institutional Review Boards of the UCSD and The Salk Institute (Project no. 110776ZF). Each of the subjects first consented to the study and filled out a questionnaire. These data were transcribed to a database and subjects were de-identified with a new sample ID (Table S1A). Family relatedness was also recorded in the questionnaire and converted into pedigree diagrams using family tree drawing software (Madeline 2.0, University of Michigan) (Figure S1). Ethnicity was reported as a free-response answer and translated into one of six recorded ethnicity groupings (African American, European, Hispanic, Indian, Middle Eastern, Asian) (Table S1A). A seventh category was used when more than one ethnicity was reported; that individual was recorded as “Multiple ethnicities reported.” For 12 individuals, the race/ethnicity question was reported by a treating physician (Table S1A, denoted by an asterisk in column K). Finally, a blood sample (for germline DNA) was collected and a skin biopsy was performed to generate fibroblast stocks for iPSC reprogramming.

## Author Contributions

K.A.F., O.H., and A.D.P. conceived the study. A.D.A., M.G., J.D.G., F.D., K.E.D., Y.H., V.M., A.D.P., J.O., C.T.D., R.F., P.B., K.F., M.C., V.B., C.A.M., and A.D.-C. generated fibroblasts, iPSCs, and iPSC-derived cardiomyocytes. C.Z. performed flow cytometry. S.I.H. and A.A. performed imaging. P.B. performed qRT-PCR. M.D., P.B., C.D., H.M., J.R., R.W., D.A.J., M.K.R.D., W.W.G., H.L., N.N., B.M.S., F.-J.M., and E.N.S. performed data processing and computational analyses. K.J., B.C.N., T.J.M., F.R., D.T.O., E.A., and N.C.C. participated in recruitment and enrollment of individuals into study. K.A.F., J.C.I.B., N.C.C., G.W.Y., S.M.E., L.S.B.G., W.T.B., J.F.L., E.N.S., and A.D.P. oversaw and managed the study. K.A.F., M.D., P.B., E.N.S., and A.D.P. prepared the manuscript.

## Accession Numbers

Phenotype, array genotype, RNA-seq expression values, and whole-genome sequence genotype data are available through dbGaP: phs000924 and phs001325. The 222 iPSC lines are available through WiCell Research Institute (www.wicell.org; NHLBI Next Gen Collection). Note: The informed consent for the individuals in the iPSCORE resource included the allowance for commercial use. However, licensing agreements are still required between individual commercial entities and DNAVEC (maker of SeV) and Wicell (iPSC bank).

## Supplemental Information

Document S1. Supplemental Experimental Procedures and Figures S1–S5

Table S1. Phenotype Information for Participants in iPSCORE Collected at Enrollment and Identifiers for Cell Lines and Genomic Data, Related to Figure 1

Table S2. Pluripotency of 213 iPSC Lines Calculated by PluriTest RNA-Seq, Related to Figure 2

Table S3. CNV Analysis of the 222 iPSC Lines, Related to Figure 3

Table S4. Gene Ontology Terms Associated with Four Gene Cluster Groups in Time-Course Study, Related to Figure 4C

Table S5. Genotype Data from 222 Germline Samples at 2,571 GWAS SNPs Measured by the HumanCoreExome BeadChip, Related to Figure 5

Document S2. Article plus Supplemental Information
