# Supplementary material for: iPSCORE: A Resource of 222 iPSC Lines Enabling Functional Characterization of Genetic Variation across a Variety of Cell Types
Source: Stem Cell Reports. 2017 Apr 6;8(4):1086–100. doi: 10.1016/j.stemcr.2017.03.012 (PMC5390244; doi:10.1016/j.stemcr.2017.03.012)
Supplement: Document S1. Supplemental Experimental Procedures and Figures S1–S5 [file mmc1.pdf]

Stem Cell Reports, Volume *8*

Supplemental Information

iPSCORE: A Resource of 222 iPSC Lines Enabling Functional Characterization of Genetic Variation across a Variety of Cell Types

Athanasia D. Panopoulos, Matteo D'Antonio, Paola Benaglio, Roy Williams, Sherin I. Hashem, Bernhard M. Schuldt, Christopher DeBoever, Angelo D. Arias, Melvin Garcia, Bradley C. Nelson, Olivier Harismendy, David A. Jakubosky, Margaret K.R. Donovan, William W. Greenwald, KathyJean Farnam, Megan Cook, Victor Borja, Carl A. Miller, Jonathan D. Grinstein, Frauke Drees, Jonathan Okubo, Kenneth E. Diffenderfer, Yuriko Hishida, Veronica Modesto, Carl T. Dargitz, Rachel Feiring, Chang Zhao, Aitor Aguirre, Thomas J. McGarry, Hiroko Matsui, He Li, Joaquin Reyna, Fangwen Rao, Daniel T. O'Connor, Gene W. Yeo, Sylvia M. Evans, Neil C. Chi, Kristen Jepsen, Naoki Nariai, Franz-Josef Müller, Lawrence S.B. Goldstein, Juan Carlos Izpisua Belmonte, Eric Adler, Jeanne F. Loring, W. Travis Berggren, Agnieszka D'Antonio-Chronowska, Erin N. Smith, and Kelly A. Frazer

Supplemental Information includes Figures S1-S5, Tables S1-S5 and Supplemental Experimental Procedures.

SUPPLEMENTARY FIGURES

Figure S1. Structures of the 41 families in the iPSCORE resource. Related to Figures 1A, 1C and 1D.

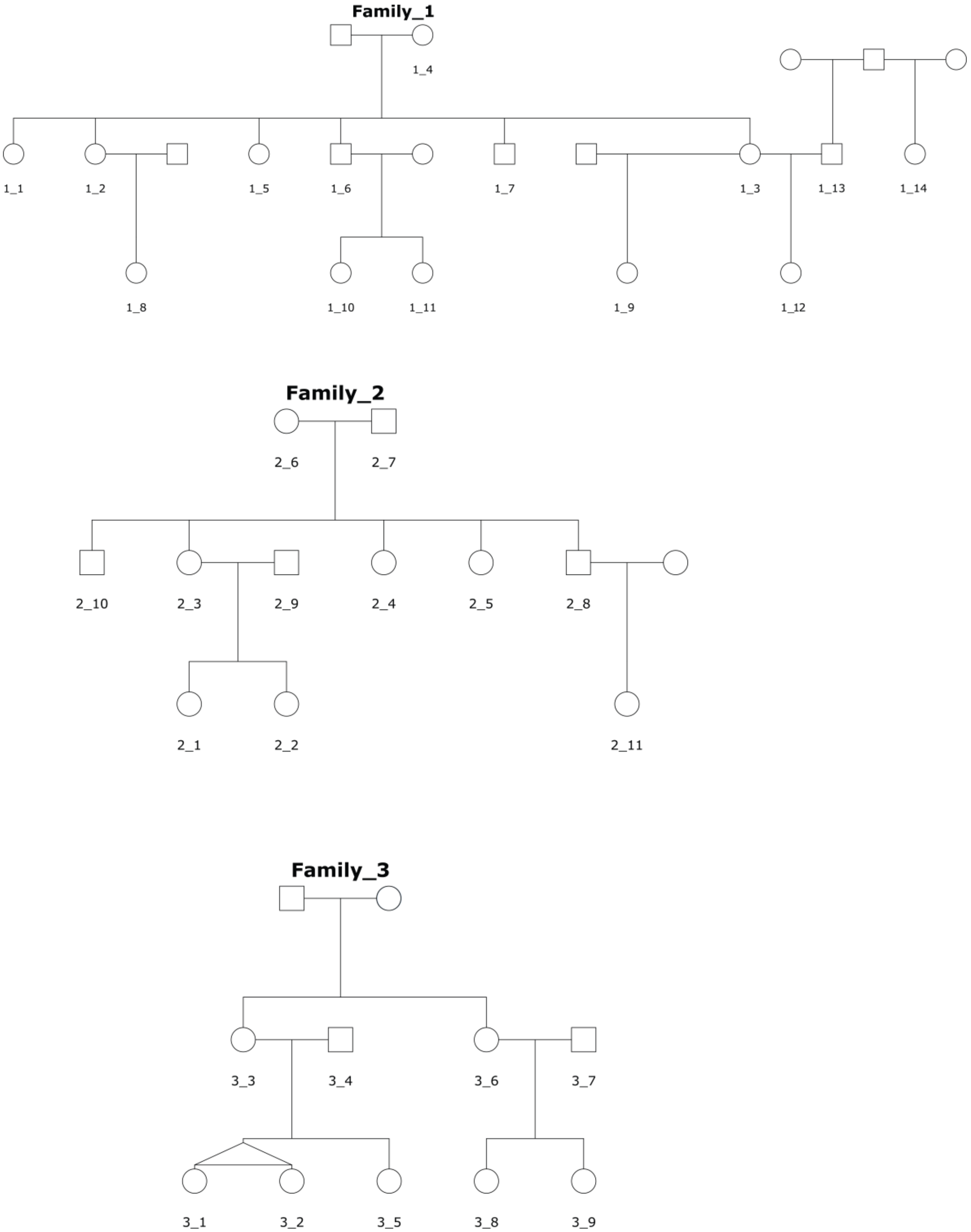

Figure S1 (continued)

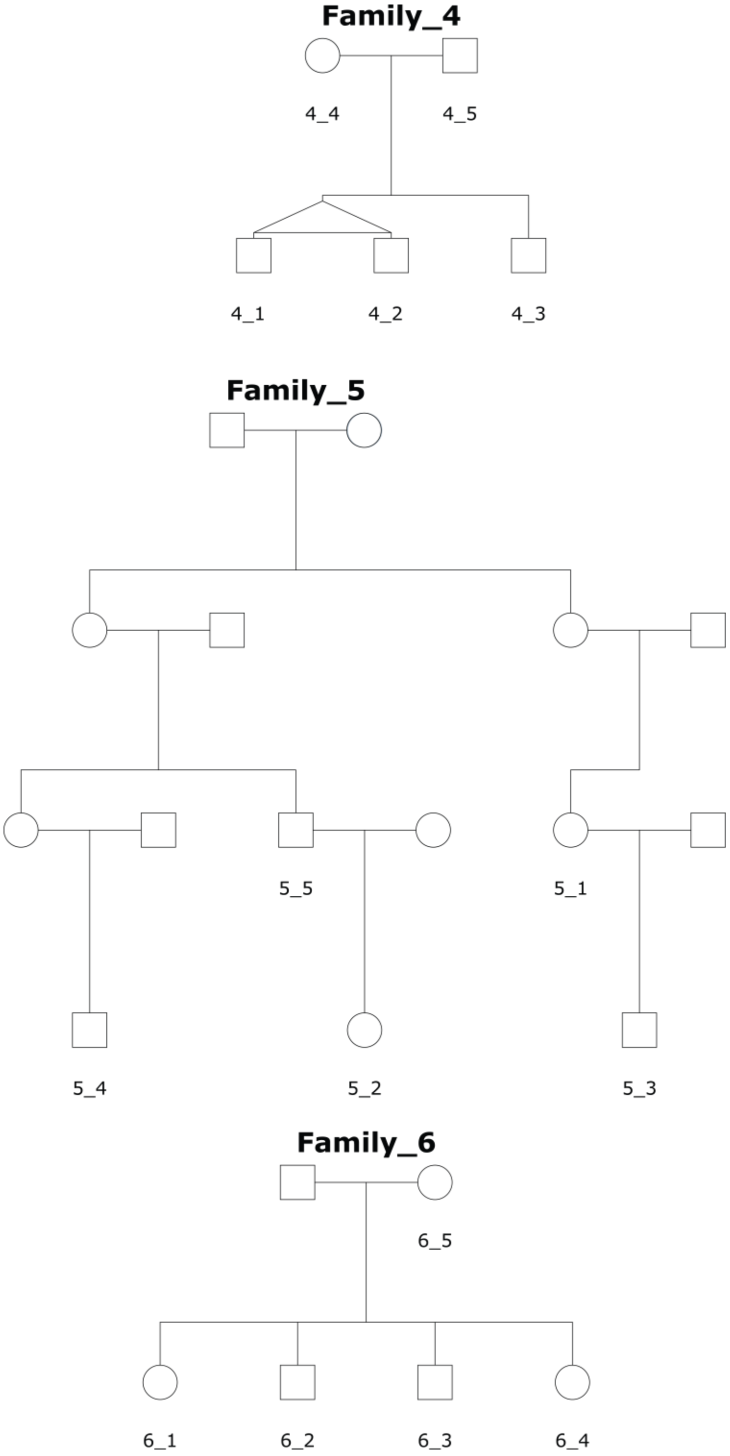

Figure S1 (continued)

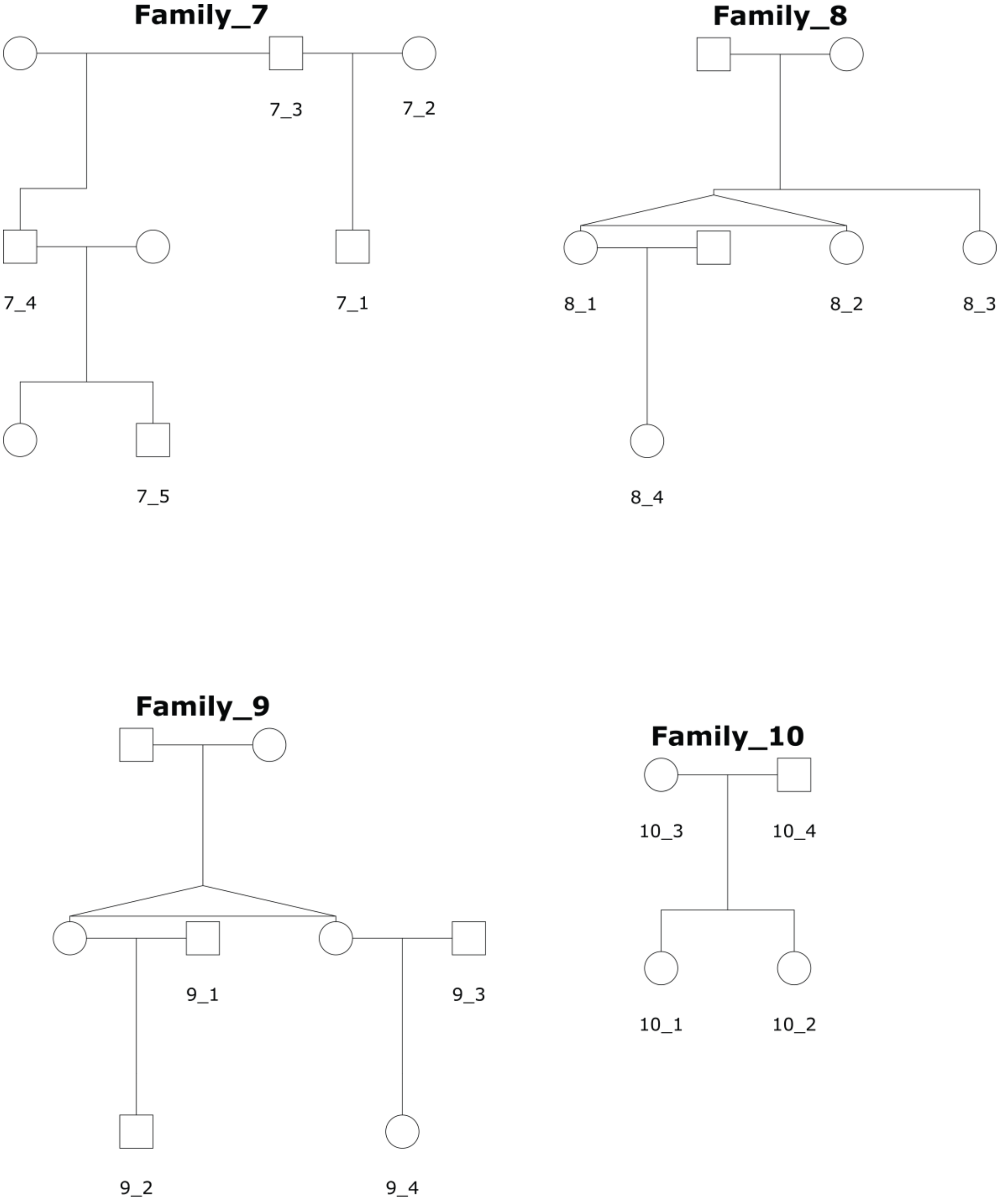

Figure S1 (continued)

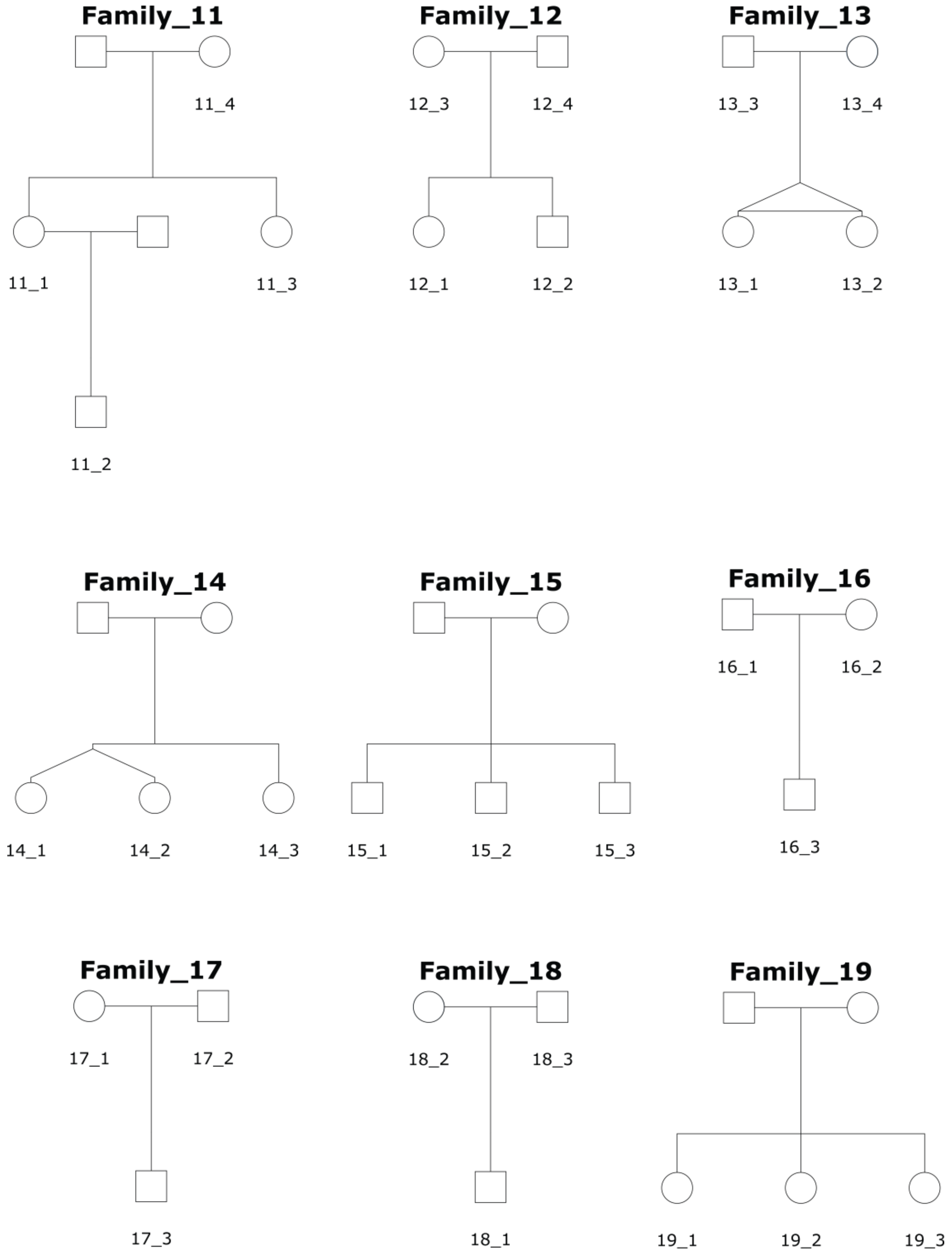

Figure S1 (continued)

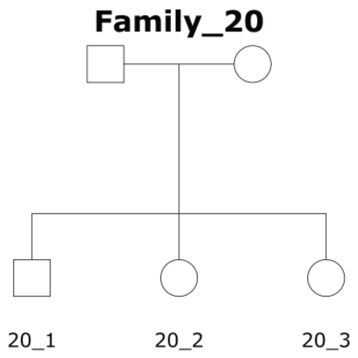

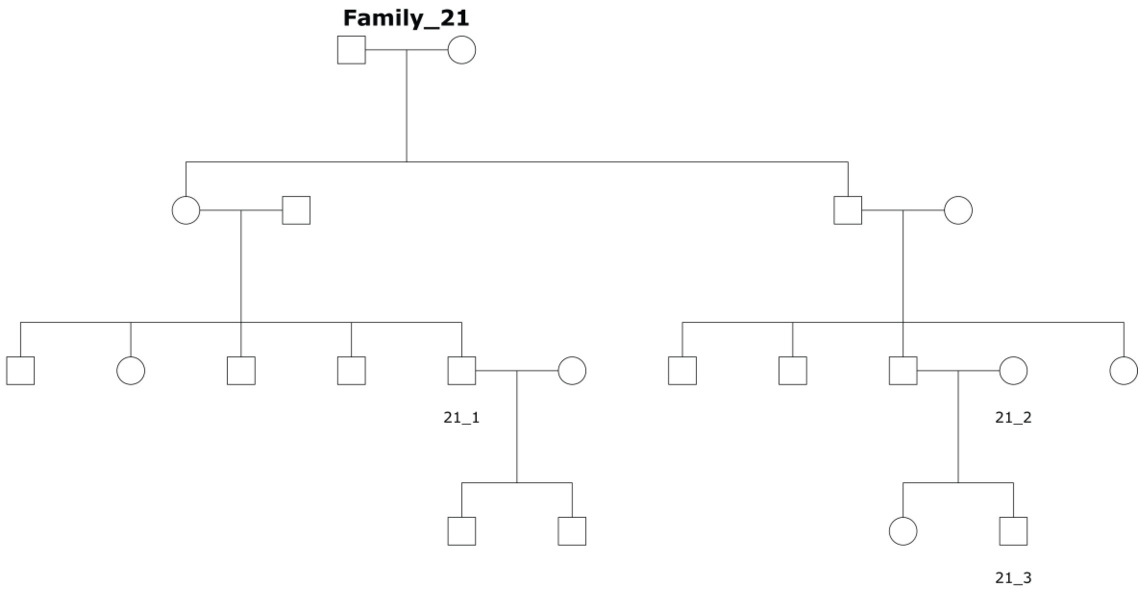

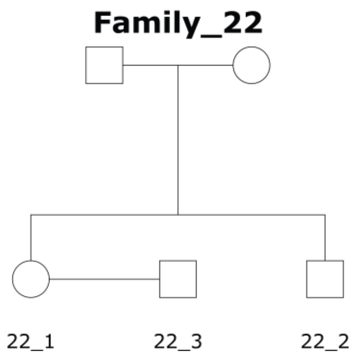

Figure S1 (continued)

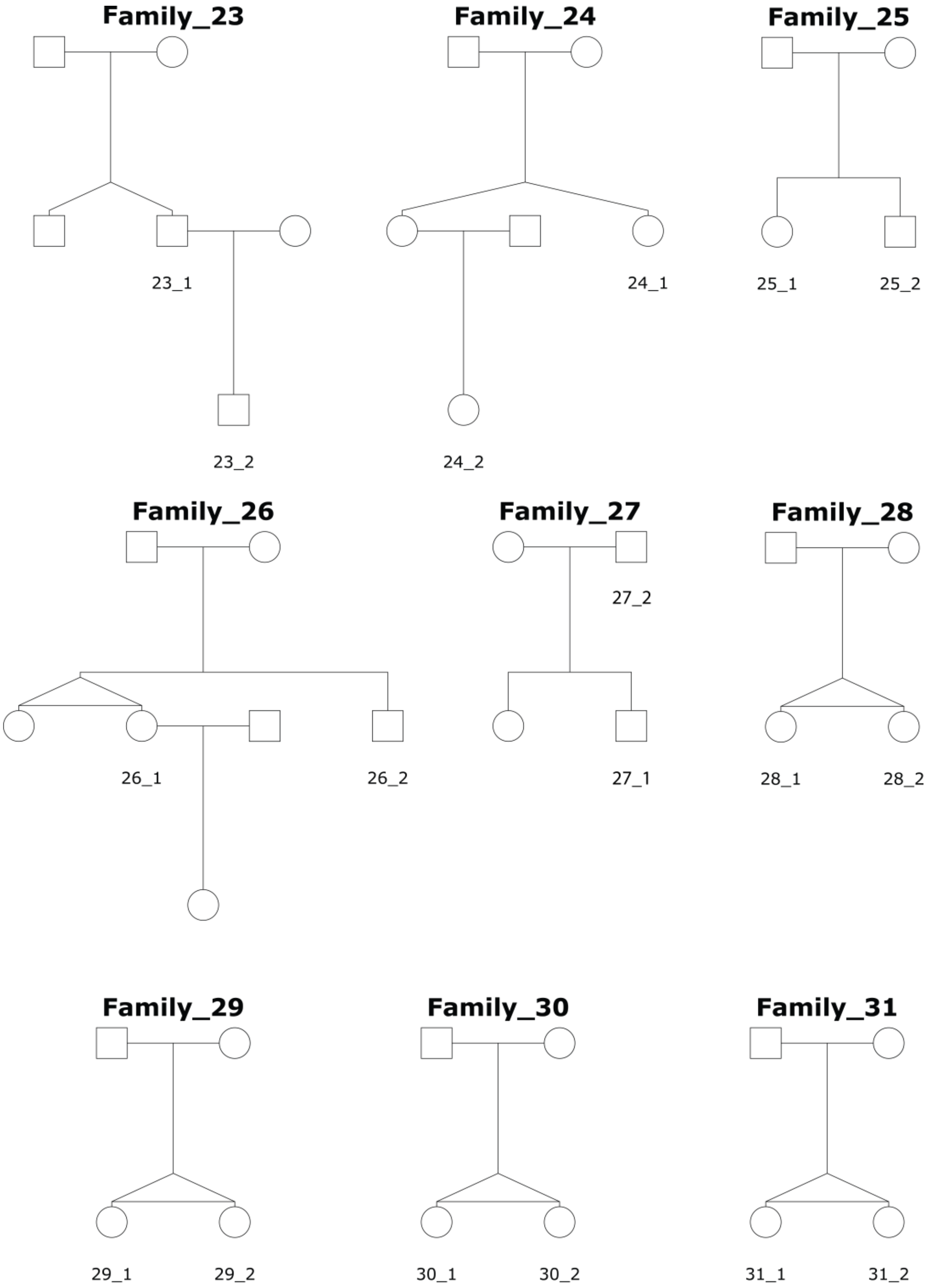

Figure S1 (continued)

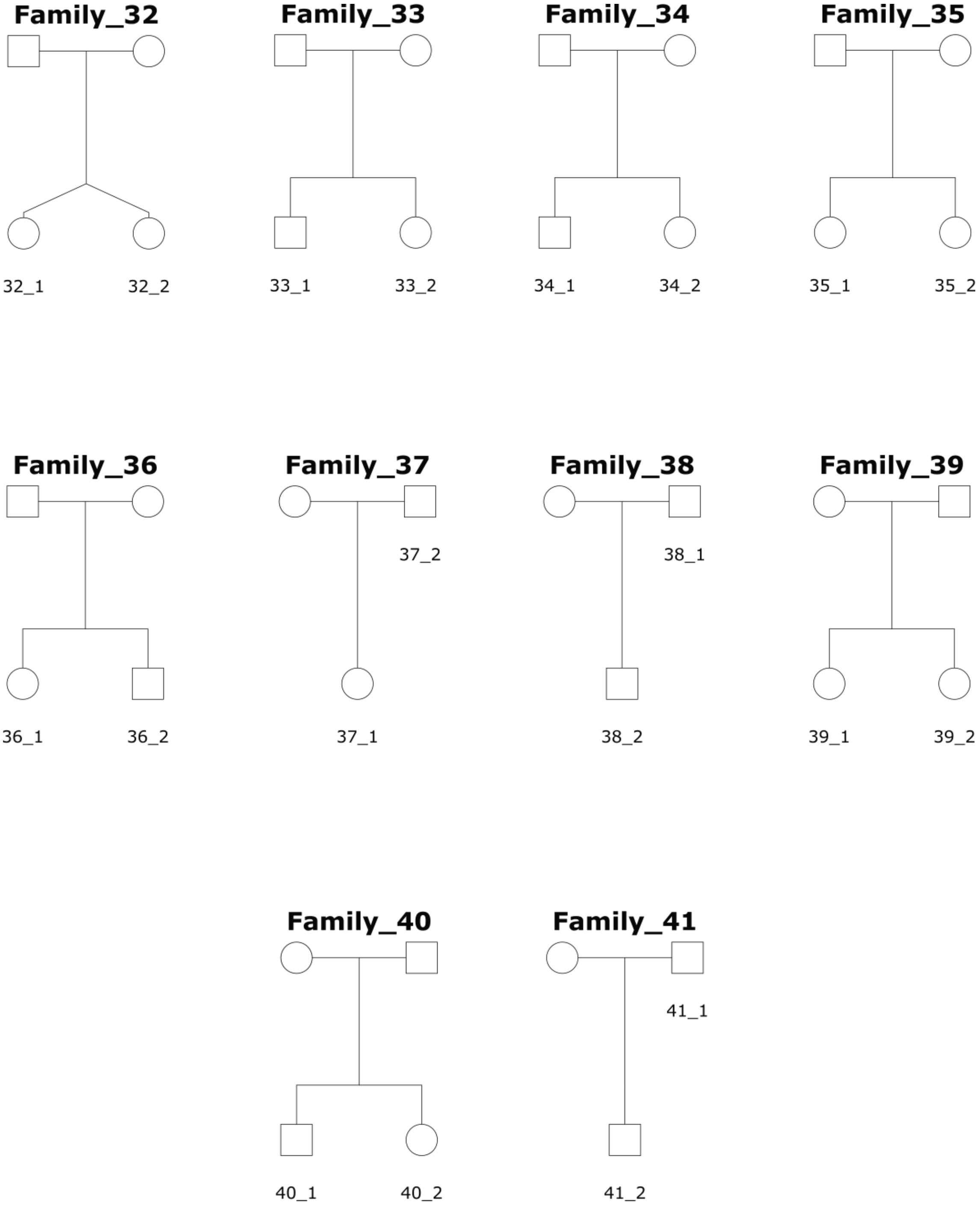

Family relatedness recorded through questionnaires was translated into pedigree diagrams for all subjects with at least one other family member in the cohort. The numbered individuals in each pedigree are the subjects for which iPSC lines were derived (see Table S1A for additional

phenotype data). Monozygotic twin pairs are drawn with a triangle, while dizygotic twin pairs are drawn with angled lines.

Figure S2. Analysis of pluripotent marker expression in iPSC lines by flow cytometry. Related to Figure 2.

A.

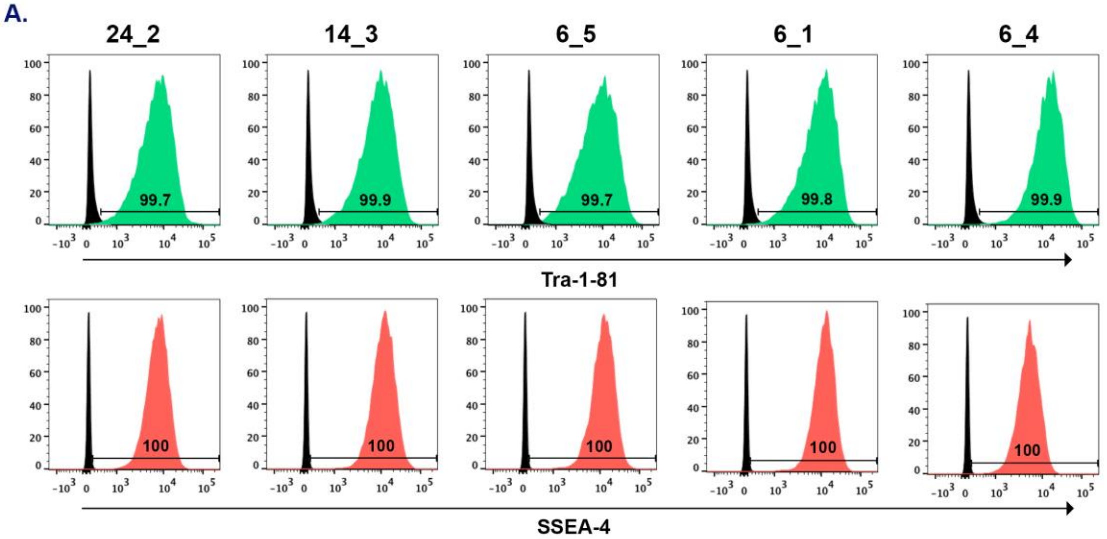

B.

Cell Line ID	% SSEA-4	%TRA-1-81
CARDiPS_24_2_iPSC_C4_P12	100.0	99.7
CARDiPS_14_3_iPSC_C2_P12	100.0	99.9
CARDiPS_6_5_iPSC_C1_P12	100.0	99.7
CARDiPS_6_1_iPSC_C2_P12	100.0	99.8
CARDiPS_6_4_iPSC_C3_P12	100.0	99.9
CARDiPS_14_1_iPSC_C3_P12	100.0	97.7
CARDiPS_6_2_iPSC_C5_P12	99.7	99.9
CARDiPS_2_11_iPSC_C2_P12	99.6	98.9
CARDiPS_7_5_iPSC_C9_P12	99.3	99.3
CARDiPS_15_3_iPSC_C3_P12	100.0	99.6
CARDiPS_15_1_iPSC_C6_P12	99.8	99.4
CARDiPS_7_4_iPSC_C6_P12	97.3	99.1
CARDiPS_6_3_iPSC_C6_P12	99.9	99.0
CARDiPS_17_1_iPSC_C5_P12	99.9	98.0
CARDiPS_15_2_iPSC_C2_P12	100.0	99.8
CARDiPS_11_4_iPSC_C4_P12	99.9	99.9
CARDiPS_14_2_iPSC_C5_P12	100.0	99.8
CARDiPS_12_3_iPSC_C3_P12	100.0	99.7
CARDiPS_4_3_iPSC_C1_P12	100.0	99.9
CARDiPS_2_1_iPSC_C4_P12	100.0	99.9
CARDiPS_2_3_iPSC_C5_P13	100.0	100.0
CARDiPS_7_3_iPSC_C3_P12	99.7	98.7
CARDiPS_11_3_iPSC_C5_P12	99.9	99.4
CARDiPS_4_4_iPSC_C4_P13	99.9	95.7
CARDiPS_12_1_iPSC_C2_P12	99.9	99.5

Cell Line ID	% SSEA-4	%TRA-1-81
CARDiPS_17_2_IPSC_C2_P12	100.0	99.6
CARDiPS_7_2_iPSC_C2_P12	99.8	99.8
CARDiPS_11_1_iPSC_C2_P12	100.0	99.9
CARDiPS_2_6_iPSC_C6_P12	100.0	95.0
CARDiPS_19_3_iPSC_C7_P13	98.4	99.0
CARDiPS_2_9_iPSC_C5_P12	100.0	96.6
CARDiPS_18_2_iPSC_C5_P12	100.0	95.2
CARDiPS_18_3_iPSC_C3_P12	100.0	99.3
CARDiPS_30_1_iPSC_C3_P13	100.0	99.9
CARDiPS_30_2_IPSC_C5_P13	99.9	100.0
CARDiPS_12_2_IPSC_C1_P12	100.0	98.7
CARDiPS_11_2_iPSC_C4_P12	100.0	99.8
CARDiPS_19_2_iPSC_C7_P14	99.9	97.2
CARDiPS_2_4_iPSC_C2_P13	98.3	97.3
CARDiPS_3_1_iPSC_C2_P19	99.8	99.4
CARDiPS_3_2_iPSC_C11_P19	100.0	98.5
CARDiPS_34_2_iPSC_C2_P13	99.7	95.5
CARDiPS_36_2_iPSC_C2_P13	99.9	99.3
CARDiPS_36_1_iPSC_C1_P13	99.9	95.9
CARDiPS_2_7_iPSC_C3_P13	100.0	99.5
CARDiPS_8_1_iPSC_C3_P13	99.9	95.6
CARDiPS_8_2_iPSC_C6_P13	100.0	99.8
CARDiPS_32_1_iPSC_C3_P14	99.7	98.1
CARDiPS_56_1_iPSC_C3_P13	99.5	95.0
CARDiPS_62_1_iPSC_C3_P13	99.3	95.0

Flow cytometry analysis of the cell surface pluripotency markers Tra-1-81 and SSEA-4 was performed in a subset of the iPSCORE iPSC lines (50 total). (A) An example of the type of analysis performed is shown for five of the iPSC lines. The individual from which the iPSC line is derived is indicated by the subject id shown at the top. (B) The percentages of cells that were positive for each individual marker are summarized, demonstrating that all iPSC lines had >95% positive expression for both pluripotent markers (Tra-1-81 and SSEA-4).

Figure S3. Distribution of CNVs across the genome. Related to Figure 3.

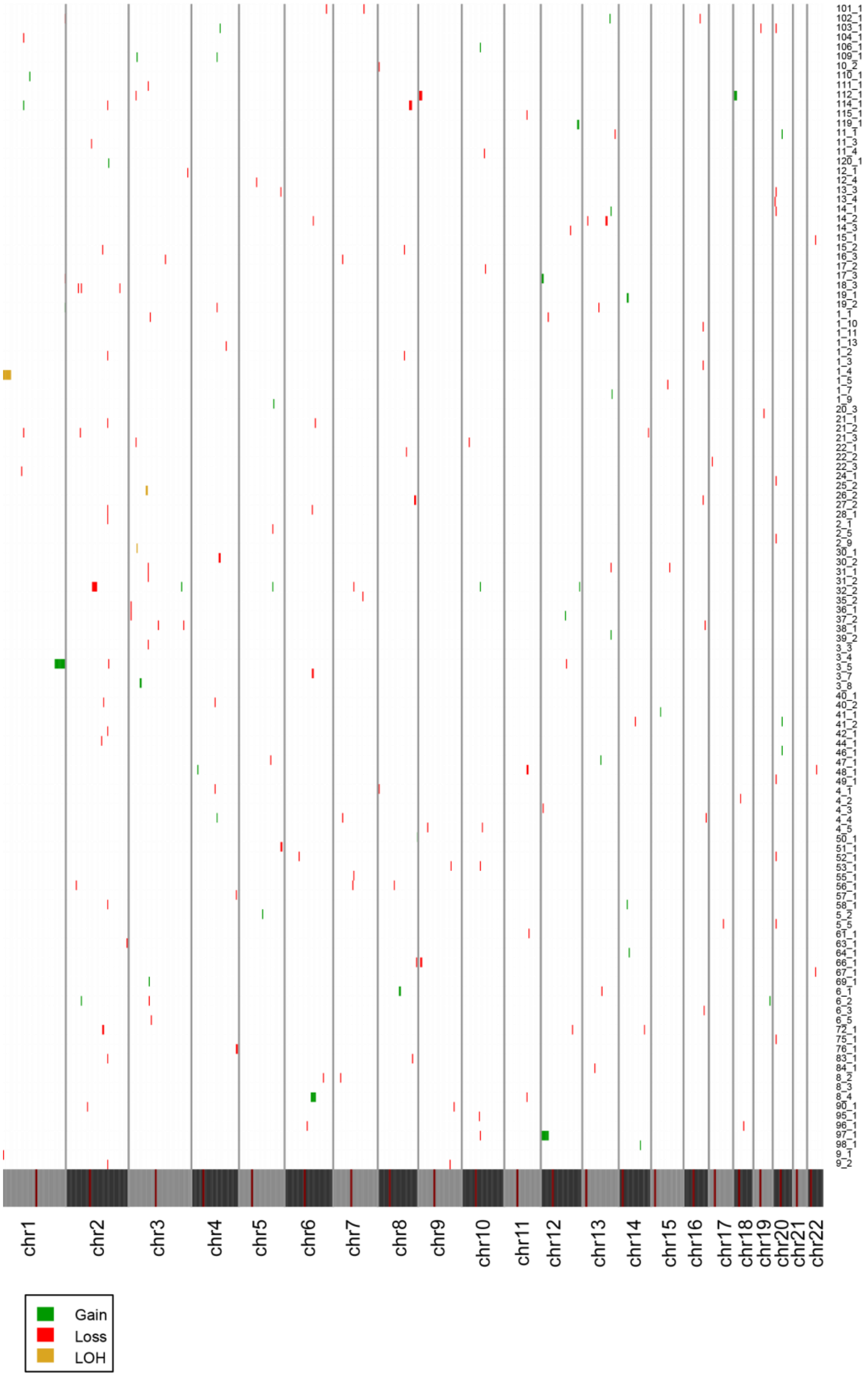

Heatmap showing genomic positions (columns) in the 121 iPSC lines that harbor at least one CNV (rows). Colors refer to different types of alterations, as indicated. The individual from which the iPSC line is derived is indicated by the subject id shown at the right. See Table S3A for coordinates of CNVs.

Figure S4. Multi-electrode array (MEA) analysis of cardiomyocytes (CM) differentiated from three iPSC lines. Related to Figure 4.

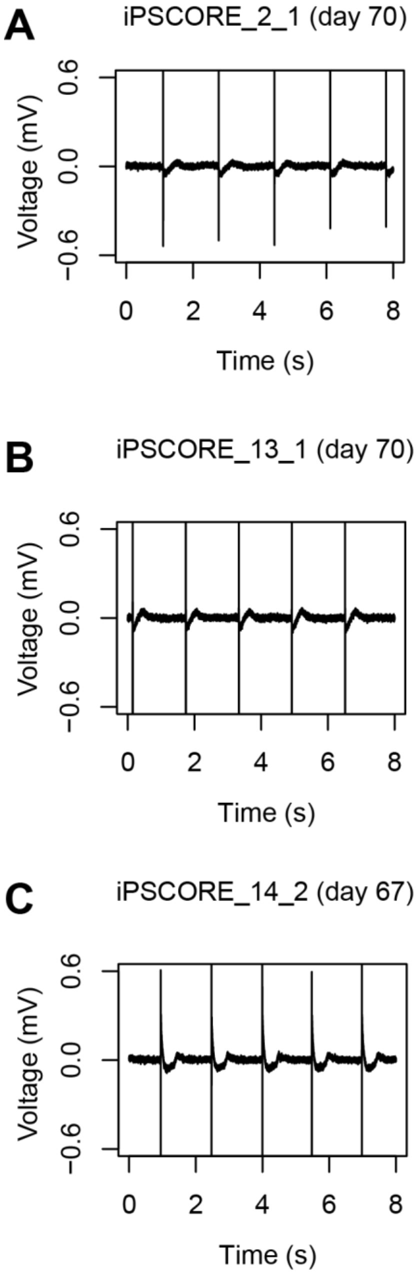

Field potential measured using MEA iPSC-derived cardiomyocytes: (A) iPSCORE_2_1; (B) iPSCORE_13_1; and (C) iPSCORE_14_2.

Figure S5. Analysis of Sendai virus clearance in iPSC lines. Related to Figure 2.

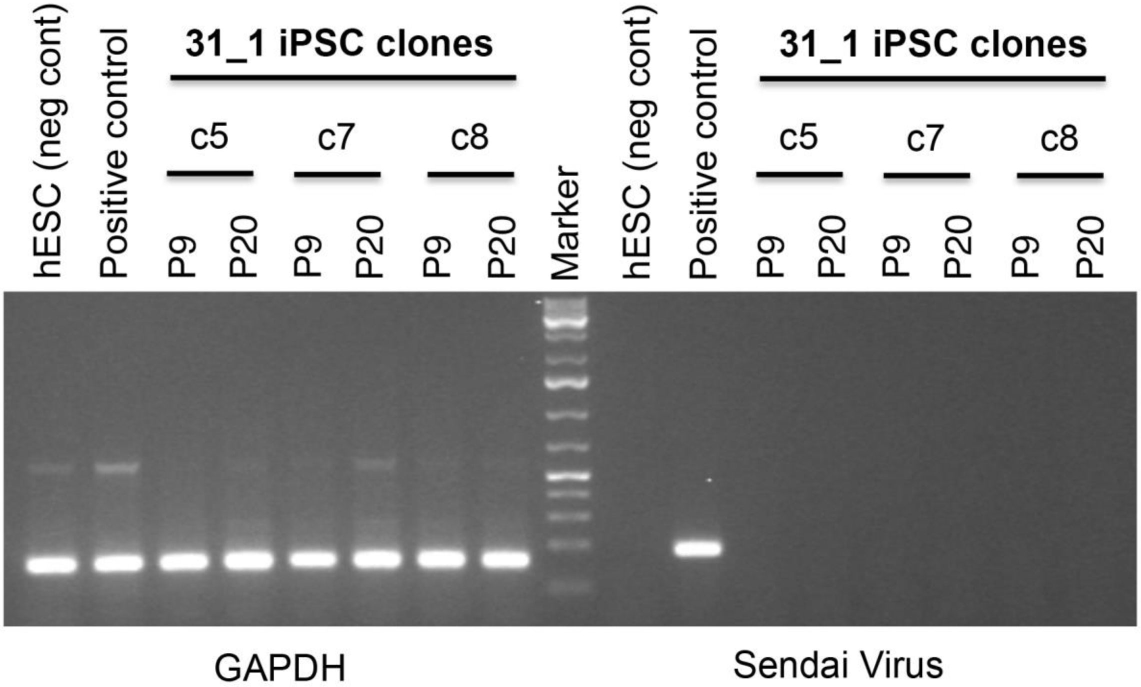

RT-PCR analysis of Sendai virus and GAPDH (housekeeping gene control) levels in iPSC lines generated from individual 31_1 in the cohort. Three clonal lines (e.g. clone 5 = c5) were assessed at passage 9 (P9) and passage 20 (P20) for the presence of Sendai virus, with all clonal lines showing clearance of Sendai virus by P9. All iPSC lines in the described study were cultured and frozen at P12 or later.

SUPPLEMENTARY TABLE LEGENDS

Table S1. Phenotype information for participants in iPSCORE collected at enrollment and identifiers for cell lines and genomic data. Related to Figure 1.

Table S1A. Phenotype information for participants in iPSCORE.
iPSCORE_ID indicates family and individual number. Subject UUID is an assigned Universal Unique Identifier (UUID) for the subject. Family ID stratifies the subject with related family members. Sex and age at the time of enrollment of the subject are given. For the 39 patients recruited from the UCSD Sulpizio Cardiovascular Center, the category of heart disease is given as the primary diagnosis, other heart diagnoses, comments and disease ontology code. Self-reported race/ethnicity (column K) obtained as a free response written by the subject or physician (denoted by asterisk) was translated into one of seven groups (African American, Asian, European, Hispanic, Indian, Middle Eastern, and Multiple ethnicities reported) and defined as recorded ethnicity grouping (column L). The expected superpopulation (SP) from the 1000 Genomes Project was generated from the recorded ethnicity and compared to the observed superpopulation (column M). We observed no mismatches. Hispanic individuals were considered a match if they matched either EUR or AMR. Middle Eastern individuals were considered a match if they matched EUR, SAS, or AFR. In the case of mixed race/ethnicity, individuals were considered to match if the top match matched one of their reported race/ethnicity groups or if their position on the PCA plots (Figure 1G) was consistent with a mixture of their reported ancestries.

Table S1B. Putative genetic variants underlying cardiac diseases
Variants identified by whole genome sequencing in cardiomyopathy or arrhythmia disease-associated genes are reported. iPSCORE_ID, Subject_UUID, and Family ID are as in Table S1A. rsID is the dbSNP identifier for the variant. Chromosome, Position, Reference allele, and

Alternate allele are reported for genome build hg19. Genotype indicates the genotype for the individual (0/0 = homozygous reference, 0/1 = heterozygous, 1/1 = homozygous alternate). The Gene indicates the affected gene, with Coding sequence change and Amino acid change indicating the impact of the variant on the gene and protein, respectively. The ClinVar clinical significance lists the pathogenicity reported to ClinVar. The ClinVar RCVaccession reports the accession numbers in ClinVar for these variants.

Table S1C. Table linking identifiers for iPSCORE participants, cell lines and genomic data. The IPSCORE IDs and UUID Subject IDs are given (columns A, B). ID and passage and clone information about iPSC lines (columns C, D, E) and the WiCell ID (column F). UUID for WGS data and DNA tissue source (columns G, H). UUIDs for RNA-seq and genotype array data (columns I, J). The UUID identifiers are also referenced in the dbGaP dataset.

Table S2. Pluripotency of 213 iPSC lines calculated by PluriTest-RNAseq. Related to Figure 2.
For each of the 213 iPSC lines that underwent RNA-seq, we provide UUID Subject IDs, iPSCORE IDs, RNA-seq UUID (as deposited to dbGAP), novelty score, pluripotency score and final assessment of pluripotency as PluriTest-RNAseq (213 lines). In total, 206 lines have high pluripotency and low novelty scores calculated by PluriTest-RNAseq. The other seven have values slightly below the 98% sensitivity and 100% specificity thresholds, suggesting that, while they are likely pluripotent, additional evaluations would be needed to confirm their pluripotency. These 7 lines overall showed high genomic integrity and a subset differentiated to cardiomyocytes at a similar rate as other passing cell lines supporting their pluripotency.

Table S3: CNV analysis of the 222 iPSC lines. Related to Figure 3.

Table S3A. List of the 199 detected CNVs (see Experimental Procedures).
The iPSC name is given (column B), the CNV type (column C) and whether it is chromosomal (a chromosomal arm or full chromosome) or subchromosomal (column D). Coordinates of the detected CNV, length of CNV, and the method of detection ("Primary Detection Method") are reported. Each CNV was detected either by automatic analysis paired with a germline sample (with Nexus) or by visual analysis and manual curation. Some of the manually curated CNVs did not pass the automatic detection cutoff points in Nexus, however they displayed typical clear CNV patterns and so were retained in the final CNV set. Nexus-called CNVs that were not clear after visual inspection of the log R ratios and B-allele frequencies were removed. Segmented neighboring CNVs were merged into a single CNV as appropriate. The cytoband containing the CNV (column J) and if the CNV was in one of the five regions significantly enriched for CNVs (column K) is given.

Table S3B. iPSC lines containing CNVs at passage (P12) examined at an earlier passage (P3).
iPSCORE ID, CNV type and chromosome position are given (columns A, B, C). The clone ID for the first (column D), second (column E) and third clones (column F) from the same subject (IPSCORE ID) are given. Columns G, H and I indicate whether the CNV was present by visual inspection in the same clone at an earlier passage (P3), the second clone or third clone. A "--" indicates that no data is available.

Table S3C. Significantly recurrent CNV regions.
The chromosome coordinates, length and cytoband location of the five regions that were enriched for CNVs are given. The type of alteration, number of genes involved and minimum STAC frequency P-value are reported.

Table S4: Gene Ontology terms associated with four gene cluster groups in time course study. Related to Figure 4C.

We tested 20,178 GO terms included in GOseq v. 1.24.0 (March 30 2016) separately for each cluster described in Figure 4C, corresponding to genes active at Day 0, Day 2, Day 5 and Day 15 of cardiomyocyte differentiation. For each GO term (column A), we provide the GO branch ("Biological Process": BP; "Molecular Function": MF; or "Cellular Component": CC, column B), the GO description (column C), the analyzed day (column D), the number of genes in the cluster included in the GO term and outside the GO term (column E and F, respectively) and of all the remaining human genes, the number in and not in the cluster (column G and H). For each GO term, log2 ratio (column I) was calculated by fraction of genes included in the GO term in the cluster and not in the cluster. P-values (column J) were calculated using GOseq and adjusted using Bonferroni method (column K). GO terms significant at an FDR < 0.05 are reported.

Table S5: Genotype data from 222 germline samples at 2,571 GWAS SNPs measured by the HumanCoreExome BeadChip. Related to Figure 5.

The chromosome, coordinate and SNP ID (rsID) are listed (columns A, B, C). The reference and alternate alleles as well as the risk allele as reported by the NHGRI GWAS Catalog, associated disease/trait and PubMed ID and nearest mapped gene are given (columns D, E, F, G, H, I). When a SNP was associated with multiple traits, it is listed multiple times. The genotype of each individual is listed as the number of risk alleles (0 = non-risk/non-risk, 1 = risk/non-risk, 2 = risk/risk).

SUPPLEMENTAL EXPERIMENTAL PROCEDURES

Cell culture and human iPSC generation

Cultures of primary dermal fibroblast cells were generated by mechanical dissection and enzymatic digestion of the punch biopsy tissue, followed by adherent outgrowth on gelatin coated 24-well plates as previously described (Israel et al., 2012). The primary fibroblast cultures were expanded for approximately 3 passages prior to cryopreservation in advance of reprogramming. The fibroblasts were thawed and plated at a density of 250K cells/well of 6-well plate, then infected with the Cytotune Sendai virus (Life Technologies) per manufacturer's protocol to initiate reprogramming. The Sendai infected cells were maintained with 10% FBS/DMEM (Invitrogen) for Days 4-7 until the cells recovered and repopulated the well. These cells were then enzymatically dissociated using TrypLE (Life Technologies) and seeded onto a 10-cm dish pre-coated with mitotically inactive-mouse embryonic fibroblasts (MEFs) at a density of 500K/dish and maintained with hESC medium, as previously described (Ruiz et al., 2010). Emerging iPSC colonies were manually picked after Day 21 and maintained on Matrigel (BD Corning) with mTeSR1 medium (Stem Cell Technologies) as previously described (Panopoulos et al., 2012). Multiple independently established iPSC lines (i.e. referred to as clones) were derived from each individual (on average three clones), with a minimum of two clones frozen at passage three as backup stocks, and one clone cultured to late passage (typically passage 12) before freezing ten vials for banking at WiCell Research Institute (WiCell IDs in Table S1C). Sendai virus clearance typically occurred at or before P9, and was not detected in the iPSC lines at the P12 stage of cryopreservation (Figure S5).

Sendai Virus Clearance Assessment

Total RNA was harvested from iPSC lines at indicated passages using Trizol Reagent (Invitrogen). For the positive control, RNA was isolated from fibroblasts 3 days post Sendai virus infection. RNA harvested from human embryonic stem cells (ESCs) was used as an uninfected pluripotent control. Collected RNA was reverse transcribed using the SuperScript II Reverse Transcriptase kit (Invitrogen) according to manufacturer's protocol. PCR was performed using primers to Sendai virus as described in the Cytotune Sendai Virus kit (Life Technologies). GAPDH (primers described in (Panopoulos et al., 2011)) was used as an internal loading control.

Flow Cytometry Analysis

Fifty iPSC lines were evaluated by flow cytometry for pluripotent marker expression. Prior to freezing, cells were brought to approximately 60% confluence and individualized with TrypLE (Thermo Fisher). After washing, sites were blocked using Fcblock (Biolegend) for 30 minutes. Cells were then resuspended in buffer (1% BSA/PBS) and stained using Tra-1-81 (Alexa Fluor 488 anti-human, Biolegend), SSEA-4 (PE anti-human, Biolegend), or the appropriate isotype controls for one hour at RT. Cells were resuspended in flow buffer (1%BSA/PBS) and analyzed using a BD FACSCanto Flow Cytometer (10,000 events counted) and the FACSDiva software (BD). They were scored as pluripotent if they were found to be 95% positive for both Tra-1-81 and SSEA-4. Pluripotency was also examined using RNA-seq data (see below).

RNA-Seq

Total RNA was extracted from pellets of $1x10^6$ cells frozen in RLT plus buffer in the Qiagen AllPrep DNA/RNA Mini kit (Qiagen Cat# 80204) and eluted in molecular grade H_2O. RNA concentration was measured by Nanodrop and integrity was determined using the Agilent 2200 TapeStation System. A total of 213 mRNA libraries were prepared by Illumina Truseq Stranded and sequenced by HiSeq2500, to an average of 20M 100bp read-pairs per sample. Reads were

aligned using STAR (2.5.0a) to the hg19 reference and a splice junction database built from the Gencode v19 gene annotation (Harrow et al., 2012). Duplicates were marked using biobambam2 (2.0.21) and transcript and gene-based expression values, including read count and transcripts per million (TPM), were obtained using the package RSEM (Li and Dewey, 2011). Read counts were normalized using variance stabilizing transformation (VST) using DeSeq2 (Love et al., 2014). VST-normalized expression levels were transformed to Z-scores by subtracting the mean value of each gene and dividing by the standard deviation.

To examine the similarity of the iPSCs to other iPSCs, embryonic stem cells, and fibroblasts, we extracted the expression levels of 34 genes known to be relevant based on the TaqMan hPSC Scorecard Assay (Choi et al., 2015), and compared their expression profiles between our 213 iPSCs and 73 publicly available cell lines from the Gene Expression Omnibus (GEO) series GSE73211 (21 iPSCs, 35 hESCs and 17 fibroblasts) (Choi et al., 2015) by hierarchical clustering and generating a heatmap using the Pheatmap R package (Figure 2A).

For the cardiomyocyte differentiation time course experiment, RNA for three iPSC lines (2_2, 2_3 and 2_9) was collected in biological triplicates at day 2, 5, 9 and 15 (Paige et al., 2012). The 500 autosomal genes with highest standard deviation in expression levels were used for hierarchical clustering and to generate a heatmap. Four gene groups, roughly corresponding to genes active in iPSC and in cells at day 2, 5 and 15, were determined using the function cutree (k = 4) in R. Functional enrichment for each group was determined using GOseq v. 1.24.0 (March 30 2016) (Young et al., 2010) on 20,178 GO terms including 20,345 human genes. P-values from GOseq were adjusted for multiple testing hypothesis using the Bonferroni method (Table S4).

PluriTest-RNAseq

PluriTest-RNAseq uses an extended and modified version of the array based PluriTest workflow (Muller et al., 2011). This algorithm generates a Pluripotency score that is the result of a logistic regression model that measures the probability of a line to be pluripotent, as well as a Novelty score that indicates the deviation of an iPSC line from a normal pluripotent line, with larger values indicating gene expression patterns usually not observed in iPSC. According to the PluriTest algorithm, high quality pluripotent lines have Pluripotency Score ≥ 20 and Novelty Score ≤ 1.67. These thresholds allow us to label a sample as "pluripotent" (Muller et al., 2011). The critical update and modification to the PluriTest procedure is the construction of a TPM (Transcripts Per Kilobase Million) based "virtual array" for each sample. Testing PluriTest-RNAseq with RNA-seq data from pluripotent and non-pluripotent cell lines reveals that it functions with similar level of specificity and selectivity as the original PluriTest array-based procedure. A complete account will be described in full elsewhere (manuscript in preparation FM, RW, BS, JL).

Briefly, the 213 samples were processed using the pseudo aligner Salmon version 0.7.2 (Patro et al., 2015) against the GRch38.p7 Gencode v25 transcriptome sequences (gencodegenes.org). A "virtual array" probe set was generated by locating the exact match probe sequences from the HT12v4 Illumina array in the Gencode v25 transcriptome sequences. This "virtual array" probe set was pruned for probes with either no match in the Gencode v25 transcriptome, or that had large model errors. We assessed the error in the "virtual array" model by performing a t-test between the expression in pluripotent samples of GSE53094 (processed as above) and the pluripotent samples in the original training set. Thus, probes with no hits in Gencode v25 or with a foldchange >0.5 and a p.value < 0.05 according to the t-test were removed, leaving 10,079 probes. A sample "virtual-array" was created by summing the Salmon

TPM for transcripts with matches to each of these 10,079 probe sequences. As previously described (Muller et al., 2011), the data was then transformed into a standard R-lumiBatch object, quantile normalized, and tested with the predictive model. This yields the pluripotency score and novelty score which reflect how similar an iPSC is to those in the original data model. Variations in the probes used can create some subtle scoring differences between PluriTest-RNAseq and PluriTest.

Previously, we set the Pluripotency and Novelty Score thresholds for the array based version of PluriTest to separate high quality iPSC lines from those with quantifiable deviations from the pluripotent phenotype (e.g. germ cell tumor cell lines and parthenogenetic stem cell lines) with 98% sensitivity and 100% specificity (Müller 2011). Cell lines that have unusually high novelty scores indicate that these test samples should be additionally evaluated for epigenetic or genetic abnormalities or unwanted differentiation. Cell lines that have pluripotency scores just below the cutoff threshold may need further investigation to confirm pluripotency. In this manuscript, for cell lines not passing either threshold, copy number estimation based on the genotype array analysis were examined to rule out genetic abnormalities and cardiomyocyte differentiation was examined to support pluripotency.

HumanCoreExome array processing and selection of SNPs

Genomic DNA was isolated from iPSC lines (AllPrep DNA/RNA Mini Kit, Qiagen) and from blood or fibroblast germline samples (DNEasy Blood & Tissue Kit), normalized to 200 ng, hybridized in pairs to Illumina HumanCoreExome arrays (Illumina), and stained per Illumina’s standard protocol. The stained Beadchips were then scanned on the Illumina HiScan and processed in GenomeStudio (v 1.9.4). Genotypes were converted from Illumina TOP orientation to genome orientation (b37) using the humancoreexome-12v1-1_a-b37-strand and

HumanCoreExome-24v1-0_A-b37-strand files generated through the Wellcome Trust Center for Human Genetics (http://www.well.ox.ac.uk/~wrayner/strand/). Sites reported as "Cautious Sites" (http://genome.sph.umich.edu/wiki/Exome_Chip_Design#Cautious_Sites) were removed. Sites were annotated to dbSNP 138 identifiers using The Genome Analysis Toolkit (GATK) (DePristo et al., 2011). We observed an average call rate of 99.2% across the 444 arrays.

To examine family relationships, estimate ancestry, and to confirm iPSC sample identity by comparison with the matched germline sample, we used a subset of the array SNPs comprised of 90,099 SNPs that were in linkage equilibrium (r2 < 0.2), common (MAF > 0.05), and present by dbSNP rsID in the KGP Phase 3 data (downloaded from ftp://ftp.1000genomes.ebi.ac.uk/vol1/ftp/release/20130502/).

Family relatedness

Genotypes from individuals that were part of a family with two or more people were compared to determine if the reported relationships matched the kinship coefficient (PI_HAT) calculated using PLINK (Purcell et al., 2007) (v1.09). Pairs of individuals that differed in expected and reported kinship coefficient by more than 0.1 were investigated and compared to other pairwise measures within the family when available to verify unexpected relationships. We observed four pairs with higher deviations from expected, but these relationships were verified through relationships within the family. These pairs all included individuals of admixed ancestry, which is known to bias measures of pIBD (Thornton et al., 2012).

Ancestry estimation

We estimated the ancestry of each participant by comparing their genomes to those of individuals in the 1000 Genomes Project (KGP) using a previously published approach (Smith et al., 2014). We identified the KGP super population group (AFR: African, AMR: Admixed

American, EAS: East Asian, EUR: European, SAS: South Asian) to which each iPSCORE participant was most similar. First, KGP individuals were clustered using principal components analysis (--pca in PLINK) and iPSCORE individuals were mapped onto these components by applying the “--within” command. To identify which super population the iPSCORE individual best matched, we used using linear discriminant analysis (lda command in MASS package (Venables et al., 2002) in R) with the first 20 principal components of the KGP individuals as a training set. We compared predicted groups to self-reported ethnicity, classifying recorded ethnicity groupings by the super population most likely to match. We considered a match for the following pairs: African American – AFR; Asian – EAS; European – EUR, Hispanic – EUR or AMR; Indian – SAS; Middle Eastern -- EUR, SAS, or AFR. In the case of mixed race/ethnicity, individuals were considered to match if the top match matched one of their reported race/ethnicity groups or if their position on the PCA plots was consistent with a mixture of their reported ancestries.

Putative genetic variants underlying cardiac diseases

To identify genetic variants that could potentially be associated with the reported cardiac diseases, we examined whole genome sequence data (DeBoever et al., In Press) (Table S1B). For probands with cardiac disease and their family members, we examined genetic variation within genes that are part of the GeneDx Cardiomyopathy or Arrhythmia Panels (www.genedx.com) for individuals with reported cardiomyopathy or arrhythmia diseases, respectively. The resulting genotypes were annotated using ClinVar (Landrum et al., 2016) and variants associated with “pathogenic” or “likely pathogenic” reports were examined. Variants that were previously reported as pathogenic, but were later reported as “benign” or “likely benign” by GeneDx were

excluded. Variants that were not reported to be associated with the proband's reported disease or did not segregate with disease in the family were also excluded.

Confirming iPSC sample identity and genetic sex

All iPSC lines were compared to their respective germline sample using the –genome command in PLINK and samples were flagged if the kinship coefficient (PI_HAT) was less than 0.95. Genetic sex was estimated using the command "--check-sex" in PLINK and compared to self or physician report.

Copy Number Variation Determination

Raw scan data were processed by Genome Studio (Illumina, Inc) using the supplied clusterfiles for SNP calling on the Human Core Exome arrays (average call rate 0.99, GenCall threshold 0.15). Processed SNP array data for the 222 iPSCs were subjected to both manual and computerized analysis for somatic CNVs. For computerized analysis, genotype data were exported to Nexus CN (version 7.5) where CNV calling was carried out with the hg19/GRCh37 reference version of the human genome. The X and Y chromosomes were removed due to the complexity of reliably determining copy number in these copy variable and highly repetitive chromosomes. A descriptor sheet was supplied with the 222 sample pairings for germline to corresponding iPSC results files. The Nexus files and settings used were: Systematic Correction File: Catlg_ILM_HumanCoreExome-12v1-1_B_20140311.bed_hg19_ilum_correction.txt (as supplied by Biodiscovery Inc), Recenter Probes to Median, Analysis performed with the SNPRank Segmentation algorithm. Significance threshold 5.0E-9, Min Number of probes per segment = 7, High Gain 0.75, Gain 0.22, Loss -0.2, Big Loss -1.1. Called CNVs were size filtered with those <100kb removed. This is the conservative limit of detection for CNVs when 7 probes are used with a spacing (90% of probes) of 14.3kb. CNV regions called LOH were

excluded if they also were listed as copy number loss, resulting in 272 regions. We then performed systematic manual inspection of each Nexus called CNV, visualizing the B-allele frequencies (proportion of A and B alleles at each genotype) and log R ratios (ratio of observed to expected intensities) for each iPSC and its respective germline sample. These plots were visually scanned by a trained operator and Nexus called CNVs that were not visually consistent with a CNV based on B-allele frequencies and log R rations were removed. In addition, manual inspection of the entire genome (including sex chromosomes) was performed for each sample compared to the respective germline. This complementary approach, which is good for calling large CNVs, identified 31 CNVs, of which, 10 were also called by Nexus. The Nexus Allelic-Imbalance and LOH CNV classes were combined into one group called LOH for Figures 3C and S3.

Identification of clustered CNVs

To test for significant clustering of CNVs across multiple samples, we used the STAC program (Diskin et al., 2006). Briefly, considering each chromosome independently the algorithm tries to identify a set of aberrations with a higher frequency than what is expected to occur randomly. We partitioned each chromosome into 100kb regions and indicated whether a CNV overlapped each region for each sample. We then performed 1,000 permutations for each chromosome to identify locations with CNV frequencies higher than expected by chance (frequency P-value < 0.05). Regions that were adjacent to each other were merged and the minimum P-value for the region reported (Table S3C).

Differentiation of iPSC lines into cardiomyocytes

Differentiation into cardiomyocytes was performed according to the protocol described by Lian et al. (Lian et al., 2013). For the time course experiment, three cell lines were differentiated in

three six-well plates each, and each plate represented a biological replicate. From each six-well plate, one well was harvested on day 0, 2, 5, 9, and 15, corresponding to previously described cardiac differentiation stages (Paige et al., 2012). For the molecular study of *KCNH2,* iPSC lines from seven family members were seeded to T150 flasks and differentiated into cardiomyocytes to day 15 in two independent experiments per line (biological replicates). Cells were dissociated using Accutase; one million cells per sample were lysed and stored in RLT plus buffer (Qiagen) for RNA extraction.

Immunohistochemistry and immunofluorescence

For sarcomeric alpha-actinin (ACTN1) and connexin 43 (Cx43) immunofluorescence, day 34 iPSC-CMs were cultured on 0.1 % gelatin-coated glass-bottom plates for 48-72hrs, and then fixed with 4% paraformaldehyde at room temperature (RT) for 20 mins. Fixed iPSC-CMs were permeabilized in 0.1% Triton X-100 for 8 mins at RT, then blocked in 5% bovine serum albumin for 30 mins at RT, and then incubated overnight at 4 °C with rabbit polyclonal anti-Cx43 antibody (Invitrogen, 710700, dilution 1:1,000) and mouse monoclonal anti-ACTN1 antibody (Sigma, A7811, dilution 1:200). Cells were incubated with donkey anti-rabbit Alexa Fluor 488 (Invitrogen, A-21206, dilution 1:800) and goat anti-mouse Alexa Fluor 568 (Invitrogen, A-11004, dilution 1:800**)** secondary antibodies for 45 mins at RT. Nuclei were counterstained with DAPI. Cells were washed 3x in PBS between each step. Olympus FluoView FV1000 confocal microscope was used for imaging. For myosin light chain 2a (MLC2a) staining, day 15 cardiomyocytes were seeded onto chambers with microscope slides (Millipore) and cultured overnight. After fixation with 4% PFA, cells were blocked and permeabilized for 1 h at 37 °C with 5% BSA, 5% serum, and 0.1% Triton X-100. Cells were incubated with 1:200 dilution of mouse monoclonal anti-myosin light chain 2a (MLC2a) antibody (Synaptic Systems, 311011)

overnight at 4 °C; with secondary antibody (AlexaFluor 488) for 2 h at RT; and 20 min with DAPI. Leica SP5 confocal microscope was used for imaging.

Multi-electrode array analysis (MEA)

Beating, 25 days old iPSC-derived cardiomyocyte monolayers in 6-well plates were dislodged using a cell scraper and dissociated into small clumps by gently pipetting with a 1-ml pipette. Cell clumps were re-plated in MEA plates (Axion Biosystems) previously coated with Matrigel and allowed to settle for 24-48 hours. Electrophysiological activity was then assayed and recorded for ~1 minute using MEA Maestro apparatus (Axion Biosystems). Cells were incubated with Isoproterenol 0.01µM at 37˚C immediately before the second MEA analysis. We extracted the field potential recording from the same electrode before and after treatment and plotted the traces (Figures 4E, S4). Beat periods were calculated from the traces of 8 electrodes of a well (Figure 4F) and plotted using R.

Analysis of *KCNH2* expression by qPCR

One µg of total RNA from iPSC-derived cardiomyocytes was retro-transcribed using SuperScript III First-Strand Synthesis System (Thermo Scientific) using oligo dT primers in 20 µl reactions. RT-qPCR reactions were performed in 15 µl using 3.5 µl of a 1:50 cDNA dilution using KAPA SYBR Fast qPCR Kit (KPA Biosystem) and run on LightCycler 480 (Roche). Primers were designed and tested to amplify specifically the wild-type or the mutated allele of *KCNH2* transcript (c.3003G>A) using the following primers: forward: GTGTCCAACATTTTCAGCTTCTT**G** (wt) or GTGTCCAACATTTTCAGCTTCTT**A** (mutated), reverse: AGTGGCCATGTCTGCACTC (common to wild type and mutated). These allele-specific primers were designed using the WASP tool (Wangkumhang et al., 2007). *KCNH2* C_ts were normalized to *GAPDH* (forward primer: TGTTGCCATCAATGACCCCTT,

reverse primer: CTCCACGACGTACTCAGCG) and expressed as $\Delta\Delta C_t$ with respect to the average ΔCt_s.

GWAS loci genotypes

GWAS-associated loci were downloaded from the NHGRI GWAS Catalog (https://www.genome.gov/26525384, 2/23/2015). SNPs were retained if they had a reported P-value < 5 x 10^{-8}; a reported risk allele of A, C, T, or G; and a current dbSNP ID. When a SNP was associated multiple times with the same phenotype, the most significant report was used. This resulted in 4,528 SNPs, 600 phenotypes, and 5,514 SNP-phenotype relationships. Of these, 2,858 SNPs were present on the HumanCoreExome BeadChip. We further excluded sites that were ambiguous (C/G or A/T SNPs), had a Hardy-Weinberg equilibrium P-value < 10^{-7} in our study, and a call rate of <90% in the germline samples, resulting in 2,517 SNPs, 487 phenotypes, and 3,350 SNP-phenotype relationships (Table S5). Genotypes from the HumanCoreExome arrays hybridized with germline DNA were then tabulated to identify the number of individuals carrying the risk/risk, risk/non-risk, and non-risk/non-risk genotypes.

REFERENCES

Choi, J., Lee, S., Mallard, W., Clement, K., Tagliazucchi, G.M., Lim, H., Choi, I.Y., Ferrari, F., Tsankov, A.M., Pop, R., *et al.* (2015). A comparison of genetically matched cell lines reveals the equivalence of human iPSCs and ESCs. Nat Biotechnol *33*, 1173-1181.

DeBoever, C., Li, H., Jakubosky, D., Arias, A., Olson, K.M., Huang, H., Biggs, W., Sandoval, E., Matsui, H., Ren, B., *et al.* (In Press). Genetic Regulation of Gene Expression in Human Induced Pluripotent Stem Cells. Cell stem cell.

DePristo, M.A., Banks, E., Poplin, R., Garimella, K.V., Maguire, J.R., Hartl, C., Philippakis, A.A., del Angel, G., Rivas, M.A., Hanna, M., *et al.* (2011). A framework for variation discovery and genotyping using next-generation DNA sequencing data. Nat Genet *43*, 491-498.

Diskin, S.J., Eck, T., Greshock, J., Mosse, Y.P., Naylor, T., Stoeckert, C.J., Jr., Weber, B.L., Maris, J.M., and Grant, G.R. (2006). STAC: A method for testing the significance of DNA copy number aberrations across multiple array-CGH experiments. Genome Res *16*, 1149-1158.

Harrow, J., Frankish, A., Gonzalez, J.M., Tapanari, E., Diekhans, M., Kokocinski, F., Aken, B.L., Barrell, D., Zadissa, A., Searle, S., *et al.* (2012). GENCODE: the reference human genome annotation for The ENCODE Project. Genome research *22*, 1760-1774.

Israel, M.A., Yuan, S.H., Bardy, C., Reyna, S.M., Mu, Y., Herrera, C., Hefferan, M.P., Van Gorp, S., Nazor, K.L., Boscolo, F.S., *et al.* (2012). Probing sporadic and familial Alzheimer's disease using induced pluripotent stem cells. Nature *482*, 216-220.

Landrum, M.J., Lee, J.M., Benson, M., Brown, G., Chao, C., Chitipiralla, S., Gu, B., Hart, J., Hoffman, D., Hoover, J., *et al.* (2016). ClinVar: public archive of interpretations of clinically relevant variants. Nucleic acids research *44*, D862-868.

Li, B., and Dewey, C.N. (2011). RSEM: accurate transcript quantification from RNA-Seq data with or without a reference genome. BMC Bioinformatics *12*, 323.

Lian, X., Zhang, J., Azarin, S.M., Zhu, K., Hazeltine, L.B., Bao, X., Hsiao, C., Kamp, T.J., and Palecek, S.P. (2013). Directed cardiomyocyte differentiation from human pluripotent stem cells by modulating Wnt/beta-catenin signaling under fully defined conditions. Nat Protoc *8*, 162-175.

Love, M.I., Huber, W., and Anders, S. (2014). Moderated estimation of fold change and dispersion for RNA-seq data with DESeq2. Genome biology *15*, 550.

Muller, F.J., Schuldt, B.M., Williams, R., Mason, D., Altun, G., Papapetrou, E.P., Danner, S., Goldmann, J.E., Herbst, A., Schmidt, N.O., *et al.* (2011). A bioinformatic assay for pluripotency in human cells. Nat Methods *8*, 315-317.

Paige, S.L., Thomas, S., Stoick-Cooper, C.L., Wang, H., Maves, L., Sandstrom, R., Pabon, L., Reinecke, H., Pratt, G., Keller, G., *et al.* (2012). A temporal chromatin signature in human embryonic stem cells identifies regulators of cardiac development. Cell *151*, 221-232.

Panopoulos, A.D., Ruiz, S., Yi, F., Herrerias, A., Batchelder, E.M., and Izpisua Belmonte, J.C. (2011). Rapid and highly efficient generation of induced pluripotent stem cells from human umbilical vein endothelial cells. PLoS One *6*, e19743.

Panopoulos, A.D., Yanes, O., Ruiz, S., Kida, Y.S., Diep, D., Tautenhahn, R., Herrerias, A., Batchelder, E.M., Plongthongkum, N., Lutz, M., *et al.* (2012). The metabolome of induced pluripotent stem cells reveals metabolic changes occurring in somatic cell reprogramming. Cell Res *22*, 168-177.

Patro, R., Duggal, G., and Kingsford, C. (2015). Accurate, fast, and model-aware transcript expression quantification with Salmon. BioRxiv.

Purcell, S., Neale, B., Todd-Brown, K., Thomas, L., Ferreira, M.A., Bender, D., Maller, J., Sklar, P., de Bakker, P.I., Daly, M.J., *et al.* (2007). PLINK: a tool set for whole-genome association and population-based linkage analyses. Am J Hum Genet *81*, 559-575.
Ruiz, S., Brennand, K., Panopoulos, A.D., Herrerias, A., Gage, F.H., and Izpisua-Belmonte, J.C. (2010). High-efficient generation of induced pluripotent stem cells from human astrocytes. PLoS One *5*, e15526.
Smith, E.N., Jepsen, K., Arias, A.D., Shepard, P.J., Chambers, C.D., and Frazer, K.A. (2014). Genetic ancestry of participants in the National Children's Study. Genome biology *15*, R22.
Thornton, T., Tang, H., Hoffmann, T.J., Ochs-Balcom, H.M., Caan, B.J., and Risch, N. (2012). Estimating kinship in admixed populations. Am J Hum Genet *91*, 122-138.
Venables, W.N., Ripley, B.D., and Venables, W.N. (2002). Modern applied statistics with S, 4th edn (New York: Springer).
Wangkumhang, P., Chaichoompu, K., Ngamphiw, C., Ruangrit, U., Chanprasert, J., Assawamakin, A., and Tongsima, S. (2007). WASP: a Web-based Allele-Specific PCR assay designing tool for detecting SNPs and mutations. BMC Genomics *8*, 275.
Young, M.D., Wakefield, M.J., Smyth, G.K., and Oshlack, A. (2010). Gene ontology analysis for RNA-seq: accounting for selection bias. Genome biology *11*, R14.
