# Supplementary material for: iPSCORE: A Resource of 222 iPSC Lines Enabling Functional Characterization of Genetic Variation across a Variety of Cell Types
Source: Stem Cell Reports. 2017 Apr 6;8(4):1086–100. doi: 10.1016/j.stemcr.2017.03.012 (PMC5390244; doi:10.1016/j.stemcr.2017.03.012)
Supplement: Document S2. Article plus Supplemental Information [file mmc7.pdf]

Stem Cell Reports
Resource
ISSCR
OPEN ACCESS

iPSCORE: A Resource of 222 iPSC Lines Enabling Functional Characterization of Genetic Variation across a Variety of Cell Types

Athanasia D. Panopoulos,[1,11] Matteo D'Antonio,[2] Paola Benaglio,[3] Roy Williams,[3,4] Sherin I. Hashem,[5] Bernhard M. Schuldt,[6] Christopher DeBoever,[7] Angelo D. Arias,[3] Melvin Garcia,[2] Bradley C. Nelson,[5] Olivier Harismendy,[5,7] David A. Jakubosky,[8] Margaret K.R. Donovan,[7] William W. Greenwald,[7] KathyJean Farnam,[2] Megan Cook,[2] Victor Borja,[2] Carl A. Miller,[2] Jonathan D. Grinstein,[5,8] Frauke Drees,[3] Jonathan Okubo,[2] Kenneth E. Diffenderfer,[9] Yuriko Hishida,[1] Veronica Modesto,[9] Carl T. Dargitz,[9] Rachel Feiring,[9] Chang Zhao,[2] Aitor Aguirre,[5] Thomas J. McGarry,[5] Hiroko Matsui,[2] He Li,[2] Joaquin Reyna,[2] Fangwen Rao,[5] Daniel T. O'Connor,[5] Gene W. Yeo,[2,10] Sylvia M. Evans,[5] Neil C. Chi,[2,5,8] Kristen Jepsen,[2] Naoki Nariai,[3] Franz-Josef Müller,[6] Lawrence S.B. Goldstein,[10] Juan Carlos Izpisua Belmonte,[1] Eric Adler,[5] Jeanne F. Loring,[4] W. Travis Berggren,[9] Agnieszka D'Antonio-Chronowska,[2] Erin N. Smith,[3] and Kelly A. Frazer[2,3,*]
[1]Gene Expression Laboratory, Salk Institute for Biological Studies, La Jolla, CA 92037, USA
[2]Institute for Genomic Medicine
[3]Department of Pediatrics
University of California, San Diego, La Jolla, CA 92093, USA
[4]Center for Regenerative Medicine, The Scripps Research Institute, La Jolla, CA 92037, USA
[5]Department of Medicine, University of California, San Diego, La Jolla, CA 92093, USA
[6]Zentrum für Integrative Psychiatrie, Universitätsklinikum Schleswig-Holstein, 24105 Kiel, Germany
[7]Bioinformatics and Systems Biology Graduate Program
[8]Biomedical Sciences Graduate Program
University of California, San Diego, La Jolla, CA 92093, USA
[9]Stem Cell Core, Salk Institute for Biological Studies, La Jolla, CA 92037, USA
[10]Department of Cellular and Molecular Medicine, University of California, San Diego, La Jolla, CA 92093, USA
[11]Present address: Department of Biological Sciences, University of Notre Dame, Notre Dame, IN 46556, USA
*Correspondence: kafrazer@ucsd.edu
http://dx.doi.org/10.1016/j.stemcr.2017.03.012

SUMMARY

Large-scale collections of induced pluripotent stem cells (iPSCs) could serve as powerful model systems for examining how genetic variation affects biology and disease. Here we describe the iPSCORE resource: a collection of systematically derived and characterized iPSC lines from 222 ethnically diverse individuals that allows for both familial and association-based genetic studies. iPSCORE lines are pluripotent with high genomic integrity (no or low numbers of somatic copy-number variants) as determined using high-throughput RNA-sequencing and genotyping arrays, respectively. Using iPSCs from a family of individuals, we show that iPSC-derived cardiomyocytes demonstrate gene expression patterns that cluster by genetic background, and can be used to examine variants associated with physiological and disease phenotypes. The iPSCORE collection contains representative individuals for risk and non-risk alleles for 95% of SNPs associated with human phenotypes through genome-wide association studies. Our study demonstrates the utility of iPSCORE for examining how genetic variants influence molecular and physiological traits in iPSCs and derived cell lines.

INTRODUCTION

Due to their ability to differentiate into a variety of cell types, induced pluripotent stem cells (iPSCs) are a potentially powerful model system to study mechanisms underlying non-coding genetic variants associated with human traits, many of which lie in cell-type-specific regulatory regions (Maurano et al., 2012). However, because non-coding regulatory variants can have relatively small effect sizes, hundreds of lines from diverse individuals may be needed to measure genetic associations as opposed to the tens of different lines typically used to study disease-associated coding variants with strong effects (Avior et al., 2016). To enable the study of genetic variants associated with complex diseases and cell-type-specific molecular phenotypes, we and others are establishing large systematically generated collections of iPSCs toward the goal of generating large genomic datasets that will be openly available to researchers (Avior et al., 2016; Kilpinen et al., 2016; McKernan and Watt, 2013; Streeter et al., 2017). Ongoing collections, including large disease-focused iPSC repositories (www.cirm.ca.gov), however, are currently limited in sample diversity and in related individuals (e.g., pedigrees or twins), which would allow for the interrogation of population-associated genetic variation, rare variation, and family-based genetic study designs. Thus, the generation of a resource consisting of hundreds of systematically derived iPSCs with available genomic data including SNP arrays, RNA sequencing (RNA-seq), and whole-genome sequencing, and that includes a variety of familial

This is an open access article under the CC BY license (http://creativecommons.org/licenses/by/4.0/).
CrossMark

architectures and individuals of multiple ethnicities, would further enable a wide variety of study designs to interrogate the genetic basis of phenotype and disease.

There are a number of potential challenges to using iPSC and iPSC-derived cells to model human phenotype and disease. Somatic heterogeneity in iPSC lines that can occur during isolation and culture may interfere with examining genetic variants with subtle effects (Fusaki et al., 2009; International Stem Cell et al., 2011; Nazor et al., 2012). This heterogeneity can include copy-number alterations, which have been reported as occurring in recurrently altered regions in existing collections of pluripotent stem cells (both embryonic stem cells [ESCs] and iPSCs) (International Stem Cell et al., 2011; Laurent et al., 2011; Taapken et al., 2011). However, because many of these lines were not systematically generated and may have undergone prolonged passaging in culture, it is unclear how prevalent these hotspots are in limited passaged lines and/or if other hotspots could be uncovered as additional iPSC are examined. In addition, it is not yet known whether iPSC-derived cell types (cardiomyocytes, neurons, adipocytes) will be useful for functionally examining genetic variants. We and others have recently shown that genetic differences between individuals are associated with a variety of molecular phenotypes in iPSCs, including the transcriptome and epigenome (Burrows et al., 2016; DeBoever et al., 2017; Panopoulos et al., 2017; Rouhani et al., 2014; Thomas et al., 2015), but it is still unclear whether genetic background is associated with molecular phenotypes in iPSC-derived cells.

Here, we describe the iPSCORE (iPSC Collection for Omic Research) resource, a systematically derived and characterized reference panel of iPSC lines. Participants were recruited to include families, twins, and individuals of diverse ethnicity to enable genetic studies investigating the segregation of traits. While the majority of the participants were generally healthy, 39 individuals with heart diseases were included to allow for investigations into heart disease using derived cell types. iPSCs were systematically reprogrammed from fibroblasts and analyzed for pluripotency and the presence and recurrence of somatic copy-number variants (CNVs). We differentiated a subset of iPSCs to cardiomyocytes and examined how the donor's genetic background is associated with gene expression variation in derived cell lines. Finally, we examined and annotated how individuals in the iPSCORE resource carry SNPs associated with diverse genome-wide association studies (GWAS) phenotypes. The iPSCORE resource provides a powerful tool to examine how genetic variants influence molecular and physiological traits across a variety of derived cell types, as well as to functionally interrogate variants underlying a variety of GWAS phenotypes.

RESULTS

Recruitment and Characterization of Individuals in the iPSCORE Resource

We recruited individuals and recorded sex, age, medical history, ethnicity, and relatedness to others in the collection through a questionnaire at enrollment (Figure 1A). Hereafter, we describe the 222 individuals for which we successfully obtained at least one iPSC line (Table S1). There were 124 females ranging in age from 10 to 88 (median age 48), and 98 males ranging in age from 9 to 82 years of age (median age 49) (Figure 1B). The resource includes 143 participants who are members of a family and genetically related to at least one other individual (Figures 1C and S1). In total, there are 41 families that contain between 2 and 14 members, which include seven monozygotic twin pairs and two dizygotic twin pairs (example pedigrees in Figure 1D; see Figure S1 for all pedigrees). Due to the fact that some of the individuals in the 41 families are only related by marriage, there are a total of 136 genetically unrelated individuals in the collection. While most participants in the collection do not have heart disease, there were 25 individuals with arrhythmia (some with multiple types), 13 with cardiomyopathy, and one with structural cardiac malformations (Figure 1E and Table S1A). Using whole-genome sequence data generated from the blood of cardiac disease probands and their families (DeBoever et al., 2017), we examined genetic variation at candidate disease genes and identified four potentially disease-associated variants affecting two families and two singletons (Table S1B). Overall, the iPSCORE resource contains both complex family structures and unrelated individuals across a large spectrum of ages and multiple ethnicities predominantly from healthy donors, but also includes a subset (18%) of individuals that have a diagnosed cardiac disease.

Germline DNA isolated from blood (or in 16 cases from fibroblasts) from each participant was hybridized to the HumanCoreExome BeadChip, and we used the derived genotypes to confirm reported familial relationships, ancestry, and sex. We estimated the proportion of the genome identical-by-descent between each pair of germline samples and observed genetic similarity that was consistent with reported familial relationships, with no cryptically related individuals (Figure 1F). Ethnicities were recorded as free response by the participants (or in a minority of cases, the physician) and categorized into the following "recorded ethnic groups" (number of individuals given): African American (4), Hispanic (15), Asian (30), European (147), Multiple ethnicities reported (18), Indian (6), and Middle Eastern (2). We estimated genetic ancestry by comparing the genetic similarity of the participants to the 1,000 Genomes Project (1KGP) and observed 100% concordance with the reported ethnicity and the most

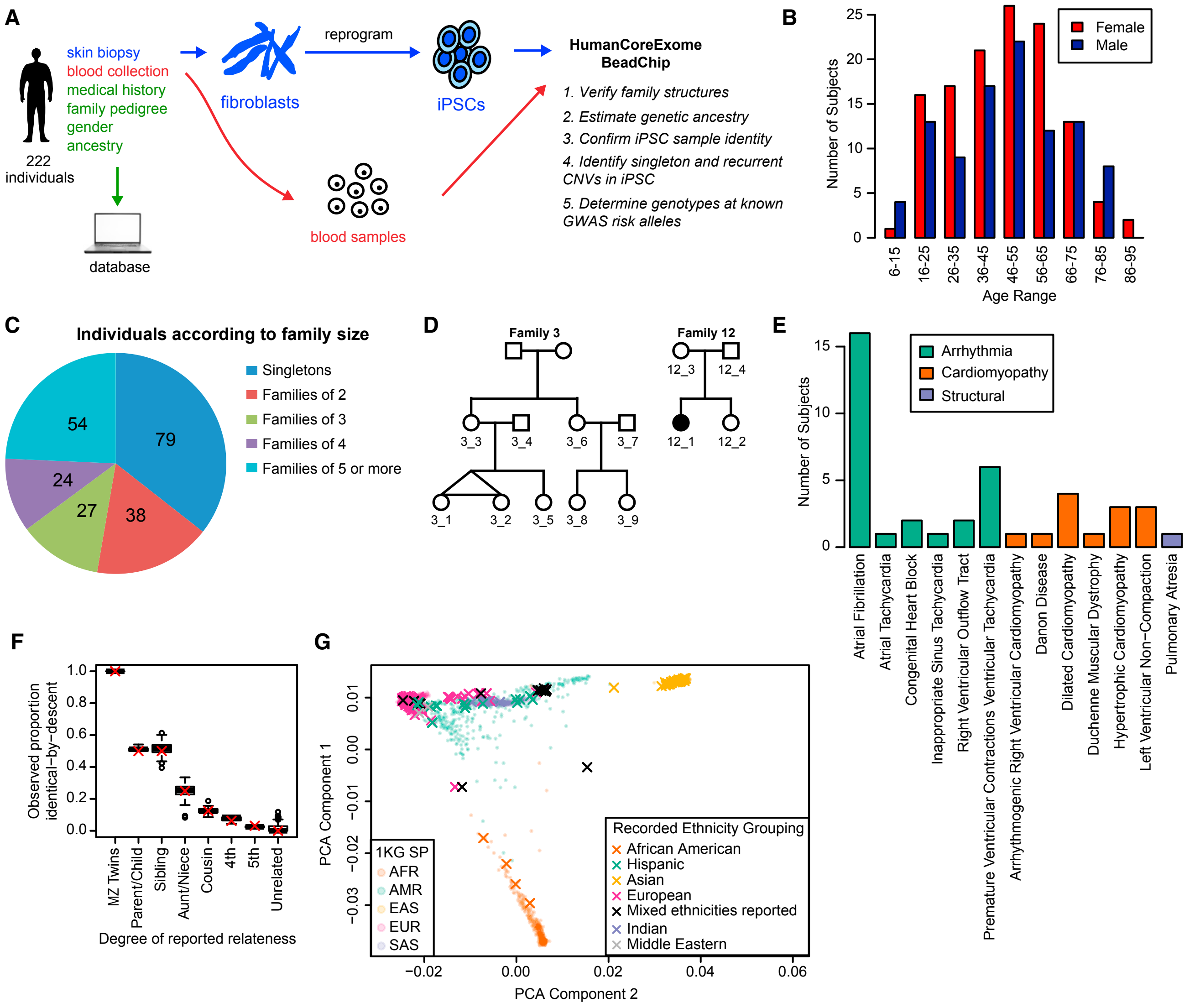

Figure 1. Description of the iPSCORE Cohort

(A) Pipeline for the systematic generation and characterization of 222 iPSC lines. Individuals filled out a questionnaire detailing their medical history, family relationships to other subjects in the cohort, gender, and ancestry. Fibroblasts from skin biopsy were reprogrammed to integration-free iPSC using Sendai virus and frozen at passage 12. Genomic DNA isolated from the iPSC and the subject-matched blood samples were hybridized to the HumanCoreExome array. The resulting data were then used to confirm reported family structure, reported ancestry, and iPSC sample identity (match with blood sample), and to perform CNV analysis (iPSC characterization) and determine status of known disease risk alleles.

(B) Age distributions of males and females.

(C) Pie chart showing how many individuals are singletons or in a family size of 2, 3, 4, and 5 or more.

(D) Pedigrees of two representative families; numbered individuals indicate presence in the study. Family 3 is a two-generation family with identical twins (nine subjects), and Family 12 has a member diagnosed with ventricular tachycardia and congenital heart block (four subjects).

(E) Number of individuals with cardiac disease, grouped by disease type. Some individuals are affected by multiple types of arrhythmia.

(F) Boxplot showing the observed proportion of the genome identical by descent (pIBD) as a function of the reported family relationship. The box hinges indicate the 25th and 75th quantiles and the whiskers extend to 1.5 times the interquartile range. A red "X" indicates the expected mean pIBD given the number of generations that separate the individuals.

(G) An x-y plot showing the first versus second components of a principal component analysis using genotype data from a subset of SNPs present on the array mapped onto a principal component analysis from the 1,000 Genomes Project (1KG) super populations (SP) (small faded circles). Individuals from the iPSCORE cohort are mapped onto these components with their recorded ethnicity grouping shown by a colored X.

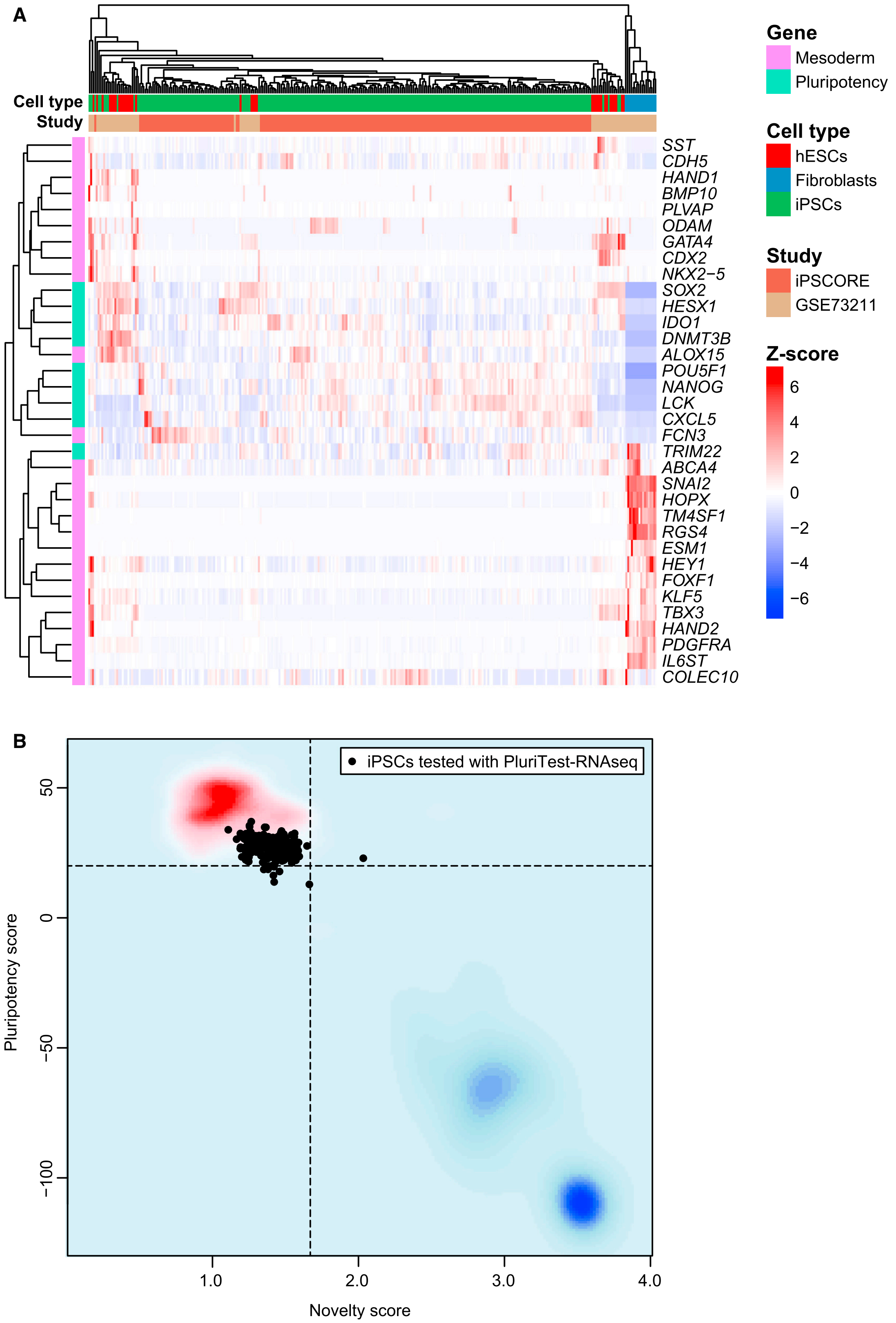

(legend on next page)

similar 1KGP super population using linear discriminant analysis (Figure 1G and Table S1A). However, some heterogeneity was observed in clustering of the first principal components, consistent with some level of unreported admixture. Finally, sex was determined from genotype data and no discrepancies were identified. These results suggest that the samples analyzed are consistent with reported phenotypes and familial relationships.

Generation, Sample Identity Verification, and Pluripotency Testing of iPSC Lines

Skin biopsies collected at enrollment were immediately used to derive fibroblasts for generating iPSCs, while the blood was stored for later DNA extraction (Figure 1A). We used a non-integrative reprogramming method (Sendai virus) to generate the iPSCs and derived multiple clones from each individual (on average three clones), with a minimum of two clones frozen at passage 3 (P3) and at least one clone cultured to later passage (typically P12). We attempted to reprogram fibroblasts from 240 of the recruited participants and obtained iPSCs for 224 individuals, of which 222 passed sample identity quality control (see below).

To confirm sample identity of the iPSC, we hybridized DNA isolated from the iPSC samples (typically at P12) to HumanCoreExome BeadChips and compared it with the genotype data from the matched germline sample. Sample identity was considered confirmed if the iPSC line genetically matched the donor germline sample across 90,099 SNPs. We identified two iPSC lines that did not genetically match the blood sample: for one, we suspect that the blood was mislabeled at time of collection, and for the other, that the iPSC was exchanged with another unknown cell line. In both cases, the anomalous sample did not match with any other sample in the study, and both germline/iPSC pairs were excluded. Overall, 222 of 224 (99.1%) iPSC lines passed sample identity quality control and were included in the study.

To evaluate iPSC pluripotency, we conducted flow cytometry and analyzed gene expression using expression arrays and RNA-seq data. We examined a subset of the lines (50 samples) by flow cytometry, all of which showed >95% positive staining for the pluripotency markers Tra-1-81 and SSEA-4 (Figure S2). For 213 iPSCs with RNA-seq (DeBoever et al., 2017), we compared the expression levels of nine pluripotency (Burridge et al., 2012; Dubois et al., 2011; Vidarsson et al., 2010) and 25 mesoderm markers (Tsankov et al., 2015) to publicly available RNA-seq data from human ESCs (hESCs), iPSCs, and fibroblasts (Choi et al., 2015) (Figure 2A). The iPSCs were comparable with these previously established pluripotent stem cell lines, showing low expression of mesoderm markers and high expression of pluripotency markers (Figure 2A). To further examine iPSC pluripotency, we analyzed the RNA-seq expression data for the 213 lines using PluriTest-RNAseq, a recently modified version of PluriTest (Muller et al., 2011) that has been adapted for RNA-seq (see Supplemental Experimental Procedures; unpublished data by B.M.S., R.W., F.J.M., and J.F.L.) as opposed to gene expression arrays. We observed strong clustering of the iPSCs in the upper left quadrant with 206 of the lines passing the test's criteria (>20 Pluripotency Score, indicating high expression levels of pluripotency-associated gene signatures; <1.67 Novelty, indicating a low probability of epigenetic or genetic abnormalities) (Table S2 and Figure 2B). Of the seven outliers, four have normal karyotypes and three have CNVs that cumulatively account for less than 500 kb in total length per line (see below and Table S3), suggesting that the variation in score is not due to genetic abnormalities. As part of an ongoing project whereby we are differentiating these iPSC lines into cardiomyocytes (see below), we attempted to differentiate four of the outlying samples and successfully differentiated three, which is similar to the overall ~78% success rate (147 of 188 attempted) for first cardiac differentiation attempts, indicating that these outliers show differentiation rates similar to passing lines (data not shown). Thus, these results support that the iPSCORE lines are pluripotent.

Figure 2. Analysis of iPSC Transcriptome Data to Assess Pluripotency

(A) Heatmap and hierarchical clustering showing normalized expression levels (*Z* scores derived from VST expression levels) of nine pluripotency (green) (Burridge et al., 2012; Dubois et al., 2011; Vidarsson et al., 2010) and 25 mesoderm marker genes (pink) (Tsankov et al., 2015) in 213 iPSCORE iPSC lines and 73 cell lines (21 iPSC, 35 hESC, and 17 fibroblast) obtained from GEO: GSE73211 (Choi et al., 2015). Samples are color coded to show whether they are derived from iPSCORE (dark brown) or from GEO: GSE73211 (light brown), and on the basis of tissue type (red for hESC, green for iPSC, and blue for fibroblast). The heatmap shows that iPSCs and hESCs have higher overall expression of pluripotency genes than fibroblasts, which have low expression of pluripotency genes, but higher expression of most mesoderm markers than iPSC lines and hESC lines.

(B) PluriTest-RNAseq-based analysis of 213 iPSCORE lines (green) with RNA-seq data. The red and blue background encodes an empirical density map indicating the location of pluripotent (red) and non-pluripotent (blue) cells in the reference dataset. The x axis represents novelty score, which indicates how much the test iPSC deviates from a normal pluripotent line, with higher values being associated with more somatic characteristics and therefore lower pluripotency. The y axis represents the pluripotency score, a logistic regression model that enables a probability-based choice between pluripotent and non-pluripotent classes (Muller et al., 2011).

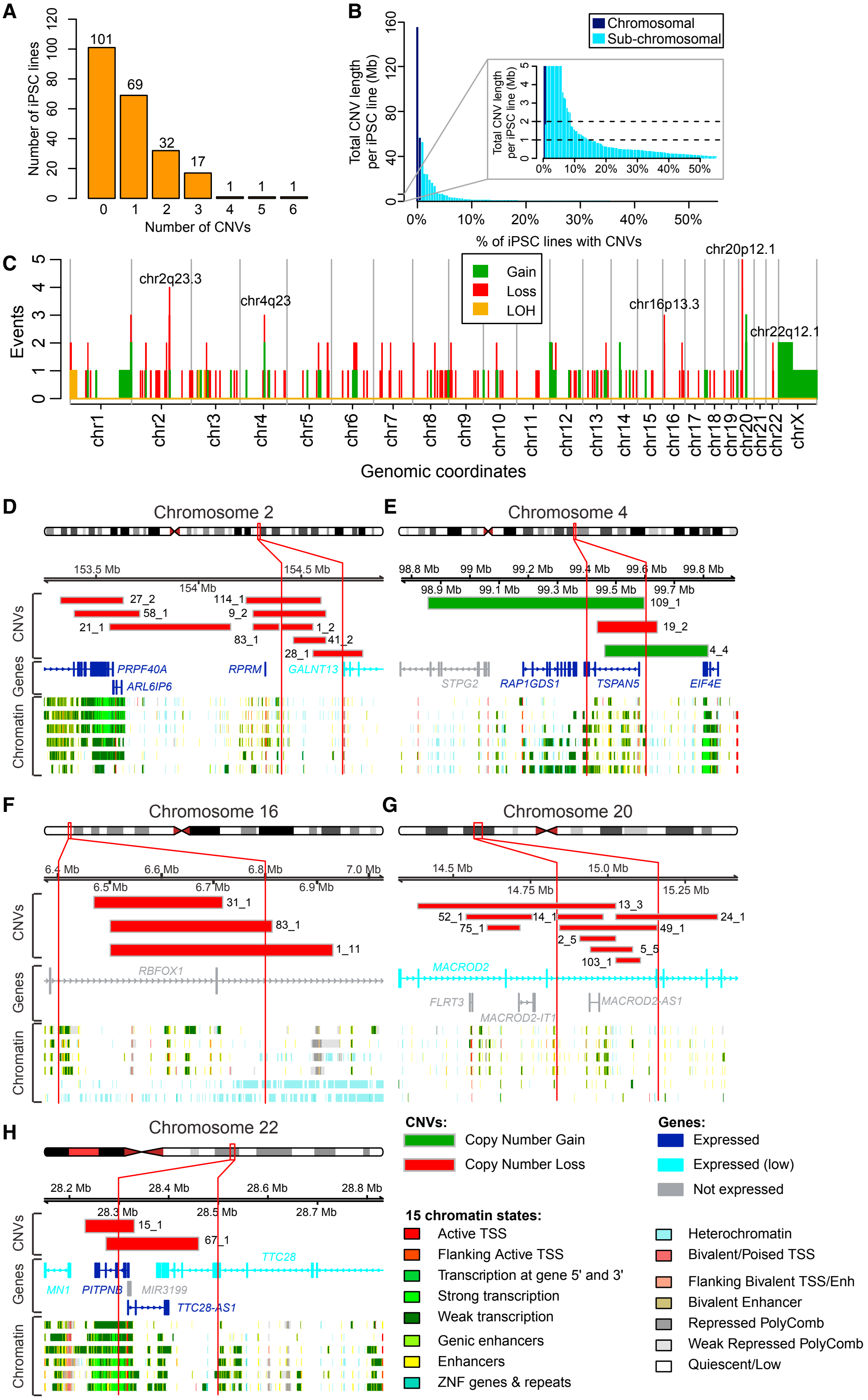

(legend on next page)

Characterization of Somatic Copy-Number Variants

Previous studies have shown that iPSC lines can contain somatic CNVs that were either present in the donor sample or arose during/after the reprogramming process (Abyzov et al., 2012; Hussein et al., 2011, 2013; Young et al., 2012). To examine the genomic integrity of the iPSC lines, we compared the intensity levels and B-allele frequencies of the HumanCoreExome arrays between the matched germline and iPSC DNA samples. We used a visual approach and a paired analysis in Nexus CN, a method that requires iPSC variants to be different from germline, and thus excludes inherited CNVs (see Supplemental Experimental Procedures). We identified 199 regions from 121 cell lines that met our criteria for CNVs with high confidence (listed in Table S3A and Figure S3). Notably, 101 of the 222 iPSC lines (as scored by the criteria described here) have no significant CNVs when compared back with their corresponding germline sample. This is followed by a distribution of iPSCs having between one CNV (69 lines) and six CNVs (1 line) (Figure 3A). We observed one trisomy (chromosome X), one event involving amplification of an entire chromosomal arm (chromosome Xp), and 197 subchromosomal alterations including 151 deletions, 43 amplifications, one loss of heterozygosity, and two allelic imbalances (likely caused by subclonal populations) (Table S3A). Size ranges for subchromosomal alterations ranged from 0.1 Mb to 48.1 Mb (average 1.3 Mb; median 285 kb). For each of the 121 iPSC lines containing one or more CNV, we calculated the cumulative amount (in bp) of CNVs (average 3.8 Mb; median 473 kb) (Figure 3B). A small number of lines carried a disproportionate burden, with 19 lines having more than 2 Mb of CNVs and 33 having more than 1 Mb. Of note, these subchromosomal alterations are almost exclusively outside the detection limits of G-banded karyotyping, which typically cannot detect genomic abnormalities <5 Mb (Manning and Hudgins, 2007; Manning et al., 2010), and therefore these lines would be considered "normal" using a standard method of iPSC characterization. Thus, a majority (~90%) of iPSCORE lines showed no detectable CNVs (101/222, or 45%) or carried CNVs less than 2 Mb (102/222, or 46%).

To investigate whether the somatic CNVs occurred prior to or during/following initial reprogramming (we cannot distinguish between mutations that occurred before or after the cell became an iPSC colony) versus during subsequent iPSC passaging in culture, we selected 17 iPSC lines containing a total of 33 CNVs at P12–P15, and compared their genotypes with a sample of the same line taken at an earlier passage (P3). Only three of the CNVs (9%) were not present at the earlier P3 version of the iPSC line, while 30 (91%) were present (Table S3B). For six of the iPSC lines (containing a total of 11 CNVs), we examined two additional clones at P3, and for one of the lines (containing two CNVs), we examined one additional clone at P3 (Table S3B). Only one of the 13 CNVs examined was present in another clone derived from the same fibroblast culture. These results are in agreement with previous studies that have found most somatic variants (single-nucleotide variants [SNVs] and CNVs) are present at low frequency in the cells of origin and are already present in early passages (Abyzov et al., 2012; Gore et al., 2011; Hussein et al., 2011; Laurent et al., 2011; Mayshar et al., 2010; Ruiz et al., 2013; Young et al., 2012). Our data suggest that systematically generated iPSC lines do not tend to acquire passage-associated CNVs (i.e., when passaged 12–15 times) and that most CNVs are detectable at early stages following iPSC derivation.

Recurrently Altered Chromosomal Regions

To identify genomic regions that may be recurrently altered in iPSCs, we plotted the distribution of the 199 CNVs and then looked for 100-kb-long intervals containing more

Figure 3. Characteristics of the Copy-Number Variants in the 222 iPSC Cell Lines

(A) Histogram showing the number of iPSC cell lines with (N) detected CNV aberrations (x axis); for example, 101 of the iPSC lines have zero aberrations detected.

(B) Histogram showing the cumulative size of CNVs (in megabase pairs) per iPSC cell line as a percentage of the cohort (Total = 222).

(C) Histogram showing the number of alterations in each genomic locus. The five intervals harboring a significant cluster of CNVs are indicated. Gray lines separate chromosomes.

(D–H) Genomic intervals harboring significantly clustered CNVs. The relative chromosomal position of each CNV cluster is shown. Red vertical lines delineate the regions significantly enriched, but additional nearby CNVs are also shown. In each panel, expressed and non-expressed genes are color coded. RNA-seq data of the 213 iPSC lines were used to determine gene expression levels: genes were defined as not expressed (gray) if fewer than 10 iPSC had an expression level of transcripts per million (TPM) >2; genes with a mean TPM <4 were considered as having low expression (light blue); while genes with a mean TPM >= 4 were considered as expressed (dark blue). The rows underneath the genes show 15 chromatin states in one ESC line (ESC.4STAR; top row) and five iPSC lines derived from Roadmap ChromHMM (http://egg2.wustl.edu/roadmap/ [Ernst and Kellis, 2012; Roadmap Epigenomics et al., 2015]). (D) Nine samples (sample ID indicated) harbor deletions (red rectangles) at chr2q23.3, of which five fall in the significantly enriched region. (E) Two samples harbor gains and one harbors a loss at chr4q23. (F) Three samples harbor deletions at chr16p13.3. (G) Nine samples harbor deletions at chr20p12.1, of which seven fall in the associated region. (H) Two samples harbor deletions at chr 22q12.1.

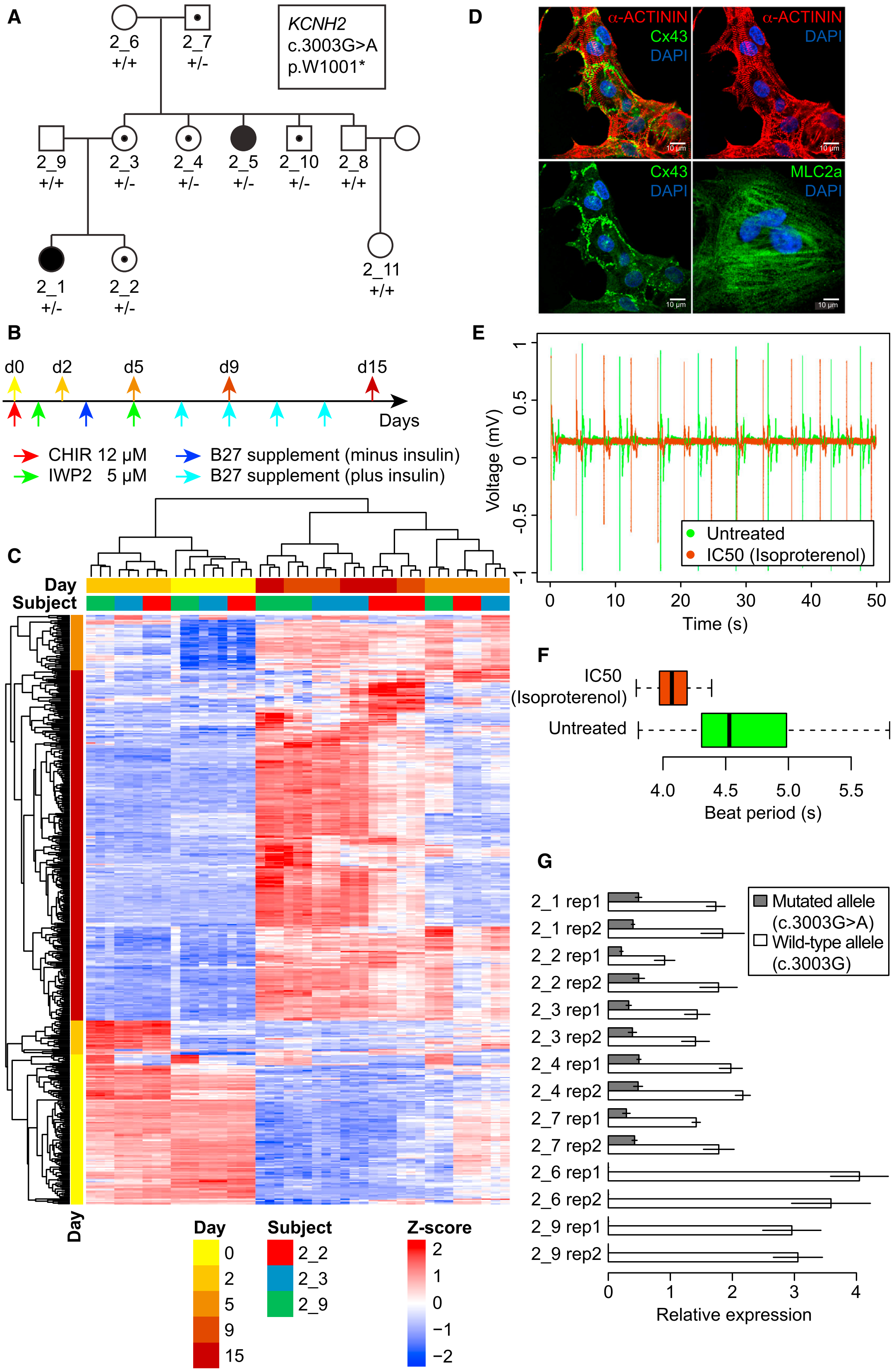

(legend on next page)

CNVs than expected by chance (Figure 3C). We observed five small regions (ranging from 200 to 400 kb) affecting 21 of 222 (9%) iPSC lines where CNVs occurred significantly more often than expected considering a uniform distribution across each individual chromosome (significance testing for aberrant copy number, $p < 0.05$) (Figures 3D–3H; Tables S3A and S3C). The most prevalent recurrent regions occurred on chr2 (chr2q23.3) and chr20 (chr20p12.1), containing an accumulation of five and seven subchromosomal CNVs (all deletions), respectively. The region on chr2 (chr2q23.3) lies in a relatively quiescent interval (not bound by regulatory proteins or modified histones) between two genes: *RPRM* (Reprimo, TP53 Dependent G2 Arrest Mediator Candidate), a tumor-suppressor gene involved in the regulation of p53-dependent cell-cycle arrest (Xu et al., 2012), and thus of potential interest due to the established importance of the p53 pathway in reprogramming (Krizhanovsky and Lowe, 2009); and *GALNT13* (Polypeptide N-Acetylgalactosaminyltransferase 13), a gene expressed at low levels in iPSCs that is involved in the glycosylation of mucins (Hang and Bertozzi, 2005). The chr4 region (chr4q23) overlaps active enhancers and an expressed gene in iPSCs: *TSPAN5* (tetraspanin 5), a member of the transmembrane 4 superfamily involved in the regulation of cell development and growth (Zhou et al., 2014). Although the gene (*RBFOX1*: RNA Binding Protein, Fox-1 Homolog) in the significantly enriched region on chr16 (16p13.3) is not expressed in iPSCs, the interval has previously been shown to be recurrently aberrantly methylated in iPSC lines (Ruiz et al., 2012). The chr20 region (chr20p12.1) affects a relatively quiescent interval and a protein-coding gene expressed at low levels in iPSCs: *MACROD2* (MACRO Domain Containing 2), a gene involved in autism (Jones et al., 2014) and in tamoxifen resistance in breast cancer (Mohseni et al., 2014). The chr22 region (chr22q12.1) affects two protein-coding genes, as well as an antisense RNA, all transcribed in iPSCs: *PITPNB* (Phosphatidylinositol Transfer Protein, Beta), *TTC28* (tetratricopeptide repeat domain 28), and TTC28-AS1 (TTC28 Antisense RNA 1). Although not a statistically significant enrichment in our study due to the relatively high number of CNVs in the iPSC lines on chr20 (resulting in a high background rate for this chromosome), we observed three CNVs overlapping the previously identified chr20q11.2 hotspot region (Laurent et al., 2011) linked with the pluripotency and cell proliferation-associated gene *DNMT3B* (DNA (Cytosine-5-)-Methyltransferase 3 Beta) (Lefort et al., 2008). The fact that the regions significantly enriched for CNVs in our study show other recurrent alterations (aberrant methylation on chr16) or contain actively transcribed genes involved in cell growth and development (chr4, chr22), suggest that these genomic intervals may have functional effects in iPSCs.

iPSC-Derived Cardiomyocytes Can Be Used to Study Molecular and Physiological Traits

To demonstrate the utility of the iPSC lines for studying how genetic variants influence molecular and physiological traits in derived cells, we generated iPSC-derived cardiomyocytes (iPSC-CMs) from individuals in a three-generational family that shows segregation of long-QT syndrome type II (Figure 4A). We differentiated three individuals (2_2, 2_3, and 2_9) in triplicate and profiled them using RNA-seq at five different cardiac differentiation stages

Figure 4. Differentiation of iPSC Lines into Cardiomyocytes and Functional Characterization

(A) Pedigree of the iPSCORE family 2 showing segregation of *KCNH2* mutation (p.W1001*) underlying dominant long-QT syndrome with incomplete penetrance. Individuals with filled in circles display long-QT syndrome, while individuals with black dots are carriers of the mutation.

(B) Protocol used for cardiomyocyte differentiation (Lian et al., 2013). Arrows at the bottom indicate the reagents that were sequentially added to cell culture. Arrows at the top indicate the time points at which cells were collected for whole transcriptome analysis, corresponding to the differentiation stages of pluripotency (day 0 [d0]), mesodermal progenitors (d2), cardiovascular progenitors (d5), committed cardiovascular cells (d9), and cardiomyocytes (d15) (Paige et al., 2012).

(C) Heatmap and hierarchical clustering of expression of the 500 genes with highest variance in expression levels among the 45 time-course samples. Samples (columns) are color coded based on the time point at which they were collected (days 0, 2, 5, 9, and 15) and on the subject from whom they were derived (2_2, 2_3, and 2_9). Genes (rows) are color coded by the four groups (hierarchical clustering), according to the differentiation stage where they were first expressed or most highly expressed (Table S4). Gene expression values are reported *Z* scores of variance stabilized transformed read counts.

(D–F) Analysis of iPSC-derived cardiomyocytes from individual 2_3. (D) Confocal images of iPSC-CMs from sample 2_3 immunostained with sarcomeric α-actinin (ACTN1) (red), Cx43 (green), or MLC2-a (green) at day 34 post differentiation. Cx43 puncta are observed on hiPSC-CM cell membranes especially at cardiomyocyte cell-cell junctions. DAPI was used to counterstain nuclei. MEA analysis: (E) field potential measured from one electrode of one well before and after treatment of iPSC-CMs from sample 2_3 with isoproterenol (IC_{50} 0.01 μM), and (F) boxplot of beat period calculated from the same data.

(G) Real-time qPCR specifically quantifying the transcripts of *KCNH2* with the two genotypes (mutated or wild-type), relative to GAPDH expression (ΔCt) in the iPSC-CMs from seven family members. Expression values are normalized relative to the average of ΔCt.

Error bars represent SDs.

(45 independent samples) (Figure 4B). The five profiled stages were each subsequent to important chemical stimuli in the differentiation process that were previously shown to result in epigenetic changes (Paige et al., 2012): pluripotent (day 0 [d0]), mesodermal progenitors (d2), cardiovascular progenitors (d5), committed cardiovascular cells (d9), and cardiomyocytes (d15). We selected the 500 most variably expressed autosomal genes, divided them into four groups using hierarchical clustering, and annotated them according to the differentiation stage where they were most highly expressed (89 genes expressed at d0, 26 at d2, 41 at d5, and 274 at the combined d9–d15) (Figure 4C). The triplicate samples for each individual at each of the five stages clustered together (the two sets of triplicates at d9 and d15 clustered), suggesting that genetic background correlates with expression differences between the different iPSC lines and derived cardiomyocytes. We performed functional enrichment analysis to confirm that genes in each of the four groups of genes recapitulate important stages of cardiac development. This analysis showed that the genes in group d0 were enriched in gene ontology terms associated with stem cells and processes involved in the specification of cell identity, group d2 genes were involved in mesoderm development and gastrulation, and group d5 genes were associated with embryo and organ development, whereas genes in group d9–d15 were involved in heart muscle development (Table S4). These results are in accordance with the cardiac differentiation stages described by Paige et al. (2012). The iPSC-CMs from sample 2_3 were further interrogated by immunofluorescence for the presence of typical cardiac structural markers, ACTN1, CX43, and MLC2a, and this confirmed that cardiomyocyte-like sarcomeres and gap junctions had developed (Figure 4D). Thus, the iPSCs can be differentiated to cardiomyocytes that show appropriate cardiac morphological structures as well as gene expression patterns that cluster by genetic background.

iPSC-CMs could potentially be used as a model system to assess individual response to drugs through in vitro functional and pharmacological assays. We characterized cardiomyocytes from 2_3 and three additional cell lines (2_1, 13_1, and 14_2) using multielectrode array analysis (MEA), which records extracellular field potentials of clusters or layers of cells and provides measurement of cardiac electrophysiology analogous to an electrocardiogram recording (Figures 4E and S4). All four cell lines displayed cardiomyocyte-like electrophysiological proprieties. When we exposed sample 2_3 to isoproterenol, a β_1 and β_2 adrenoreceptor agonist used for the treatment of bradycardia and heart block, cardiomyocytes showed a significantly increased beat rate (Figures 4E and 4F), consistent with previous reports (Mandel et al., 2012; Scott et al., 2014; Sirenko et al., 2013). Thus, these observations suggest that cardiomyocytes derived from this collection show expected electrophysiological properties in response to drug stimulus, and therefore may allow for studying the genetic components underlying drug response differences between individuals.

The long-QT syndrome that shows segregation in iPSCORE family 2 (Figure 4A; Table S1A) is caused by the p.W1001* mutation in *KCNH2*, which encodes the α subunit of a potassium ion channel essential for the final repolarization of the ventricular action potential (Kupershmidt et al., 2002). It has been proposed that the disease mechanism for this mutation is the reduction of the rapid delayed rectifier current (IKr) due to degradation of the transcript by nonsense-mediated mRNA decay (NMD), and consequent prolongation of the action potential (Gong et al., 2007). To examine this hypothesis, we generated d15 iPSC-derived cardiomyocytes from additional family members (2_1, 2_4, 2_6, and 2_7) and analyzed *KCNH2* expression in all seven individuals by allele-specific qPCR. We found that in the carriers of the mutation, the transcript from the mutated allele was reduced by ~75% (t test $p = 7.4 \times 10^{-7}$) with respect to the wild-type allele, consistent with the proposed NMD hypothesis (Figure 4G). These results demonstrate that iPSC-derived cell types can be used to investigate mechanisms underlying the association of genetic variation with molecular, physiological, and disease phenotypes.

iPSC Lines Carry Genetic Variants Associated with a Variety of Traits and Diseases

Given that many of the iPSC lines in the iPSCORE resource are from healthy donors, they may be useful for examining common genetic variants associated with non-cardiac phenotypes. GWAS have examined hundreds of human phenotypes and identified thousands of SNPs associated with one or more trait (Cingolani et al., 2012). We identified 2,571 of these GWAS SNPs present on the HumanCoreExome arrays that are associated with one or more phenotype and report the risk allele genotypes from the germline samples of the 222 participants (Figure 1A and Table S5). In addition, we examined the distribution of risk/risk, risk/non-risk, and non-risk/non-risk genotypes at these GWAS SNPs and found that for 95% (2,434/2,571), each of the three genotypes was represented, the totals for which can be seen in Figure 5A. These phenotypes include those that are relevant to cardiovascular disease, diabetes, and neurological health, such as QT interval, coronary artery disease, fasting glucose levels, and late-onset Alzheimer's disease (Figures 5B–5E). It has been shown that iPSC lines can be differentiated into a variety of human cell types, including adipocytes (Lian et al., 2016), cardiomyocytes (Burridge et al., 2014), hematopoietic progenitor cells (Ferrell et al., 2015), pancreatic β cells (Tulpule et al., 2013), and several different neuronal

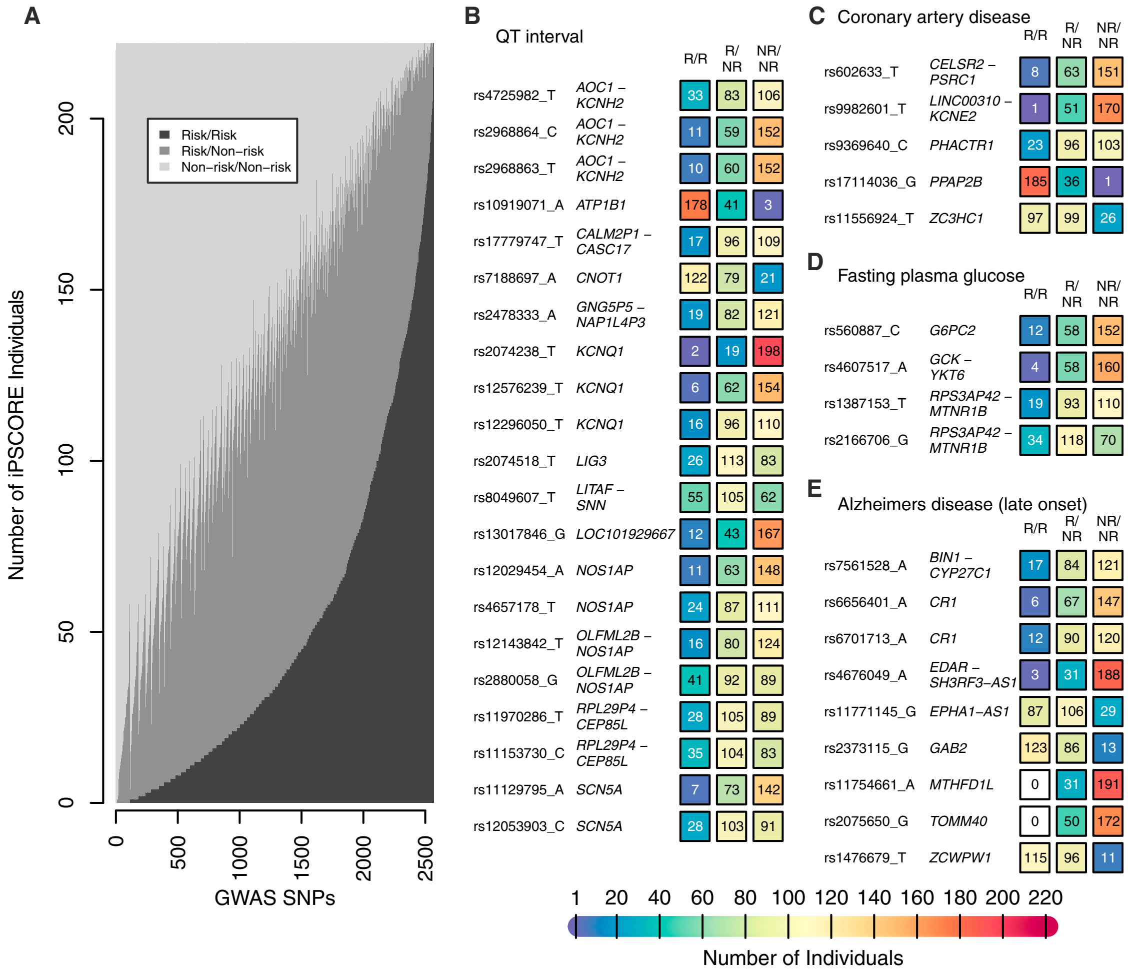

Figure 5. Distributions of GWAS SNP Genotypes in the iPSCORE Resource

(A) Stacked barplot showing the number of individuals in the iPSCORE resource that have particular genotypes at 2,571 SNPs that have been previously associated with one or more phenotypes through GWAS.

(B–E) Counts of individuals that carry the risk/risk (R/R), risk/non-risk (R/NR), and non-risk/non-risk (NR/NR) genotypes for SNPs implicated in the indicated disease. The color of the box indicates the number of individuals on a color scale shown at the bottom. (B) QT interval; (C) coronary artery disease; (D) fasting plasma glucose levels; and (E) Alzheimer's disease (late onset).

See also Table S5.

cell types (Sances et al., 2016). Thus, the iPSC lines in the iPSCORE resource could be used to investigate the molecular mechanisms underlying the genetic risk for a wide variety of traits and diseases in the appropriate derived cell types.

DISCUSSION

Current large-scale collections of iPSCs generally have limited numbers of lines from people of non-European ancestry or individuals in multigenerational families. The iPSCORE collection includes 75 lines from people of Hispanic ethnicity, non-European ancestry, or multiple ancestries, which will aid in studies interrogating population-associated genetic variation or in fine-mapping using trans-ethnicity mapping. Additionally we include multigenerational families and monozygotic twins, which will enable interrogation of rare, family-specific variation, segregation analysis of molecular and physiological traits, and estimation of technical and environmental variation independent of genetic background. The 136 genetically unrelated individuals in the resource enable the derived cell lines to be used for genetic association studies that historically have required unrelated individuals; although with methods that account for sample structure (Kang et al., 2010), these studies can incorporate all 222 individuals. These association studies will be further augmented

by the fact that whole-genome sequence data has been generated from somatic tissue (blood and in some cases fibroblasts) of the iPSCORE participants and is part of the resource (Table S1C). Because risk and non-risk alleles for the vast majority of GWAS SNPs are represented in the genomes of the 222 individuals, this resource will allow for the functional interrogation of these important predominantly regulatory variants in appropriate iPSC-derived cell types. Thus, the nature of the individuals who participated in the iPSCORE resource will allow for diverse experimental approaches to examine how genetic variation affects molecular and physiological traits.

To efficiently characterize more than 200 iPSC lines, we incorporated genomic tools such as the HumanCoreExome BeadChip to examine genomic integrity, establish sample identity, and estimate genetic ancestry and familial relatedness; and RNA-seq to establish pluripotency. Overall, genomic integrity for these low-passage lines was high with almost half of the iPSCs in the iPSCORE resource showing no detectable abnormalities, and ~90% showing less than 2 Mb of cumulative CNV coverage (in bp). It is important to note that genotype array assays are limited to the extent that they are unable to detect balanced chromosomal translocations or abnormalities occurring at a frequency lower than 20% (D'Antonio et al., 2017 [this issue of *Stem Cell Reports*]); however, previous studies using genotype arrays have found higher ratios and frequencies of abnormalities in iPSCs (International Stem Cell et al., 2011; Laurent et al., 2011; Taapken et al., 2011) than we report, suggesting that a systematic approach to iPSC generation can result in significantly fewer abnormalities. We also used RNA-seq data to validate the quality of the iPSCORE lines by comparing them with publicly available RNA-seq data for stem cells previously shown to be pluripotent and performed pluripotency estimation using PluriTest-RNAseq. Thus, the adoption of high-throughput genomic methods can help reduce costs and enable effective and relatively rapid characterization of iPSC lines for genomic integrity and pluripotency.

Although we observed low overall rates of CNVs, we observed five recurrently altered regions. Three of the intervals are quiescent, containing few (if any) regulatory elements and either low or unexpressed genes. However, one of these intervals (the chr16 interval) is recurrently aberrantly methylated in iPSC lines (Ruiz et al., 2012), which suggests that the region has functional significance in iPSCs. The other two recurrently altered intervals contain actively transcribed genes involved in cell growth and development in iPSCs. Further studies are needed to determine whether these significantly altered intervals offered a selective advantage in the reprogramming process or were due to hotspots that recurrently mutate at a low rate (2%–4%) in iPSCs (or the parental cells). Previous studies have shown that most somatic variants (both CNVs and SNVs) observed in iPSCs are already present in the cell of origin (Abyzov et al., 2012; Cheng et al., 2012; Gore et al., 2011; Ruiz et al., 2013; Young et al., 2012). We observed in a small number of lines that the majority of somatic CNVs observed in later-passage iPSCs (P12) were already present at earlier passages (P3), supporting the model that most somatic variants are likely derived from the parental cell. In total, these data suggest that while a significant number of our systematically generated iPSCs examined at relatively early passage (P12) do not harbor detectable genomic alterations, some iPSCs showed recurrently altered genomic intervals that may reveal a selective advantage during the reprogramming process, and that many of these may be present in the cell of origin.

In summary, iPSCORE is a high-quality large-scale collection of iPSCs from 222 individuals that is currently publicly available through the NHLBI-contracted biorepository at WiCell Research Institute, with phenotype and genomic data (SNP arrays, RNA-seq, whole-genome sequencing) being released through public databases. We are currently using the resource to differentiate the iPSC lines into cardiomyocytes with the intention of investigating molecular (ATAC-seq [assay for transposase-accessible chromatin with high-throughput sequencing], DNA methylation, H3K27ac marks) and physiological phenotypes in both iPSC lines and iPSC-derived cardiomyocytes. As these, and other genomic (such as whole-genome sequences) and molecular data for a variety of derived cell types become available, the resource will become substantially richer over time, enabling the research community to efficiently address a multitude of questions regarding human biology and disease.

EXPERIMENTAL PROCEDURES

Enrollment of Subjects for the iPSCORE Resource

This resource was established as part of the Next Generation Consortium of the National Heart, Lung and Blood Institute and is available to researchers through the biorepository at WiCell Research Institute (www.wicell.org; NHLBI Next Gen Collection). For-profit organizations can contact the corresponding author directly to discuss line availability. Healthy individuals were recruited for the resource through both the Twin Sibling Pedigree cohort (TSP; a population-based twin registry spanning counties in Southern California) (Pasha et al., 2013) and open enrollment through the Clinical and Translational Research Institute (CTRI) at the University of California at San Diego (UCSD). Thirty-nine patients at UCSD Sulpizio Cardiovascular Center were also recruited. These collections were approved by the Institutional Review Boards of the UCSD and The Salk Institute (Project no. 110776ZF). Each of the subjects first consented to the study and filled out a questionnaire. These data were transcribed to a database and subjects were de-identified with a new sample ID (Table S1A).

Family relatedness was also recorded in the questionnaire and converted into pedigree diagrams using family tree drawing software (Madeline 2.0, University of Michigan) (Figure S1). Ethnicity was reported as a free-response answer and translated into one of six recorded ethnicity groupings (African American, European, Hispanic, Indian, Middle Eastern, Asian) (Table S1A). A seventh category was used when more than one ethnicity was reported; that individual was recorded as "Multiple ethnicities reported." For 12 individuals, the race/ethnicity question was reported by a treating physician (Table S1A, denoted by an asterisk in column K). Finally, a blood sample (for germline DNA) was collected and a skin biopsy was performed to generate fibroblast stocks for iPSC reprogramming.

ACCESSION NUMBERS

Phenotype, array genotype, RNA-seq expression values, and whole-genome sequence genotype data are available through dbGaP: phs000924 and phs001325. The 222 iPSC lines are available through WiCell Research Institute (www.wicell.org; NHLBI Next Gen Collection). Note: The informed consent for the individuals in the iPSCORE resource included the allowance for commercial use. However, licensing agreements are still required between individual commercial entities and DNAVEC (maker of SeV) and Wicell (iPSC bank).

SUPPLEMENTAL INFORMATION

Supplemental Information includes Supplemental Experimental Procedures, five figures, and five tables and can be found with this article online at http://dx.doi.org/10.1016/j.stemcr.2017.03.012.

AUTHOR CONTRIBUTIONS

K.A.F., O.H., and A.D.P. conceived the study. A.D.A., M.G., J.D.G., F.D., K.E.D., Y.H., V.M., A.D.P., J.O., C.T.D., R.F., P.B., K.F., M.C., V.B., C.A.M., and A.D.-C. generated fibroblasts, iPSCs, and iPSC-derived cardiomyocytes. C.Z. performed flow cytometry. S.I.H. and A.A. performed imaging. P.B. performed qRT-PCR. M.D., P.B., C.D., H.M., J.R., R.W., D.A.J., M.K.R.D., W.W.G., H.L., N.N., B.M.S., F.-J.M., and E.N.S. performed data processing and computational analyses. K.J., B.C.N., T.J.M., F.R., D.T.O., E.A., and N.C.C. participated in recruitment and enrollment of individuals into study. K.A.F., J.C.I.B., N.C.C., G.W.Y., S.M.E., L.S.B.G., W.T.B., J.F.L., E.N.S., and A.D.P. oversaw and managed the study. K.A.F., M.D., P.B., E.N.S., and A.D.P. prepared the manuscript.

ACKNOWLEDGMENTS

This work was supported in part by a California Institute for Regenerative Medicine (CIRM) grant GC1R-06673 (to K.A.F.), TR3-05687 (to E.A.), and NIH grants HG008118 (to K.A.F.), HL107442 (to K.A.F., S.M.E., G.W.Y., N.C.C., and J.C.I.B.), DK105541 (to K.A.F.) and DK112155 (to K.A.F.); The following individuals were partially supported by NIH Supplements: A.D.A. (HL107442), M.G. (HG008118), and V.B. (EY021237). C.Z. was supported by CIRM Bridges TBI-01186 grant and K.J.F. and M.C. by CIRM Bridges II EDUC2-08375 grant awarded to California State University San Marcos (to Bianca R. Mothé). The Leona M. and Harry B. Helmsley Charitable Trust (2012-PG-MED002) (J.C.I.B.), Universidad Catolica San Antonio de Murcia (UCAM) and the G. Harold and Leila Y. Mathers Charitable Foundation (J.C.I.B.), the NIH grant UL1TR000100 of CTSA funding prior to August 13, 2015 and grant UL1TR001442 of CTSA funding beginning August 13, 2015 and beyond; J.D.G. and C.D. are supported in part by an institutional award to the UCSD Genetics Training Program from the NIGMS (T32GM008666); C.D. is supported in part by CIRM Interdisciplinary Stem Cell Training Program at UCSD II (TG2-01154); D.A.J. and M.K.R.D. are supported in part by NIH National Library of Medicine Training Grant (4T15LM011271). W.W.G. is supported in part by U.S. Department of Health and Human Services training grant (5T32GM008806-15). P.B. is supported in part by the Swiss National Science Foundation (P2LAP3-155105 and P300PA-167612); HumanCoreExome array data were generated at the UCSD IGM Genomics Center with support from NIH grant P30CA023100. R.M.W. and J.F.L. were supported by CIRM Award for Tools and Technologies (RT3-07655). F.-J.M. and B.M.S. were supported by grants from the BMBF (13GW0128A and 01GM1513D) and from the Deutsche Forschungsgemeinschaft (German Research Foundation DFG MU 3231/3-1). We thank the Gallagher Family for their generous gift to the University of Notre Dame to support stem cell research. We thank Mahdieh Khosroheidari for processing of array data; Navinder Sawhney, Denise Bernard, Gregory Feld, David Krummen, Ulrika Green, and Paul Grossfeld for assistance with recruitment of individuals. We dedicate this work to the memory of our dear colleague Dr. Daniel O'Connor.

Received: July 27, 2016
Revised: March 8, 2017
Accepted: March 13, 2017
Published: April 6, 2017

REFERENCES

Abyzov, A., Mariani, J., Palejev, D., Zhang, Y., Haney, M.S., Tomasini, L., Ferrandino, A.F., Rosenberg Belmaker, L.A., Szekely, A., Wilson, M., et al. (2012). Somatic copy number mosaicism in human skin revealed by induced pluripotent stem cells. Nature *492*, 438–442.

Avior, Y., Sagi, I., and Benvenisty, N. (2016). Pluripotent stem cells in disease modelling and drug discovery. Nat. Rev. Mol. Cell Biol. *17*, 170–182.

Burridge, P.W., Keller, G., Gold, J.D., and Wu, J.C. (2012). Production of de novo cardiomyocytes: human pluripotent stem cell differentiation and direct reprogramming. Cell Stem Cell *10*, 16–28.

Burridge, P.W., Matsa, E., Shukla, P., Lin, Z.C., Churko, J.M., Ebert, A.D., Lan, F., Diecke, S., Huber, B., Mordwinkin, N.M., et al. (2014). Chemically defined generation of human cardiomyocytes. Nat. Methods *11*, 855–860.

Burrows, C.K., Banovich, N.E., Pavlovic, B.J., Patterson, K., Gallego Romero, I., Pritchard, J.K., and Gilad, Y. (2016). Genetic variation, not cell type of origin, underlies the majority of identifiable regulatory differences in iPSCs. PLoS Genet. *12*, e1005793.

Cheng, L., Hansen, N.F., Zhao, L., Du, Y., Zou, C., Donovan, F.X., Chou, B.K., Zhou, G., Li, S., Dowey, S.N., et al. (2012). Low

incidence of DNA sequence variation in human induced pluripotent stem cells generated by nonintegrating plasmid expression. Cell Stem Cell *10*, 337–344.

Choi, J., Lee, S., Mallard, W., Clement, K., Tagliazucchi, G.M., Lim, H., Choi, I.Y., Ferrari, F., Tsankov, A.M., Pop, R., et al. (2015). A comparison of genetically matched cell lines reveals the equivalence of human iPSCs and ESCs. Nat. Biotechnol. *33*, 1173–1181.

Cingolani, P., Platts, A., Wang le, L., Coon, M., Nguyen, T., Wang, L., Land, S.J., Lu, X., and Ruden, D.M. (2012). A program for annotating and predicting the effects of single nucleotide polymorphisms, SnpEff: SNPs in the genome of Drosophila melanogaster strain w1118; iso-2; iso-3. Fly *6*, 80–92.

D'Antonio, M., Woodruff, G., Nathanson, J.L., D'Antonio-Chronowska, A., Arias, A., Matsui, H., Williams, R., Herrera, C., Reyna, S.M., Yeo, G.W., et al. (2017). High-throughput and cost-effective characterization of induced pluripotent stem cells. Stem Cell Reports *8*, this issue, 1101–1111.

DeBoever, C., Li, H., Jakubosky, D., Benaglio, P., Reyna, J., Olson, K.M., Huang, H., Biggs, W., Sandoval, E., D'Antonio, M., et al. (2017). Large-scale profiling reveals the influence of genetic variation on gene expression in human induced pluripotent stem cells. Cell Stem Cell. Published online April 6, 2017. http://dx.doi.org/10.1016/j.stem.2017.03.009.

Dubois, N.C., Craft, A.M., Sharma, P., Elliott, D.A., Stanley, E.G., Elefanty, A.G., Gramolini, A., and Keller, G. (2011). SIRPA is a specific cell-surface marker for isolating cardiomyocytes derived from human pluripotent stem cells. Nat. Biotechnol. *29*, 1011–1018.

Ernst, J., and Kellis, M. (2012). ChromHMM: automating chromatin-state discovery and characterization. Nat. Methods *9*, 215–216.

Ferrell, P.I., Xi, J., Ma, C., Adlakha, M., and Kaufman, D.S. (2015). The RUNX1 +24 enhancer and P1 promoter identify a unique subpopulation of hematopoietic progenitor cells derived from human pluripotent stem cells. Stem Cells *33*, 1130–1141.

Fusaki, N., Ban, H., Nishiyama, A., Saeki, K., and Hasegawa, M. (2009). Efficient induction of transgene-free human pluripotent stem cells using a vector based on Sendai virus, an RNA virus that does not integrate into the host genome. Proc. Jpn. Acad. Ser. B Phys. Biol. Sci. *85*, 348–362.

Gong, Q., Zhang, L., Vincent, G.M., Horne, B.D., and Zhou, Z. (2007). Nonsense mutations in hERG cause a decrease in mutant mRNA transcripts by nonsense-mediated mRNA decay in human long-QT syndrome. Circulation *116*, 17–24.

Gore, A., Li, Z., Fung, H.L., Young, J.E., Agarwal, S., Antosiewicz-Bourget, J., Canto, I., Giorgetti, A., Israel, M.A., Kiskinis, E., et al. (2011). Somatic coding mutations in human induced pluripotent stem cells. Nature *471*, 63–67.

Hang, H.C., and Bertozzi, C.R. (2005). The chemistry and biology of mucin-type O-linked glycosylation. Bioorg. Med. Chem. *13*, 5021–5034.

Hussein, S.M., Batada, N.N., Vuoristo, S., Ching, R.W., Autio, R., Narva, E., Ng, S., Sourour, M., Hamalainen, R., Olsson, C., et al. (2011). Copy number variation and selection during reprogramming to pluripotency. Nature *471*, 58–62.

Hussein, S.M., Elbaz, J., and Nagy, A.A. (2013). Genome damage in induced pluripotent stem cells: assessing the mechanisms and their consequences. Bioessays *35*, 152–162.

International Stem Cell Initiative, Amps, K., Andrews, P.W., Anyfantis, G., Armstrong, L., Avery, S., Baharvand, H., Baker, J., Baker, D., Munoz, M.B., et al. (2011). Screening ethnically diverse human embryonic stem cells identifies a chromosome 20 minimal amplicon conferring growth advantage. Nat. Biotechnol. *29*, 1132–1144.

Jones, R.M., Cadby, G., Blangero, J., Abraham, L.J., Whitehouse, A.J., and Moses, E.K. (2014). MACROD2 gene associated with autistic-like traits in a general population sample. Psychiatr. Genet. *24*, 241–248.

Kang, H.M., Sul, J.H., Service, S.K., Zaitlen, N.A., Kong, S.Y., Freimer, N.B., Sabatti, C., and Eskin, E. (2010). Variance component model to account for sample structure in genome-wide association studies. Nat. Genet. *42*, 348–354.

Kilpinen, H., Goncalves, A., Leha, A., Afzal, V., Ashford, S., Bala, S., Bensaddek, D., Casale, F.P., Culley, O., Danacek, P., et al. (2016). Common genetic variation drives molecular heterogeneity in human iPSCs. bioRxiv http://dx.doi.org/10.1101/055160.

Krizhanovsky, V., and Lowe, S.W. (2009). Stem cells: the promises and perils of p53. Nature *460*, 1085–1086.

Kupershmidt, S., Yang, T., Chanthaphaychith, S., Wang, Z., Towbin, J.A., and Roden, D.M. (2002). Defective human Ether-a-go-go-related gene trafficking linked to an endoplasmic reticulum retention signal in the C terminus. J. Biol. Chem. *277*, 27442–27448.

Laurent, L.C., Ulitsky, I., Slavin, I., Tran, H., Schork, A., Morey, R., Lynch, C., Harness, J.V., Lee, S., Barrero, M.J., et al. (2011). Dynamic changes in the copy number of pluripotency and cell proliferation genes in human ESCs and iPSCs during reprogramming and time in culture. Cell Stem Cell *8*, 106–118.

Lefort, N., Feyeux, M., Bas, C., Feraud, O., Bennaceur-Griscelli, A., Tachdjian, G., Peschanski, M., and Perrier, A.L. (2008). Human embryonic stem cells reveal recurrent genomic instability at 20q11.21. Nat. Biotechnol. *26*, 1364–1366.

Lian, X., Zhang, J., Azarin, S.M., Zhu, K., Hazeltine, L.B., Bao, X., Hsiao, C., Kamp, T.J., and Palecek, S.P. (2013). Directed cardiomyocyte differentiation from human pluripotent stem cells by modulating Wnt/beta-catenin signaling under fully defined conditions. Nat. Protoc. *8*, 162–175.

Lian, Q., Zhang, Y., Liang, X., Gao, F., and Tse, H.F. (2016). Directed differentiation of human-induced pluripotent stem cells to mesenchymal stem cells. Methods Mol. Biol. *1416*, 289–298.

Mandel, Y., Weissman, A., Schick, R., Barad, L., Novak, A., Meiry, G., Goldberg, S., Lorber, A., Rosen, M.R., Itskovitz-Eldor, J., et al. (2012). Human embryonic and induced pluripotent stem cell-derived cardiomyocytes exhibit beat rate variability and power-law behavior. Circulation *125*, 883–893.

Manning, M., and Hudgins, L. (2007). Use of array-based technology in the practice of medical genetics. Genet. Med. *9*, 650–653.

Manning, M., Hudgins, L., Professional, P., and Guidelines, C. (2010). Array-based technology and recommendations for utilization in medical genetics practice for detection of chromosomal abnormalities. Genet. Med. *12*, 742–745.

Maurano, M.T., Humbert, R., Rynes, E., Thurman, R.E., Haugen, E., Wang, H., Reynolds, A.P., Sandstrom, R., Qu, H., Brody, J., et al. (2012). Systematic localization of common disease-associated variation in regulatory DNA. Science *337*, 1190–1195.

Mayshar, Y., Ben-David, U., Lavon, N., Biancotti, J.C., Yakir, B., Clark, A.T., Plath, K., Lowry, W.E., and Benvenisty, N. (2010). Identification and classification of chromosomal aberrations in human induced pluripotent stem cells. Cell Stem Cell *7*, 521–531.

McKernan, R., and Watt, F.M. (2013). What is the point of large-scale collections of human induced pluripotent stem cells? Nat. Biotechnol. *31*, 875–877.

Mohseni, M., Cidado, J., Croessmann, S., Cravero, K., Cimino-Mathews, A., Wong, H.Y., Scharpf, R., Zabransky, D.J., Abukhdeir, A.M., Garay, J.P., et al. (2014). MACROD2 overexpression mediates estrogen independent growth and tamoxifen resistance in breast cancers. Proc. Natl. Acad. Sci. USA *111*, 17606–17611.

Muller, F.J., Schuldt, B.M., Williams, R., Mason, D., Altun, G., Papapetrou, E.P., Danner, S., Goldmann, J.E., Herbst, A., Schmidt, N.O., et al. (2011). A bioinformatic assay for pluripotency in human cells. Nat. Methods *8*, 315–317.

Nazor, K.L., Altun, G., Lynch, C., Tran, H., Harness, J.V., Slavin, I., Garitaonandia, I., Muller, F.J., Wang, Y.C., Boscolo, F.S., et al. (2012). Recurrent variations in DNA methylation in human pluripotent stem cells and their differentiated derivatives. Cell Stem Cell *10*, 620–634.

Paige, S.L., Thomas, S., Stoick-Cooper, C.L., Wang, H., Maves, L., Sandstrom, R., Pabon, L., Reinecke, H., Pratt, G., Keller, G., et al. (2012). A temporal chromatin signature in human embryonic stem cells identifies regulators of cardiac development. Cell *151*, 221–232.

Panopoulos, A.D., Smith, E.N., Arias, A.D., Shepard, P.J., Hishida, Y., Modesto, V., Diffenderfer, K.E., Conner, C., Biggs, W., Sandoval, E., et al. (2017). Aberrant DNA methylation in human iPSCs associates with MYC binding motifs in a clone-specific manner independent of genetics. Cell Stem Cell. Published online April 6, 2017. http://dx.doi.org/10.1016/j.stem.2017.03.010.

Pasha, D.N., Davis, J.T., Rao, F., Chen, Y., Wen, G., Fung, M.M., Mahata, M., Zhang, K., Trzebinska, D., Mustapic, M., et al. (2013). Heritable influence of DBH on adrenergic and renal function: twin and disease studies. PLoS One *8*, e82956.

Roadmap Epigenomics, C., Kundaje, A., Meuleman, W., Ernst, J., Bilenky, M., Yen, A., Heravi-Moussavi, A., Kheradpour, P., Zhang, Z., Wang, J., et al. (2015). Integrative analysis of 111 reference human epigenomes. Nature *518*, 317–330.

Rouhani, F., Kumasaka, N., de Brito, M.C., Bradley, A., Vallier, L., and Gaffney, D. (2014). Genetic background drives transcriptional variation in human induced pluripotent stem cells. PLoS Genet. *10*, e1004432.

Ruiz, S., Diep, D., Gore, A., Panopoulos, A.D., Montserrat, N., Plongthongkum, N., Kumar, S., Fung, H.L., Giorgetti, A., Bilic, J., et al. (2012). Identification of a specific reprogramming-associated epigenetic signature in human induced pluripotent stem cells. Proc. Natl. Acad. Sci. USA *109*, 16196–16201.

Ruiz, S., Gore, A., Li, Z., Panopoulos, A.D., Montserrat, N., Fung, H.L., Giorgetti, A., Bilic, J., Batchelder, E.M., Zaehres, H., et al. (2013). Analysis of protein-coding mutations in hiPSCs and their possible role during somatic cell reprogramming. Nat. Commun. *4*, 1382.

Sances, S., Bruijn, L.I., Chandran, S., Eggan, K., Ho, R., Klim, J.R., Livesey, M.R., Lowry, E., Macklis, J.D., Rushton, D., et al. (2016). Modeling ALS with motor neurons derived from human induced pluripotent stem cells. Nat. Neurosci. *16*, 542–553.

Scott, C.W., Zhang, X., Abi-Gerges, N., Lamore, S.D., Abassi, Y.A., and Peters, M.F. (2014). An impedance-based cellular assay using human iPSC-derived cardiomyocytes to quantify modulators of cardiac contractility. Toxicol. Sci. *142*, 331–338.

Sirenko, O., Crittenden, C., Callamaras, N., Hesley, J., Chen, Y.W., Funes, C., Rusyn, I., Anson, B., and Cromwell, E.F. (2013). Multiparameter in vitro assessment of compound effects on cardiomyocyte physiology using iPSC cells. J. Biomol. Screen. *18*, 39–53.

Streeter, I., Harrison, P.W., Faulconbridge, A., The HipSci, Consortium, Flicek, P., Parkinson, H., and Clarke, L. (2017). The human-induced pluripotent stem cell initiative-data resources for cellular genetics. Nucleic Acids Res. *45*, D691–D697.

Taapken, S.M., Nisler, B.S., Newton, M.A., Sampsell-Barron, T.L., Leonhard, K.A., McIntire, E.M., and Montgomery, K.D. (2011). Karotypic abnormalities in human induced pluripotent stem cells and embryonic stem cells. Nat. Biotechnol. *29*, 313–314.

Thomas, S.M., Kagan, C., Pavlovic, B.J., Burnett, J., Patterson, K., Pritchard, J.K., and Gilad, Y. (2015). Reprogramming LCLs to iPSCs results in recovery of donor-specific gene expression signature. PLoS Genet. *11*, e1005216.

Tsankov, A.M., Akopian, V., Pop, R., Chetty, S., Gifford, C.A., Daheron, L., Tsankova, N.M., and Meissner, A. (2015). A qPCR ScoreCard quantifies the differentiation potential of human pluripotent stem cells. Nat. Biotechnol. *33*, 1182–1192.

Tulpule, A., Kelley, J.M., Lensch, M.W., McPherson, J., Park, I.H., Hartung, O., Nakamura, T., Schlaeger, T.M., Shimamura, A., and Daley, G.Q. (2013). Pluripotent stem cell models of Shwachman-Diamond syndrome reveal a common mechanism for pancreatic and hematopoietic dysfunction. Cell Stem Cell *12*, 727–736.

Vidarsson, H., Hyllner, J., and Sartipy, P. (2010). Differentiation of human embryonic stem cells to cardiomyocytes for in vitro and in vivo applications. Stem Cell Rev. *6*, 108–120.

Xu, M., Knox, A.J., Michaelis, K.A., Kiseljak-Vassiliades, K., Kleinschmidt-DeMasters, B.K., Lillehei, K.O., and Wierman, M.E. (2012). Reprimo (RPRM) is a novel tumor suppressor in pituitary tumors and regulates survival, proliferation, and tumorigenicity. Endocrinology *153*, 2963–2973.

Young, M.A., Larson, D.E., Sun, C.W., George, D.R., Ding, L., Miller, C.A., Lin, L., Pawlik, K.M., Chen, K., Fan, X., et al. (2012). Background mutations in parental cells account for most of the genetic heterogeneity of induced pluripotent stem cells. Cell Stem Cell *10*, 570–582.

Zhou, J., Fujiwara, T., Ye, S., Li, X., and Zhao, H. (2014). Downregulation of Notch modulators, tetraspanin 5 and 10, inhibits osteoclastogenesis in vitro. Calcif. Tissue Int. *95*, 209–217.

Stem Cell Reports, Volume *8*

Supplemental Information

iPSCORE: A Resource of 222 iPSC Lines Enabling Functional Characterization of Genetic Variation across a Variety of Cell Types

Athanasia D. Panopoulos, Matteo D'Antonio, Paola Benaglio, Roy Williams, Sherin I. Hashem, Bernhard M. Schuldt, Christopher DeBoever, Angelo D. Arias, Melvin Garcia, Bradley C. Nelson, Olivier Harismendy, David A. Jakubosky, Margaret K.R. Donovan, William W. Greenwald, KathyJean Farnam, Megan Cook, Victor Borja, Carl A. Miller, Jonathan D. Grinstein, Frauke Drees, Jonathan Okubo, Kenneth E. Diffenderfer, Yuriko Hishida, Veronica Modesto, Carl T. Dargitz, Rachel Feiring, Chang Zhao, Aitor Aguirre, Thomas J. McGarry, Hiroko Matsui, He Li, Joaquin Reyna, Fangwen Rao, Daniel T. O'Connor, Gene W. Yeo, Sylvia M. Evans, Neil C. Chi, Kristen Jepsen, Naoki Nariai, Franz-Josef Müller, Lawrence S.B. Goldstein, Juan Carlos Izpisua Belmonte, Eric Adler, Jeanne F. Loring, W. Travis Berggren, Agnieszka D'Antonio-Chronowska, Erin N. Smith, and Kelly A. Frazer

Supplemental Information includes Figures S1-S5, Tables S1-S5 and Supplemental Experimental Procedures.

SUPPLEMENTARY FIGURES

Figure S1. Structures of the 41 families in the iPSCORE resource. Related to Figures 1A, 1C and 1D.

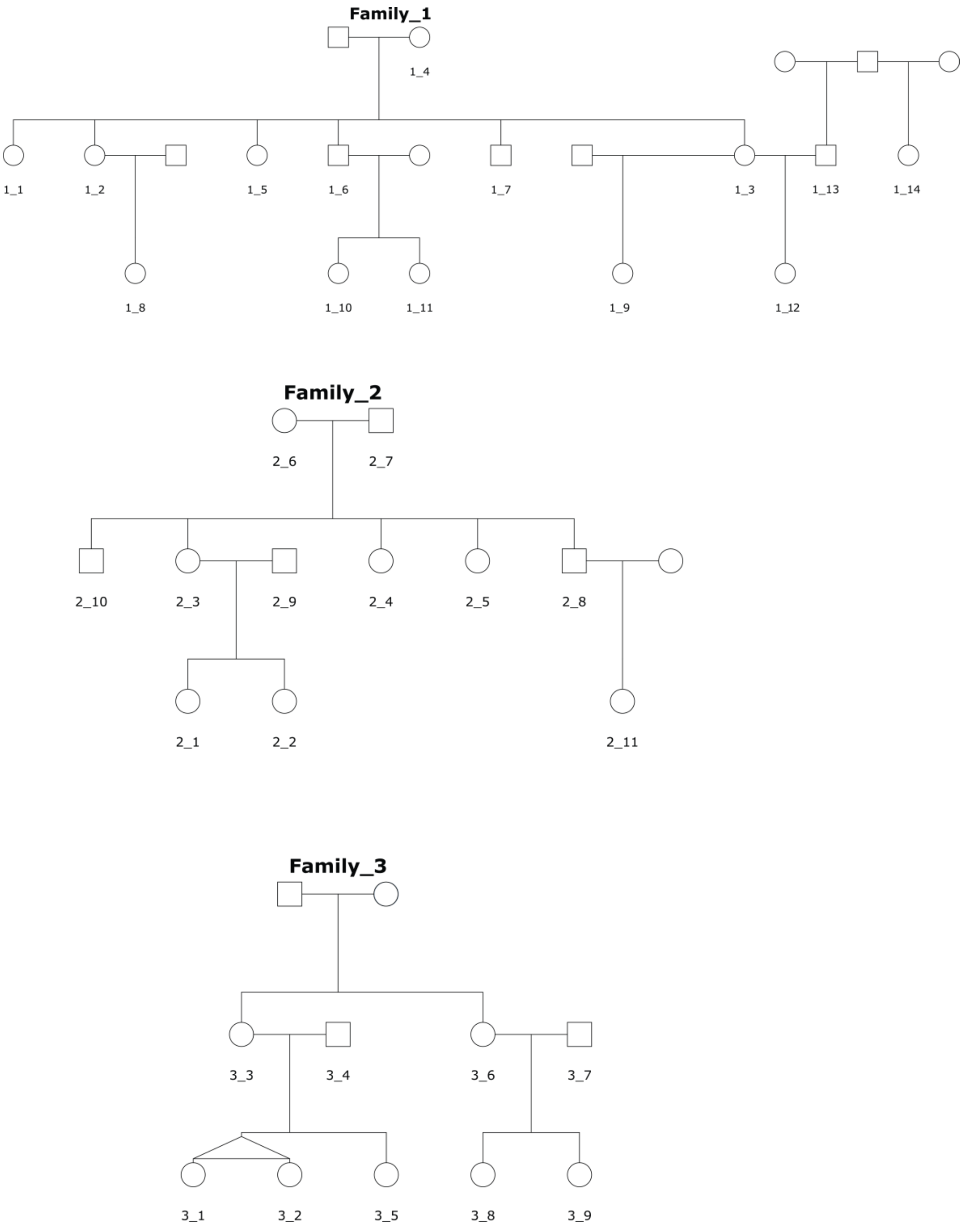

Figure S1 (continued)

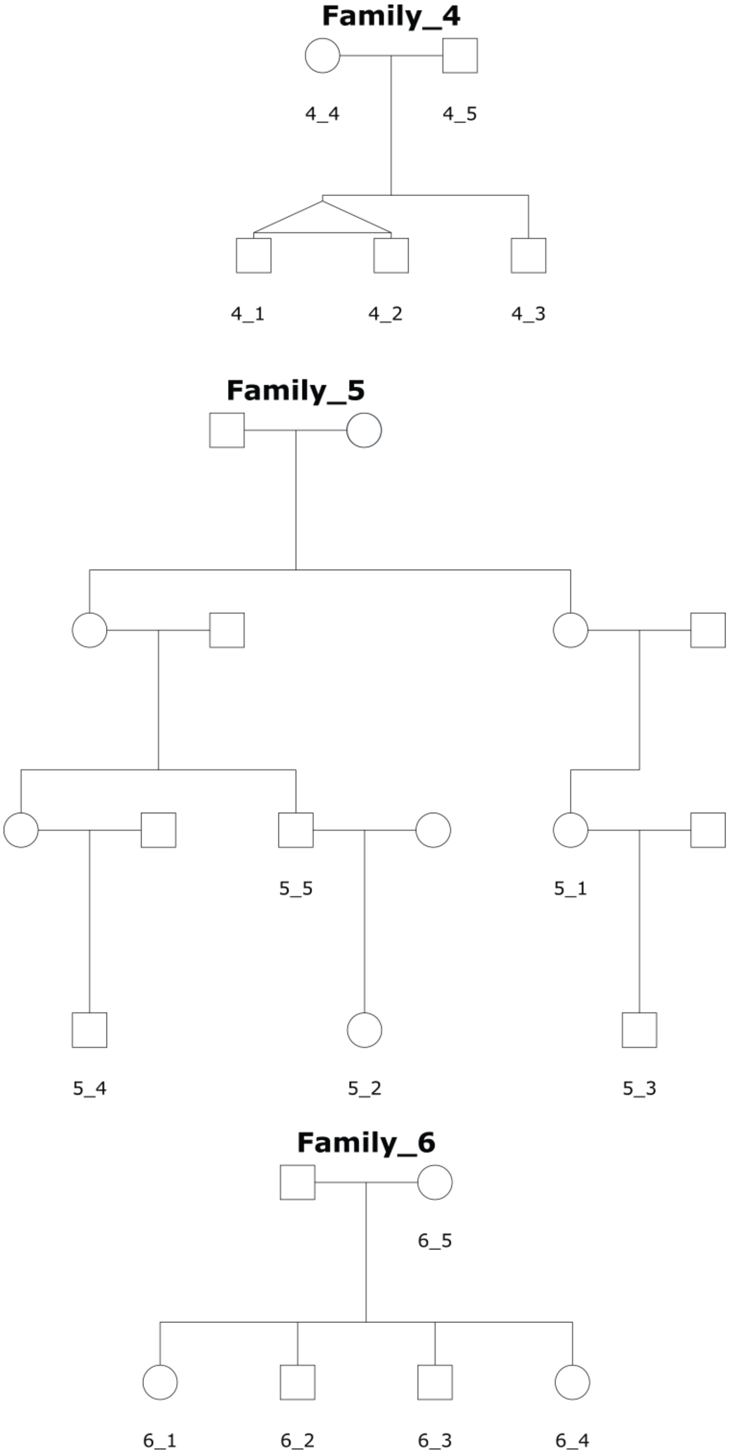

Figure S1 (continued)

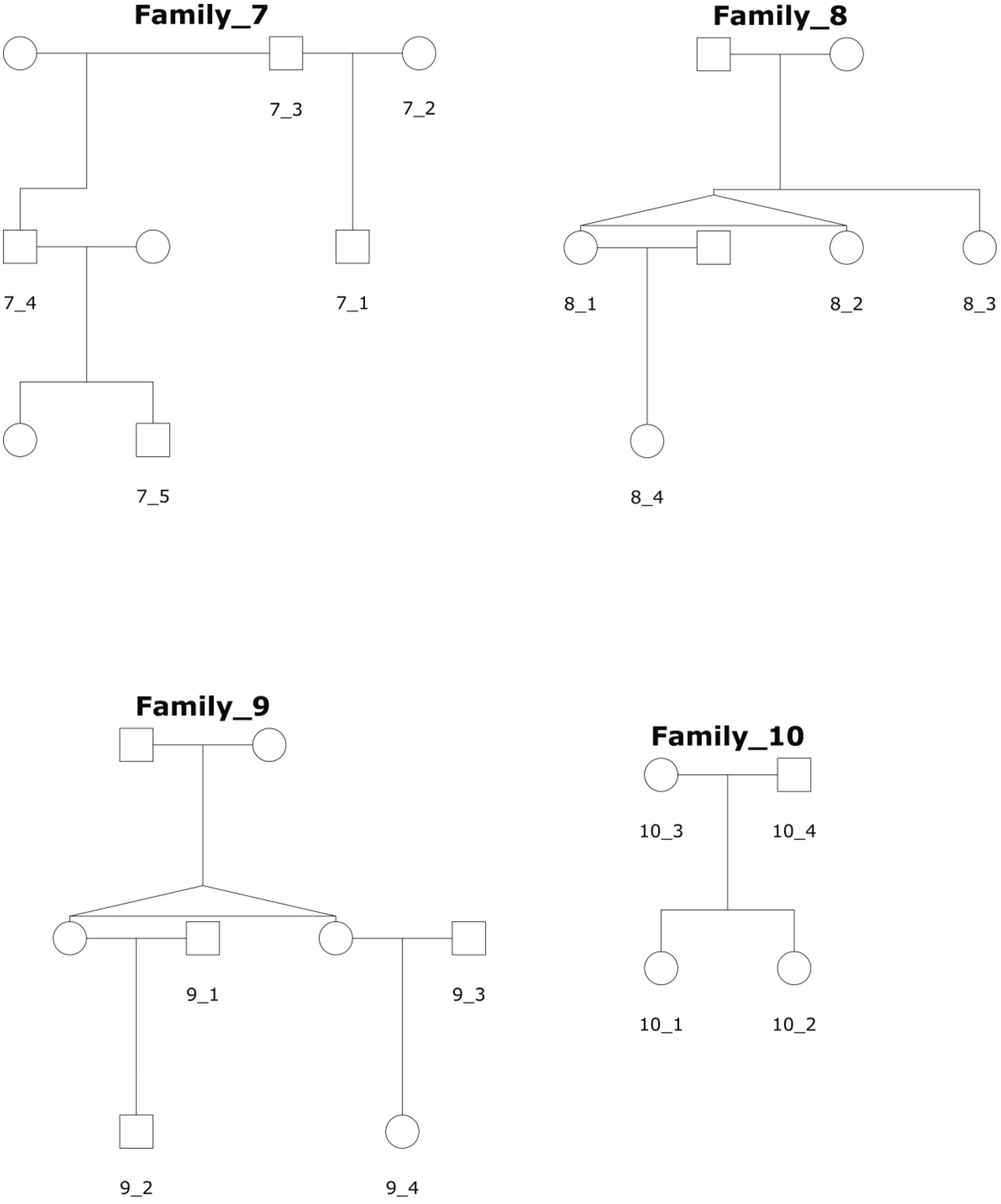

Figure S1 (continued)

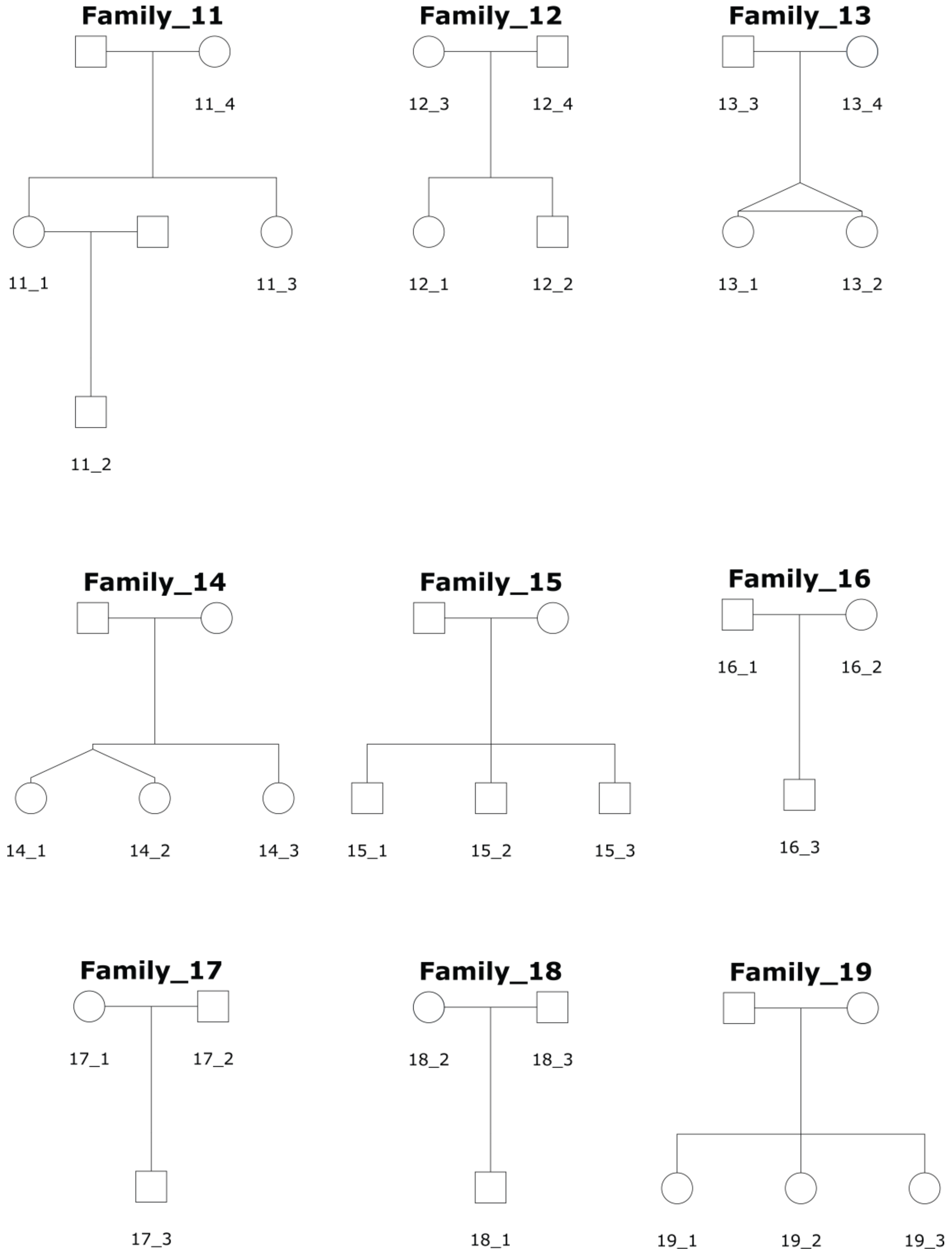

Figure S1 (continued)

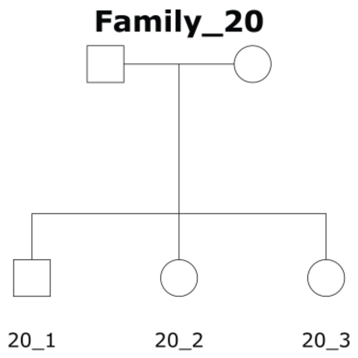

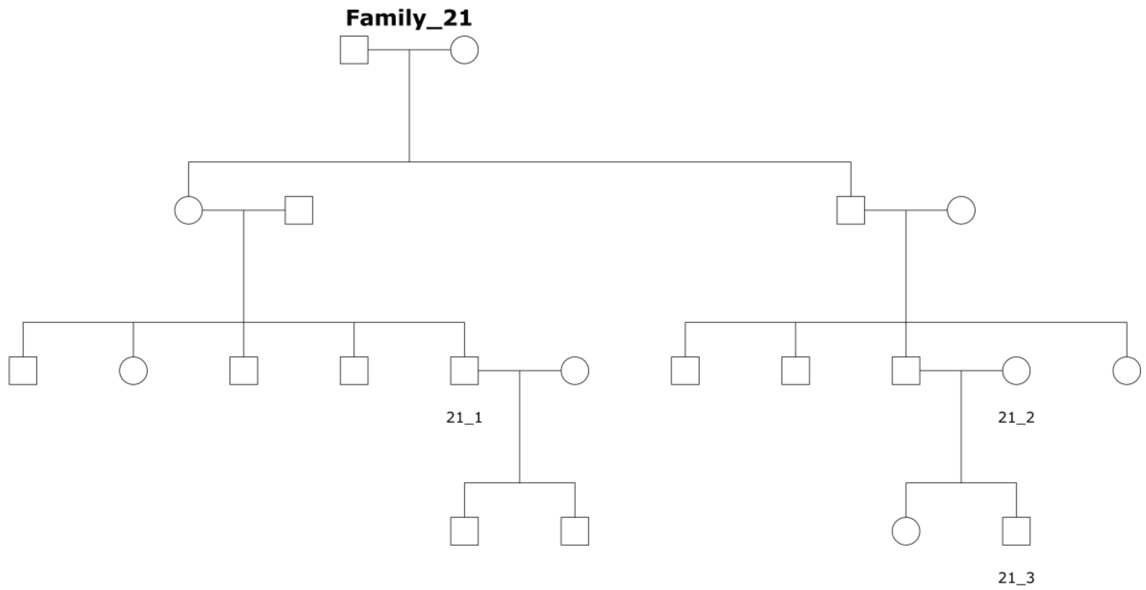

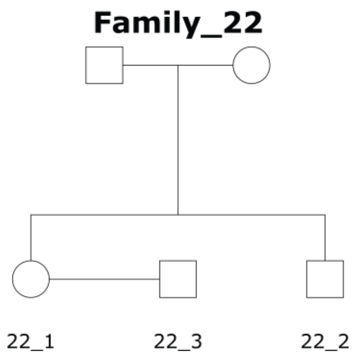

Figure S1 (continued)

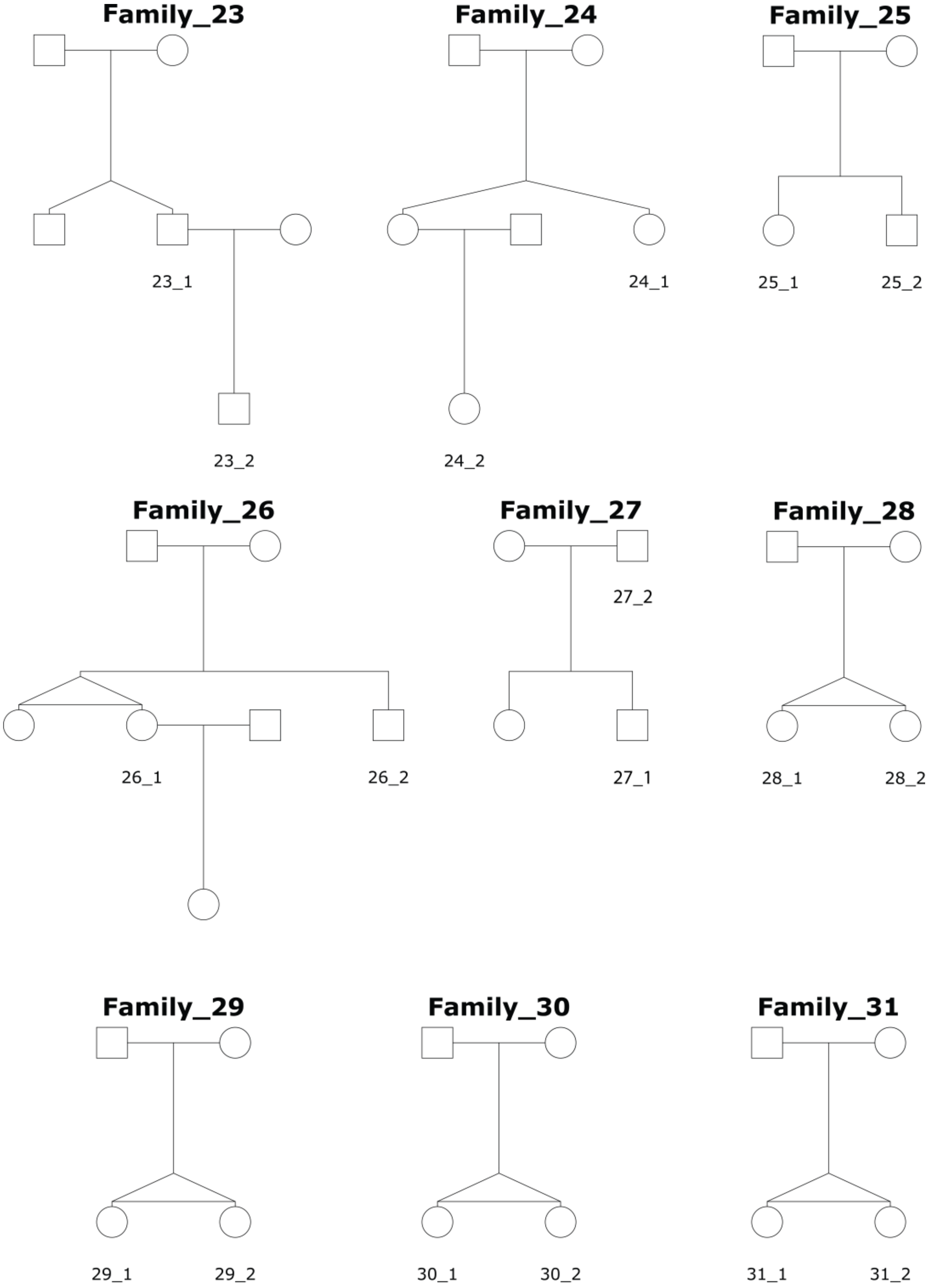

Figure S1 (continued)

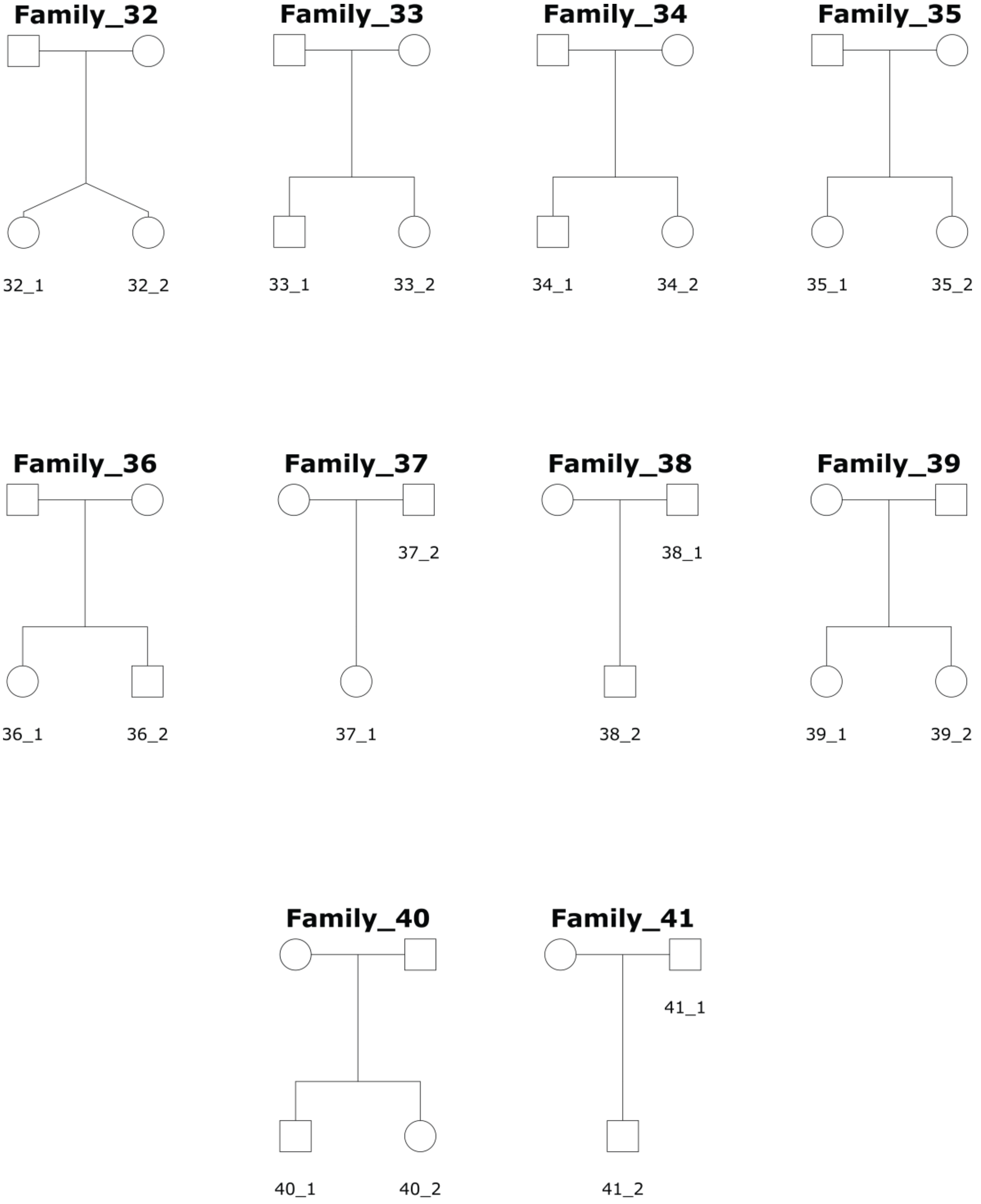

Family relatedness recorded through questionnaires was translated into pedigree diagrams for all subjects with at least one other family member in the cohort. The numbered individuals in each pedigree are the subjects for which iPSC lines were derived (see Table S1A for additional

phenotype data). Monozygotic twin pairs are drawn with a triangle, while dizygotic twin pairs are drawn with angled lines.

Figure S2. Analysis of pluripotent marker expression in iPSC lines by flow cytometry. Related to Figure 2.

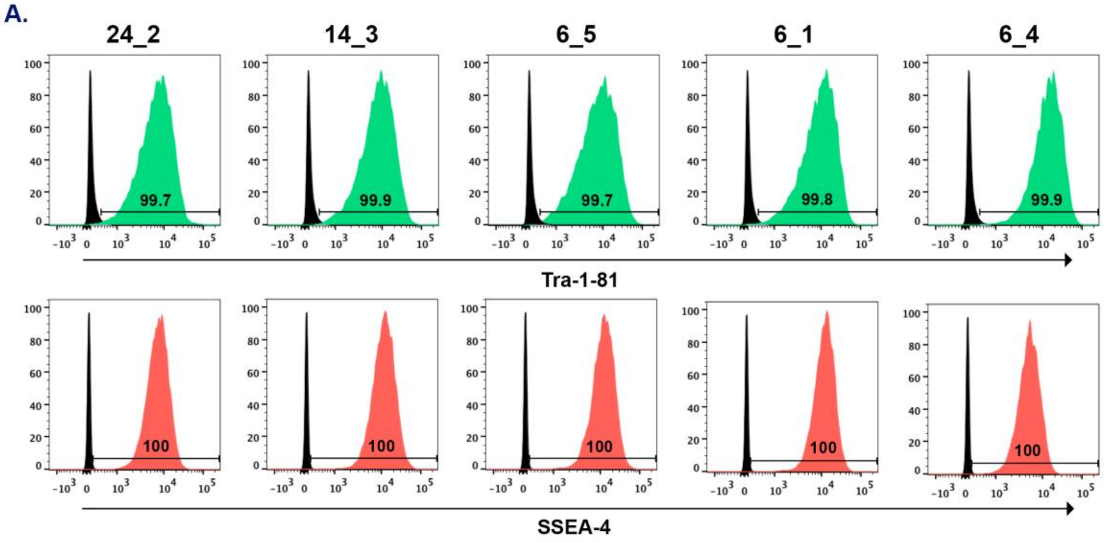

B.

Cell Line ID	% SSEA-4	%TRA-1-81
CARDiPS_24_2_iPSC_C4_P12	100.0	99.7
CARDiPS_14_3_iPSC_C2_P12	100.0	99.9
CARDiPS_6_5_iPSC_C1_P12	100.0	99.7
CARDiPS_6_1_iPSC_C2_P12	100.0	99.8
CARDiPS_6_4_iPSC_C3_P12	100.0	99.9
CARDiPS_14_1_iPSC_C3_P12	100.0	97.7
CARDiPS_6_2_iPSC_C5_P12	99.7	99.9
CARDiPS_2_11_iPSC_C2_P12	99.6	98.9
CARDiPS_7_5_iPSC_C9_P12	99.3	99.3
CARDiPS_15_3_iPSC_C3_P12	100.0	99.6
CARDiPS_15_1_iPSC_C6_P12	99.8	99.4
CARDiPS_7_4_iPSC_C6_P12	97.3	99.1
CARDiPS_6_3_iPSC_C6_P12	99.9	99.0
CARDiPS_17_1_iPSC_C5_P12	99.9	98.0
CARDiPS_15_2_iPSC_C2_P12	100.0	99.8
CARDiPS_11_4_iPSC_C4_P12	99.9	99.9
CARDiPS_14_2_iPSC_C5_P12	100.0	99.8
CARDiPS_12_3_iPSC_C3_P12	100.0	99.7
CARDiPS_4_3_iPSC_C1_P12	100.0	99.9
CARDiPS_2_1_iPSC_C4_P12	100.0	99.9
CARDiPS_2_3_iPSC_C5_P13	100.0	100.0
CARDiPS_7_3_iPSC_C3_P12	99.7	98.7
CARDiPS_11_3_iPSC_C5_P12	99.9	99.4
CARDiPS_4_4_iPSC_C4_P13	99.9	95.7
CARDiPS_12_1_iPSC_C2_P12	99.9	99.5

Cell Line ID	% SSEA-4	%TRA-1-81
CARDiPS_17_2_IPSC_C2_P12	100.0	99.6
CARDiPS_7_2_iPSC_C2_P12	99.8	99.8
CARDiPS_11_1_iPSC_C2_P12	100.0	99.9
CARDiPS_2_6_iPSC_C6_P12	100.0	95.0
CARDiPS_19_3_iPSC_C7_P13	98.4	99.0
CARDiPS_2_9_iPSC_C5_P12	100.0	96.6
CARDiPS_18_2_iPSC_C5_P12	100.0	95.2
CARDiPS_18_3_iPSC_C3_P12	100.0	99.3
CARDiPS_30_1_iPSC_C3_P13	100.0	99.9
CARDiPS_30_2_IPSC_C5_P13	99.9	100.0
CARDiPS_12_2_IPSC_C1_P12	100.0	98.7
CARDiPS_11_2_iPSC_C4_P12	100.0	99.8
CARDiPS_19_2_iPSC_C7_P14	99.9	97.2
CARDiPS_2_4_iPSC_C2_P13	98.3	97.3
CARDiPS_3_1_iPSC_C2_P19	99.8	99.4
CARDiPS_3_2_iPSC_C11_P19	100.0	98.5
CARDiPS_34_2_iPSC_C2_P13	99.7	95.5
CARDiPS_36_2_iPSC_C2_P13	99.9	99.3
CARDiPS_36_1_iPSC_C1_P13	99.9	95.9
CARDiPS_2_7_iPSC_C3_P13	100.0	99.5
CARDiPS_8_1_iPSC_C3_P13	99.9	95.6
CARDiPS_8_2_iPSC_C6_P13	100.0	99.8
CARDiPS_32_1_iPSC_C3_P14	99.7	98.1
CARDiPS_56_1_iPSC_C3_P13	99.5	95.0
CARDiPS_62_1_iPSC_C3_P13	99.3	95.0

Flow cytometry analysis of the cell surface pluripotency markers Tra-1-81 and SSEA-4 was performed in a subset of the iPSCORE iPSC lines (50 total). (A) An example of the type of analysis performed is shown for five of the iPSC lines. The individual from which the iPSC line is derived is indicated by the subject id shown at the top. (B) The percentages of cells that were positive for each individual marker are summarized, demonstrating that all iPSC lines had >95% positive expression for both pluripotent markers (Tra-1-81 and SSEA-4).

Figure S3. Distribution of CNVs across the genome. Related to Figure 3.

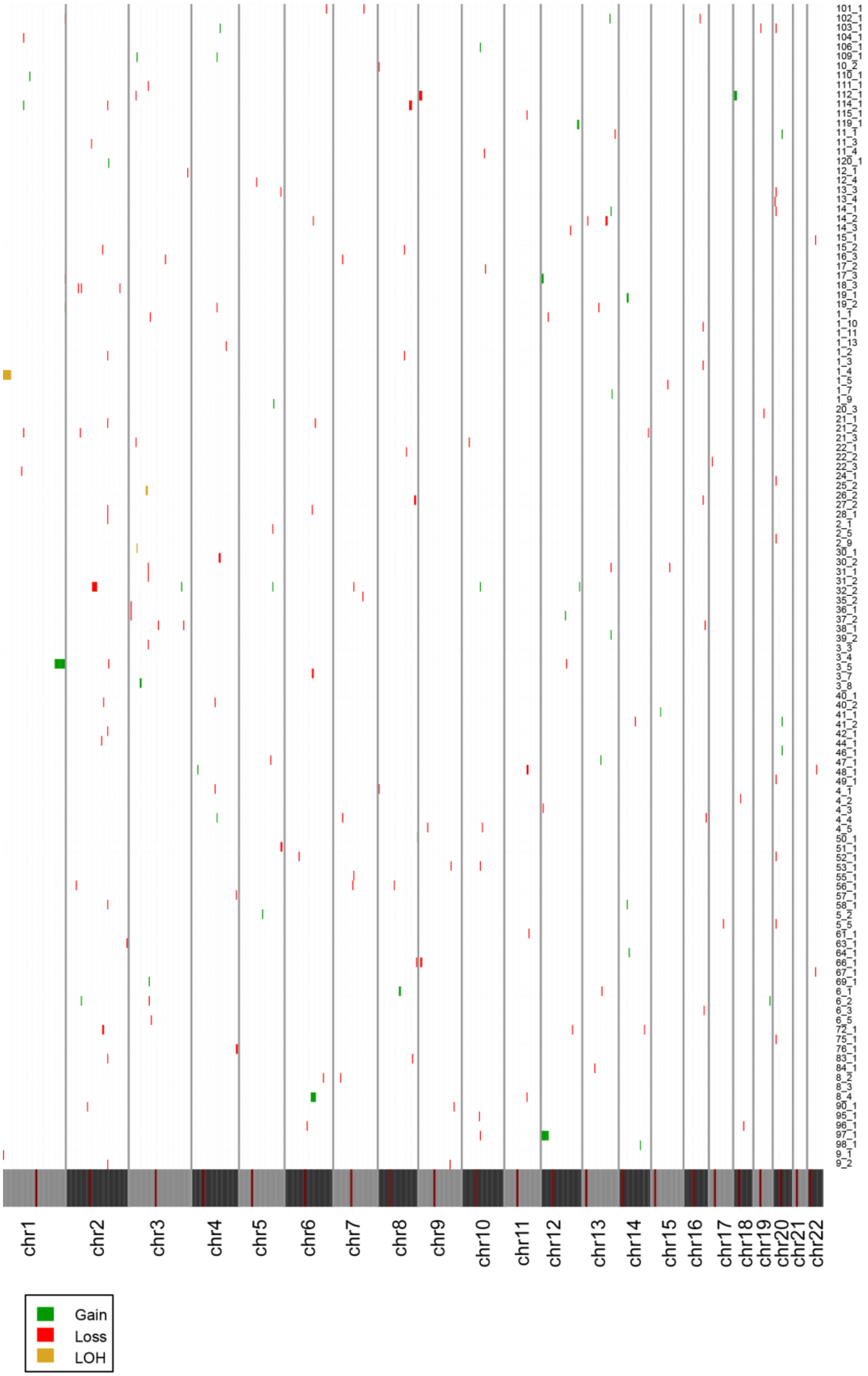

Heatmap showing genomic positions (columns) in the 121 iPSC lines that harbor at least one CNV (rows). Colors refer to different types of alterations, as indicated. The individual from which the iPSC line is derived is indicated by the subject id shown at the right. See Table S3A for coordinates of CNVs.

Figure S4. Multi-electrode array (MEA) analysis of cardiomyocytes (CM) differentiated from three iPSC lines. Related to Figure 4.

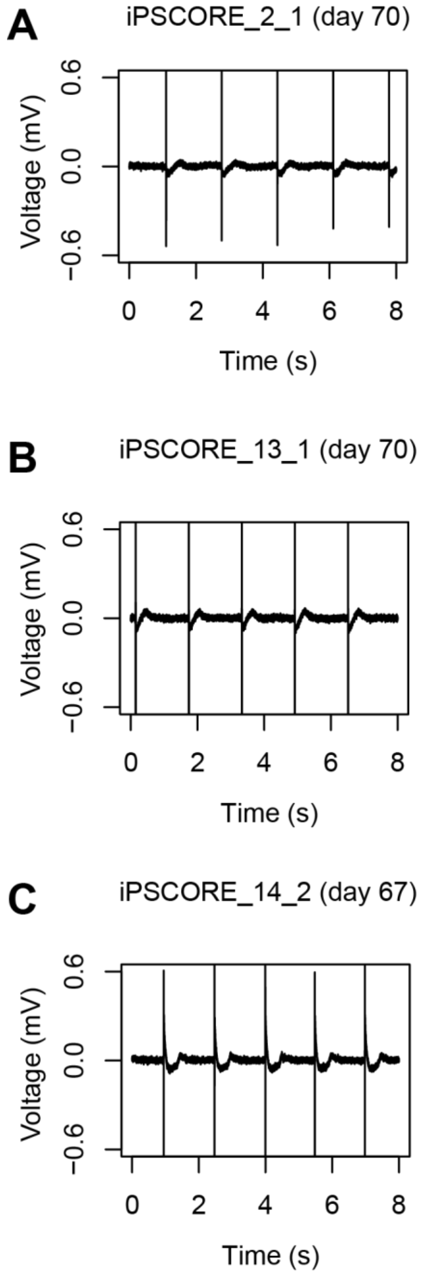

Field potential measured using MEA iPSC-derived cardiomyocytes: (A) iPSCORE_2_1; (B) iPSCORE_13_1; and (C) iPSCORE_14_2.

Figure S5. Analysis of Sendai virus clearance in iPSC lines. Related to Figure 2.

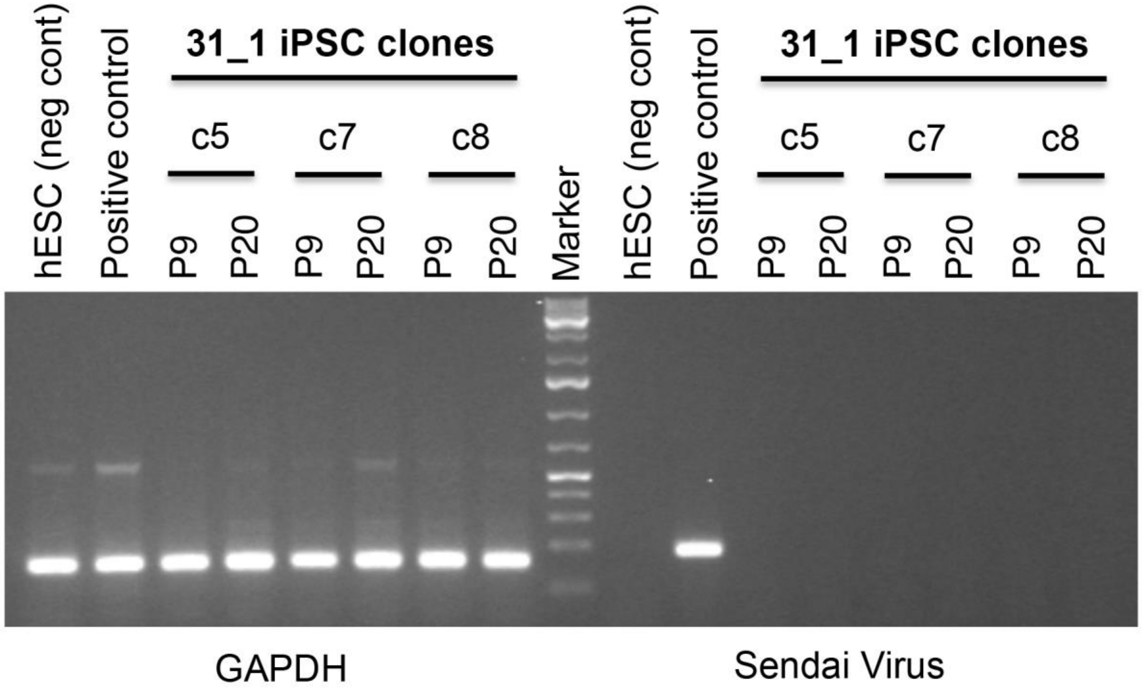

RT-PCR analysis of Sendai virus and GAPDH (housekeeping gene control) levels in iPSC lines generated from individual 31_1 in the cohort. Three clonal lines (e.g. clone 5 = c5) were assessed at passage 9 (P9) and passage 20 (P20) for the presence of Sendai virus, with all clonal lines showing clearance of Sendai virus by P9. All iPSC lines in the described study were cultured and frozen at P12 or later.

SUPPLEMENTARY TABLE LEGENDS

Table S1. Phenotype information for participants in iPSCORE collected at enrollment and identifiers for cell lines and genomic data. Related to Figure 1.

Table S1A. Phenotype information for participants in iPSCORE.
iPSCORE_ID indicates family and individual number. Subject UUID is an assigned Universal Unique Identifier (UUID) for the subject. Family ID stratifies the subject with related family members. Sex and age at the time of enrollment of the subject are given. For the 39 patients recruited from the UCSD Sulpizio Cardiovascular Center, the category of heart disease is given as the primary diagnosis, other heart diagnoses, comments and disease ontology code. Self-reported race/ethnicity (column K) obtained as a free response written by the subject or physician (denoted by asterisk) was translated into one of seven groups (African American, Asian, European, Hispanic, Indian, Middle Eastern, and Multiple ethnicities reported) and defined as recorded ethnicity grouping (column L). The expected superpopulation (SP) from the 1000 Genomes Project was generated from the recorded ethnicity and compared to the observed superpopulation (column M). We observed no mismatches. Hispanic individuals were considered a match if they matched either EUR or AMR. Middle Eastern individuals were considered a match if they matched EUR, SAS, or AFR. In the case of mixed race/ethnicity, individuals were considered to match if the top match matched one of their reported race/ethnicity groups or if their position on the PCA plots (Figure 1G) was consistent with a mixture of their reported ancestries.

Table S1B. Putative genetic variants underlying cardiac diseases
Variants identified by whole genome sequencing in cardiomyopathy or arrhythmia disease-associated genes are reported. iPSCORE_ID, Subject_UUID, and Family ID are as in Table S1A. rsID is the dbSNP identifier for the variant. Chromosome, Position, Reference allele, and

Alternate allele are reported for genome build hg19. Genotype indicates the genotype for the individual (0/0 = homozygous reference, 0/1 = heterozygous, 1/1 = homozygous alternate). The Gene indicates the affected gene, with Coding sequence change and Amino acid change indicating the impact of the variant on the gene and protein, respectively. The ClinVar clinical significance lists the pathogenicity reported to ClinVar. The ClinVar RCVaccession reports the accession numbers in ClinVar for these variants.

Table S1C. Table linking identifiers for iPSCORE participants, cell lines and genomic data. The IPSCORE IDs and UUID Subject IDs are given (columns A, B). ID and passage and clone information about iPSC lines (columns C, D, E) and the WiCell ID (column F). UUID for WGS data and DNA tissue source (columns G, H). UUIDs for RNA-seq and genotype array data (columns I, J). The UUID identifiers are also referenced in the dbGaP dataset.

Table S2. Pluripotency of 213 iPSC lines calculated by PluriTest-RNAseq. Related to Figure 2.
For each of the 213 iPSC lines that underwent RNA-seq, we provide UUID Subject IDs, iPSCORE IDs, RNA-seq UUID (as deposited to dbGAP), novelty score, pluripotency score and final assessment of pluripotency as PluriTest-RNAseq (213 lines). In total, 206 lines have high pluripotency and low novelty scores calculated by PluriTest-RNAseq. The other seven have values slightly below the 98% sensitivity and 100% specificity thresholds, suggesting that, while they are likely pluripotent, additional evaluations would be needed to confirm their pluripotency. These 7 lines overall showed high genomic integrity and a subset differentiated to cardiomyocytes at a similar rate as other passing cell lines supporting their pluripotency.

Table S3: CNV analysis of the 222 iPSC lines. Related to Figure 3.

Table S3A. List of the 199 detected CNVs (see Experimental Procedures).
The iPSC name is given (column B), the CNV type (column C) and whether it is chromosomal (a chromosomal arm or full chromosome) or subchromosomal (column D). Coordinates of the detected CNV, length of CNV, and the method of detection ("Primary Detection Method") are reported. Each CNV was detected either by automatic analysis paired with a germline sample (with Nexus) or by visual analysis and manual curation. Some of the manually curated CNVs did not pass the automatic detection cutoff points in Nexus, however they displayed typical clear CNV patterns and so were retained in the final CNV set. Nexus-called CNVs that were not clear after visual inspection of the log R ratios and B-allele frequencies were removed. Segmented neighboring CNVs were merged into a single CNV as appropriate. The cytoband containing the CNV (column J) and if the CNV was in one of the five regions significantly enriched for CNVs (column K) is given.

Table S3B. iPSC lines containing CNVs at passage (P12) examined at an earlier passage (P3).
iPSCORE ID, CNV type and chromosome position are given (columns A, B, C). The clone ID for the first (column D), second (column E) and third clones (column F) from the same subject (IPSCORE ID) are given. Columns G, H and I indicate whether the CNV was present by visual inspection in the same clone at an earlier passage (P3), the second clone or third clone. A "--" indicates that no data is available.

Table S3C. Significantly recurrent CNV regions.
The chromosome coordinates, length and cytoband location of the five regions that were enriched for CNVs are given. The type of alteration, number of genes involved and minimum STAC frequency P-value are reported.

Table S4: Gene Ontology terms associated with four gene cluster groups in time course study. Related to Figure 4C.

We tested 20,178 GO terms included in GOseq v. 1.24.0 (March 30 2016) separately for each cluster described in Figure 4C, corresponding to genes active at Day 0, Day 2, Day 5 and Day 15 of cardiomyocyte differentiation. For each GO term (column A), we provide the GO branch ("Biological Process": BP; "Molecular Function": MF; or "Cellular Component": CC, column B), the GO description (column C), the analyzed day (column D), the number of genes in the cluster included in the GO term and outside the GO term (column E and F, respectively) and of all the remaining human genes, the number in and not in the cluster (column G and H). For each GO term, log2 ratio (column I) was calculated by fraction of genes included in the GO term in the cluster and not in the cluster. P-values (column J) were calculated using GOseq and adjusted using Bonferroni method (column K). GO terms significant at an FDR < 0.05 are reported.

Table S5: Genotype data from 222 germline samples at 2,571 GWAS SNPs measured by the HumanCoreExome BeadChip. Related to Figure 5.

The chromosome, coordinate and SNP ID (rsID) are listed (columns A, B, C). The reference and alternate alleles as well as the risk allele as reported by the NHGRI GWAS Catalog, associated disease/trait and PubMed ID and nearest mapped gene are given (columns D, E, F, G, H, I). When a SNP was associated with multiple traits, it is listed multiple times. The genotype of each individual is listed as the number of risk alleles (0 = non-risk/non-risk, 1 = risk/non-risk, 2 = risk/risk).

SUPPLEMENTAL EXPERIMENTAL PROCEDURES

Cell culture and human iPSC generation

Cultures of primary dermal fibroblast cells were generated by mechanical dissection and enzymatic digestion of the punch biopsy tissue, followed by adherent outgrowth on gelatin coated 24-well plates as previously described (Israel et al., 2012). The primary fibroblast cultures were expanded for approximately 3 passages prior to cryopreservation in advance of reprogramming. The fibroblasts were thawed and plated at a density of 250K cells/well of 6-well plate, then infected with the Cytotune Sendai virus (Life Technologies) per manufacturer's protocol to initiate reprogramming. The Sendai infected cells were maintained with 10% FBS/DMEM (Invitrogen) for Days 4-7 until the cells recovered and repopulated the well. These cells were then enzymatically dissociated using TrypLE (Life Technologies) and seeded onto a 10-cm dish pre-coated with mitotically inactive-mouse embryonic fibroblasts (MEFs) at a density of 500K/dish and maintained with hESC medium, as previously described (Ruiz et al., 2010). Emerging iPSC colonies were manually picked after Day 21 and maintained on Matrigel (BD Corning) with mTeSR1 medium (Stem Cell Technologies) as previously described (Panopoulos et al., 2012). Multiple independently established iPSC lines (i.e. referred to as clones) were derived from each individual (on average three clones), with a minimum of two clones frozen at passage three as backup stocks, and one clone cultured to late passage (typically passage 12) before freezing ten vials for banking at WiCell Research Institute (WiCell IDs in Table S1C). Sendai virus clearance typically occurred at or before P9, and was not detected in the iPSC lines at the P12 stage of cryopreservation (Figure S5).

Sendai Virus Clearance Assessment

Total RNA was harvested from iPSC lines at indicated passages using Trizol Reagent (Invitrogen). For the positive control, RNA was isolated from fibroblasts 3 days post Sendai virus infection. RNA harvested from human embryonic stem cells (ESCs) was used as an uninfected pluripotent control. Collected RNA was reverse transcribed using the SuperScript II Reverse Transcriptase kit (Invitrogen) according to manufacturer's protocol. PCR was performed using primers to Sendai virus as described in the Cytotune Sendai Virus kit (Life Technologies). GAPDH (primers described in (Panopoulos et al., 2011)) was used as an internal loading control.

Flow Cytometry Analysis

Fifty iPSC lines were evaluated by flow cytometry for pluripotent marker expression. Prior to freezing, cells were brought to approximately 60% confluence and individualized with TrypLE (Thermo Fisher). After washing, sites were blocked using Fcblock (Biolegend) for 30 minutes. Cells were then resuspended in buffer (1% BSA/PBS) and stained using Tra-1-81 (Alexa Fluor 488 anti-human, Biolegend), SSEA-4 (PE anti-human, Biolegend), or the appropriate isotype controls for one hour at RT. Cells were resuspended in flow buffer (1%BSA/PBS) and analyzed using a BD FACSCanto Flow Cytometer (10,000 events counted) and the FACSDiva software (BD). They were scored as pluripotent if they were found to be 95% positive for both Tra-1-81 and SSEA-4. Pluripotency was also examined using RNA-seq data (see below).

RNA-Seq

Total RNA was extracted from pellets of $1x10^6$ cells frozen in RLT plus buffer in the Qiagen AllPrep DNA/RNA Mini kit (Qiagen Cat# 80204) and eluted in molecular grade H_2O. RNA concentration was measured by Nanodrop and integrity was determined using the Agilent 2200 TapeStation System. A total of 213 mRNA libraries were prepared by Illumina Truseq Stranded and sequenced by HiSeq2500, to an average of 20M 100bp read-pairs per sample. Reads were

aligned using STAR (2.5.0a) to the hg19 reference and a splice junction database built from the Gencode v19 gene annotation (Harrow et al., 2012). Duplicates were marked using biobambam2 (2.0.21) and transcript and gene-based expression values, including read count and transcripts per million (TPM), were obtained using the package RSEM (Li and Dewey, 2011). Read counts were normalized using variance stabilizing transformation (VST) using DeSeq2 (Love et al., 2014). VST-normalized expression levels were transformed to Z-scores by subtracting the mean value of each gene and dividing by the standard deviation.

To examine the similarity of the iPSCs to other iPSCs, embryonic stem cells, and fibroblasts, we extracted the expression levels of 34 genes known to be relevant based on the TaqMan hPSC Scorecard Assay (Choi et al., 2015), and compared their expression profiles between our 213 iPSCs and 73 publicly available cell lines from the Gene Expression Omnibus (GEO) series GSE73211 (21 iPSCs, 35 hESCs and 17 fibroblasts) (Choi et al., 2015) by hierarchical clustering and generating a heatmap using the Pheatmap R package (Figure 2A).

For the cardiomyocyte differentiation time course experiment, RNA for three iPSC lines (2_2, 2_3 and 2_9) was collected in biological triplicates at day 2, 5, 9 and 15 (Paige et al., 2012). The 500 autosomal genes with highest standard deviation in expression levels were used for hierarchical clustering and to generate a heatmap. Four gene groups, roughly corresponding to genes active in iPSC and in cells at day 2, 5 and 15, were determined using the function cutree (k = 4) in R. Functional enrichment for each group was determined using GOseq v. 1.24.0 (March 30 2016) (Young et al., 2010) on 20,178 GO terms including 20,345 human genes. P-values from GOseq were adjusted for multiple testing hypothesis using the Bonferroni method (Table S4).

PluriTest-RNAseq

PluriTest-RNAseq uses an extended and modified version of the array based PluriTest workflow (Muller et al., 2011). This algorithm generates a Pluripotency score that is the result of a logistic regression model that measures the probability of a line to be pluripotent, as well as a Novelty score that indicates the deviation of an iPSC line from a normal pluripotent line, with larger values indicating gene expression patterns usually not observed in iPSC. According to the PluriTest algorithm, high quality pluripotent lines have Pluripotency Score ≥ 20 and Novelty Score ≤ 1.67. These thresholds allow us to label a sample as "pluripotent" (Muller et al., 2011). The critical update and modification to the PluriTest procedure is the construction of a TPM (Transcripts Per Kilobase Million) based "virtual array" for each sample. Testing PluriTest-RNAseq with RNA-seq data from pluripotent and non-pluripotent cell lines reveals that it functions with similar level of specificity and selectivity as the original PluriTest array-based procedure. A complete account will be described in full elsewhere (manuscript in preparation FM, RW, BS, JL).

Briefly, the 213 samples were processed using the pseudo aligner Salmon version 0.7.2 (Patro et al., 2015) against the GRch38.p7 Gencode v25 transcriptome sequences (gencodegenes.org). A "virtual array" probe set was generated by locating the exact match probe sequences from the HT12v4 Illumina array in the Gencode v25 transcriptome sequences. This "virtual array" probe set was pruned for probes with either no match in the Gencode v25 transcriptome, or that had large model errors. We assessed the error in the "virtual array" model by performing a t-test between the expression in pluripotent samples of GSE53094 (processed as above) and the pluripotent samples in the original training set. Thus, probes with no hits in Gencode v25 or with a foldchange >0.5 and a p.value < 0.05 according to the t-test were removed, leaving 10,079 probes. A sample "virtual-array" was created by summing the Salmon

TPM for transcripts with matches to each of these 10,079 probe sequences. As previously described (Muller et al., 2011), the data was then transformed into a standard R-lumiBatch object, quantile normalized, and tested with the predictive model. This yields the pluripotency score and novelty score which reflect how similar an iPSC is to those in the original data model. Variations in the probes used can create some subtle scoring differences between PluriTest-RNAseq and PluriTest.

Previously, we set the Pluripotency and Novelty Score thresholds for the array based version of PluriTest to separate high quality iPSC lines from those with quantifiable deviations from the pluripotent phenotype (e.g. germ cell tumor cell lines and parthenogenetic stem cell lines) with 98% sensitivity and 100% specificity (Müller 2011). Cell lines that have unusually high novelty scores indicate that these test samples should be additionally evaluated for epigenetic or genetic abnormalities or unwanted differentiation. Cell lines that have pluripotency scores just below the cutoff threshold may need further investigation to confirm pluripotency. In this manuscript, for cell lines not passing either threshold, copy number estimation based on the genotype array analysis were examined to rule out genetic abnormalities and cardiomyocyte differentiation was examined to support pluripotency.

HumanCoreExome array processing and selection of SNPs

Genomic DNA was isolated from iPSC lines (AllPrep DNA/RNA Mini Kit, Qiagen) and from blood or fibroblast germline samples (DNEasy Blood & Tissue Kit), normalized to 200 ng, hybridized in pairs to Illumina HumanCoreExome arrays (Illumina), and stained per Illumina's standard protocol. The stained Beadchips were then scanned on the Illumina HiScan and processed in GenomeStudio (v 1.9.4). Genotypes were converted from Illumina TOP orientation to genome orientation (b37) using the humancoreexome-12v1-1_a-b37-strand and

HumanCoreExome-24v1-0_A-b37-strand files generated through the Wellcome Trust Center for Human Genetics (http://www.well.ox.ac.uk/~wrayner/strand/). Sites reported as “Cautious Sites” (http://genome.sph.umich.edu/wiki/Exome_Chip_Design#Cautious_Sites) were removed. Sites were annotated to dbSNP 138 identifiers using The Genome Analysis Toolkit (GATK) (DePristo et al., 2011). We observed an average call rate of 99.2% across the 444 arrays.

To examine family relationships, estimate ancestry, and to confirm iPSC sample identity by comparison with the matched germline sample, we used a subset of the array SNPs comprised of 90,099 SNPs that were in linkage equilibrium ($r2 < 0.2$), common ($MAF > 0.05$), and present by dbSNP rsID in the KGP Phase 3 data (downloaded from ftp://ftp.1000genomes.ebi.ac.uk/vol1/ftp/release/20130502/).

Family relatedness

Genotypes from individuals that were part of a family with two or more people were compared to determine if the reported relationships matched the kinship coefficient (PI_HAT) calculated using PLINK (Purcell et al., 2007) (v1.09). Pairs of individuals that differed in expected and reported kinship coefficient by more than 0.1 were investigated and compared to other pairwise measures within the family when available to verify unexpected relationships. We observed four pairs with higher deviations from expected, but these relationships were verified through relationships within the family. These pairs all included individuals of admixed ancestry, which is known to bias measures of pIBD (Thornton et al., 2012).

Ancestry estimation

We estimated the ancestry of each participant by comparing their genomes to those of individuals in the 1000 Genomes Project (KGP) using a previously published approach (Smith et al., 2014). We identified the KGP super population group (AFR: African, AMR: Admixed

American, EAS: East Asian, EUR: European, SAS: South Asian) to which each iPSCORE participant was most similar. First, KGP individuals were clustered using principal components analysis (--pca in PLINK) and iPSCORE individuals were mapped onto these components by applying the "--within" command. To identify which super population the iPSCORE individual best matched, we used using linear discriminant analysis (lda command in MASS package (Venables et al., 2002) in R) with the first 20 principal components of the KGP individuals as a training set. We compared predicted groups to self-reported ethnicity, classifying recorded ethnicity groupings by the super population most likely to match. We considered a match for the following pairs: African American – AFR; Asian – EAS; European – EUR, Hispanic – EUR or AMR; Indian – SAS; Middle Eastern -- EUR, SAS, or AFR. In the case of mixed race/ethnicity, individuals were considered to match if the top match matched one of their reported race/ethnicity groups or if their position on the PCA plots was consistent with a mixture of their reported ancestries.

Putative genetic variants underlying cardiac diseases

To identify genetic variants that could potentially be associated with the reported cardiac diseases, we examined whole genome sequence data (DeBoever et al., In Press) (Table S1B). For probands with cardiac disease and their family members, we examined genetic variation within genes that are part of the GeneDx Cardiomyopathy or Arrhythmia Panels (www.genedx.com) for individuals with reported cardiomyopathy or arrhythmia diseases, respectively. The resulting genotypes were annotated using ClinVar (Landrum et al., 2016) and variants associated with "pathogenic" or "likely pathogenic" reports were examined. Variants that were previously reported as pathogenic, but were later reported as "benign" or "likely benign" by GeneDx were

excluded. Variants that were not reported to be associated with the proband's reported disease or did not segregate with disease in the family were also excluded.

Confirming iPSC sample identity and genetic sex

All iPSC lines were compared to their respective germline sample using the –genome command in PLINK and samples were flagged if the kinship coefficient (PI_HAT) was less than 0.95. Genetic sex was estimated using the command "--check-sex" in PLINK and compared to self or physician report.

Copy Number Variation Determination

Raw scan data were processed by Genome Studio (Illumina, Inc) using the supplied clusterfiles for SNP calling on the Human Core Exome arrays (average call rate 0.99, GenCall threshold 0.15). Processed SNP array data for the 222 iPSCs were subjected to both manual and computerized analysis for somatic CNVs. For computerized analysis, genotype data were exported to Nexus CN (version 7.5) where CNV calling was carried out with the hg19/GRCh37 reference version of the human genome. The X and Y chromosomes were removed due to the complexity of reliably determining copy number in these copy variable and highly repetitive chromosomes. A descriptor sheet was supplied with the 222 sample pairings for germline to corresponding iPSC results files. The Nexus files and settings used were: Systematic Correction File: Catlg_ILM_HumanCoreExome-12v1-1_B_20140311.bed_hg19_ilum_correction.txt (as supplied by Biodiscovery Inc), Recenter Probes to Median, Analysis performed with the SNPRank Segmentation algorithm. Significance threshold 5.0E-9, Min Number of probes per segment = 7, High Gain 0.75, Gain 0.22, Loss -0.2, Big Loss -1.1. Called CNVs were size filtered with those <100kb removed. This is the conservative limit of detection for CNVs when 7 probes are used with a spacing (90% of probes) of 14.3kb. CNV regions called LOH were

excluded if they also were listed as copy number loss, resulting in 272 regions. We then performed systematic manual inspection of each Nexus called CNV, visualizing the B-allele frequencies (proportion of A and B alleles at each genotype) and log R ratios (ratio of observed to expected intensities) for each iPSC and its respective germline sample. These plots were visually scanned by a trained operator and Nexus called CNVs that were not visually consistent with a CNV based on B-allele frequencies and log R rations were removed. In addition, manual inspection of the entire genome (including sex chromosomes) was performed for each sample compared to the respective germline. This complementary approach, which is good for calling large CNVs, identified 31 CNVs, of which, 10 were also called by Nexus. The Nexus Allelic-Imbalance and LOH CNV classes were combined into one group called LOH for Figures 3C and S3.

Identification of clustered CNVs

To test for significant clustering of CNVs across multiple samples, we used the STAC program (Diskin et al., 2006). Briefly, considering each chromosome independently the algorithm tries to identify a set of aberrations with a higher frequency than what is expected to occur randomly. We partitioned each chromosome into 100kb regions and indicated whether a CNV overlapped each region for each sample. We then performed 1,000 permutations for each chromosome to identify locations with CNV frequencies higher than expected by chance (frequency P-value < 0.05). Regions that were adjacent to each other were merged and the minimum P-value for the region reported (Table S3C).

Differentiation of iPSC lines into cardiomyocytes

Differentiation into cardiomyocytes was performed according to the protocol described by Lian et al. (Lian et al., 2013). For the time course experiment, three cell lines were differentiated in

three six-well plates each, and each plate represented a biological replicate. From each six-well plate, one well was harvested on day 0, 2, 5, 9, and 15, corresponding to previously described cardiac differentiation stages (Paige et al., 2012). For the molecular study of *KCNH2,* iPSC lines from seven family members were seeded to T150 flasks and differentiated into cardiomyocytes to day 15 in two independent experiments per line (biological replicates). Cells were dissociated using Accutase; one million cells per sample were lysed and stored in RLT plus buffer (Qiagen) for RNA extraction.

Immunohistochemistry and immunofluorescence

For sarcomeric alpha-actinin (ACTN1) and connexin 43 (Cx43) immunofluorescence, day 34 iPSC-CMs were cultured on 0.1 % gelatin-coated glass-bottom plates for 48-72hrs, and then fixed with 4% paraformaldehyde at room temperature (RT) for 20 mins. Fixed iPSC-CMs were permeabilized in 0.1% Triton X-100 for 8 mins at RT, then blocked in 5% bovine serum albumin for 30 mins at RT, and then incubated overnight at 4 °C with rabbit polyclonal anti-Cx43 antibody (Invitrogen, 710700, dilution 1:1,000) and mouse monoclonal anti-ACTN1 antibody (Sigma, A7811, dilution 1:200). Cells were incubated with donkey anti-rabbit Alexa Fluor 488 (Invitrogen, A-21206, dilution 1:800) and goat anti-mouse Alexa Fluor 568 (Invitrogen, A-11004, dilution 1:800**)** secondary antibodies for 45 mins at RT. Nuclei were counterstained with DAPI. Cells were washed 3x in PBS between each step. Olympus FluoView FV1000 confocal microscope was used for imaging. For myosin light chain 2a (MLC2a) staining, day 15 cardiomyocytes were seeded onto chambers with microscope slides (Millipore) and cultured overnight. After fixation with 4% PFA, cells were blocked and permeabilized for 1 h at 37 °C with 5% BSA, 5% serum, and 0.1% Triton X-100. Cells were incubated with 1:200 dilution of mouse monoclonal anti-myosin light chain 2a (MLC2a) antibody (Synaptic Systems, 311011)

overnight at 4 °C; with secondary antibody (AlexaFluor 488) for 2 h at RT; and 20 min with DAPI. Leica SP5 confocal microscope was used for imaging.

Multi-electrode array analysis (MEA)

Beating, 25 days old iPSC-derived cardiomyocyte monolayers in 6-well plates were dislodged using a cell scraper and dissociated into small clumps by gently pipetting with a 1-ml pipette. Cell clumps were re-plated in MEA plates (Axion Biosystems) previously coated with Matrigel and allowed to settle for 24-48 hours. Electrophysiological activity was then assayed and recorded for ~1 minute using MEA Maestro apparatus (Axion Biosystems). Cells were incubated with Isoproterenol 0.01µM at 37˚C immediately before the second MEA analysis. We extracted the field potential recording from the same electrode before and after treatment and plotted the traces (Figures 4E, S4). Beat periods were calculated from the traces of 8 electrodes of a well (Figure 4F) and plotted using R.

Analysis of *KCNH2* expression by qPCR

One µg of total RNA from iPSC-derived cardiomyocytes was retro-transcribed using SuperScript III First-Strand Synthesis System (Thermo Scientific) using oligo dT primers in 20 µl reactions. RT-qPCR reactions were performed in 15 µl using 3.5 µl of a 1:50 cDNA dilution using KAPA SYBR Fast qPCR Kit (KPA Biosystem) and run on LightCycler 480 (Roche). Primers were designed and tested to amplify specifically the wild-type or the mutated allele of *KCNH2* transcript (c.3003G>A) using the following primers: forward: GTGTCCAACATTTTCAGCTTCTT**G** (wt) or GTGTCCAACATTTTCAGCTTCTT**A** (mutated), reverse: AGTGGCCATGTCTGCACTC (common to wild type and mutated). These allele-specific primers were designed using the WASP tool (Wangkumhang et al., 2007). *KCNH2* C_ts were normalized to *GAPDH* (forward primer: TGTTGCCATCAATGACCCCTT,

reverse primer: CTCCACGACGTACTCAGCG) and expressed as $\Delta\Delta C_t$ with respect to the average ΔCt_S.

GWAS loci genotypes

GWAS-associated loci were downloaded from the NHGRI GWAS Catalog (https://www.genome.gov/26525384, 2/23/2015). SNPs were retained if they had a reported P-value < 5×10^{-8}; a reported risk allele of A, C, T, or G; and a current dbSNP ID. When a SNP was associated multiple times with the same phenotype, the most significant report was used. This resulted in 4,528 SNPs, 600 phenotypes, and 5,514 SNP-phenotype relationships. Of these, 2,858 SNPs were present on the HumanCoreExome BeadChip. We further excluded sites that were ambiguous (C/G or A/T SNPs), had a Hardy-Weinberg equilibrium P-value < 10^{-7} in our study, and a call rate of <90% in the germline samples, resulting in 2,517 SNPs, 487 phenotypes, and 3,350 SNP-phenotype relationships (Table S5). Genotypes from the HumanCoreExome arrays hybridized with germline DNA were then tabulated to identify the number of individuals carrying the risk/risk, risk/non-risk, and non-risk/non-risk genotypes.

REFERENCES

Choi, J., Lee, S., Mallard, W., Clement, K., Tagliazucchi, G.M., Lim, H., Choi, I.Y., Ferrari, F., Tsankov, A.M., Pop, R., *et al.* (2015). A comparison of genetically matched cell lines reveals the equivalence of human iPSCs and ESCs. Nat Biotechnol *33*, 1173-1181.

DeBoever, C., Li, H., Jakubosky, D., Arias, A., Olson, K.M., Huang, H., Biggs, W., Sandoval, E., Matsui, H., Ren, B., *et al.* (In Press). Genetic Regulation of Gene Expression in Human Induced Pluripotent Stem Cells. Cell stem cell.

DePristo, M.A., Banks, E., Poplin, R., Garimella, K.V., Maguire, J.R., Hartl, C., Philippakis, A.A., del Angel, G., Rivas, M.A., Hanna, M., *et al.* (2011). A framework for variation discovery and genotyping using next-generation DNA sequencing data. Nat Genet *43*, 491-498.

Diskin, S.J., Eck, T., Greshock, J., Mosse, Y.P., Naylor, T., Stoeckert, C.J., Jr., Weber, B.L., Maris, J.M., and Grant, G.R. (2006). STAC: A method for testing the significance of DNA copy number aberrations across multiple array-CGH experiments. Genome Res *16*, 1149-1158.

Harrow, J., Frankish, A., Gonzalez, J.M., Tapanari, E., Diekhans, M., Kokocinski, F., Aken, B.L., Barrell, D., Zadissa, A., Searle, S., *et al.* (2012). GENCODE: the reference human genome annotation for The ENCODE Project. Genome research *22*, 1760-1774.

Israel, M.A., Yuan, S.H., Bardy, C., Reyna, S.M., Mu, Y., Herrera, C., Hefferan, M.P., Van Gorp, S., Nazor, K.L., Boscolo, F.S., *et al.* (2012). Probing sporadic and familial Alzheimer's disease using induced pluripotent stem cells. Nature *482*, 216-220.

Landrum, M.J., Lee, J.M., Benson, M., Brown, G., Chao, C., Chitipiralla, S., Gu, B., Hart, J., Hoffman, D., Hoover, J., *et al.* (2016). ClinVar: public archive of interpretations of clinically relevant variants. Nucleic acids research *44*, D862-868.

Li, B., and Dewey, C.N. (2011). RSEM: accurate transcript quantification from RNA-Seq data with or without a reference genome. BMC Bioinformatics *12*, 323.

Lian, X., Zhang, J., Azarin, S.M., Zhu, K., Hazeltine, L.B., Bao, X., Hsiao, C., Kamp, T.J., and Palecek, S.P. (2013). Directed cardiomyocyte differentiation from human pluripotent stem cells by modulating Wnt/beta-catenin signaling under fully defined conditions. Nat Protoc *8*, 162-175.

Love, M.I., Huber, W., and Anders, S. (2014). Moderated estimation of fold change and dispersion for RNA-seq data with DESeq2. Genome biology *15*, 550.

Muller, F.J., Schuldt, B.M., Williams, R., Mason, D., Altun, G., Papapetrou, E.P., Danner, S., Goldmann, J.E., Herbst, A., Schmidt, N.O., *et al.* (2011). A bioinformatic assay for pluripotency in human cells. Nat Methods *8*, 315-317.

Paige, S.L., Thomas, S., Stoick-Cooper, C.L., Wang, H., Maves, L., Sandstrom, R., Pabon, L., Reinecke, H., Pratt, G., Keller, G., *et al.* (2012). A temporal chromatin signature in human embryonic stem cells identifies regulators of cardiac development. Cell *151*, 221-232.

Panopoulos, A.D., Ruiz, S., Yi, F., Herrerias, A., Batchelder, E.M., and Izpisua Belmonte, J.C. (2011). Rapid and highly efficient generation of induced pluripotent stem cells from human umbilical vein endothelial cells. PLoS One *6*, e19743.

Panopoulos, A.D., Yanes, O., Ruiz, S., Kida, Y.S., Diep, D., Tautenhahn, R., Herrerias, A., Batchelder, E.M., Plongthongkum, N., Lutz, M., *et al.* (2012). The metabolome of induced pluripotent stem cells reveals metabolic changes occurring in somatic cell reprogramming. Cell Res *22*, 168-177.

Patro, R., Duggal, G., and Kingsford, C. (2015). Accurate, fast, and model-aware transcript expression quantification with Salmon. BioRxiv.

Purcell, S., Neale, B., Todd-Brown, K., Thomas, L., Ferreira, M.A., Bender, D., Maller, J., Sklar, P., de Bakker, P.I., Daly, M.J., *et al.* (2007). PLINK: a tool set for whole-genome association and population-based linkage analyses. Am J Hum Genet *81*, 559-575.
Ruiz, S., Brennand, K., Panopoulos, A.D., Herrerias, A., Gage, F.H., and Izpisua-Belmonte, J.C. (2010). High-efficient generation of induced pluripotent stem cells from human astrocytes. PLoS One *5*, e15526.
Smith, E.N., Jepsen, K., Arias, A.D., Shepard, P.J., Chambers, C.D., and Frazer, K.A. (2014). Genetic ancestry of participants in the National Children's Study. Genome biology *15*, R22.
Thornton, T., Tang, H., Hoffmann, T.J., Ochs-Balcom, H.M., Caan, B.J., and Risch, N. (2012). Estimating kinship in admixed populations. Am J Hum Genet *91*, 122-138.
Venables, W.N., Ripley, B.D., and Venables, W.N. (2002). Modern applied statistics with S, 4th edn (New York: Springer).
Wangkumhang, P., Chaichoompu, K., Ngamphiw, C., Ruangrit, U., Chanprasert, J., Assawamakin, A., and Tongsima, S. (2007). WASP: a Web-based Allele-Specific PCR assay designing tool for detecting SNPs and mutations. BMC Genomics *8*, 275.
Young, M.D., Wakefield, M.J., Smyth, G.K., and Oshlack, A. (2010). Gene ontology analysis for RNA-seq: accounting for selection bias. Genome biology *11*, R14.
